# Synthesis and Biological Profiling of Quinolino-Fused 7-Deazapurine Nucleosides

**DOI:** 10.1021/acsomega.4c02031

**Published:** 2024-04-27

**Authors:** Marianne Fleuti, Tania Sanchez-Quirante, Lenka Poštová Slavětínská, Eva Tloušt'ová, Michal Tichý, Soňa Gurská, Petr Džubák, Marián Hajdúch, Michal Hocek

**Affiliations:** † Department of Organic Chemistry, Faculty of Science, Charles University in Prague, Hlavova 8, Prague 2 CZ-12843, Czech Republic; ‡ Institute of Organic Chemistry and Biochemistry, Czech Academy of Sciences, Flemingovo nam. 2, Prague 6 CZ-16610, Czech Republic; § Institute of Molecular and Translational Medicine, Palacky University and University Hospital in Olomouc, Faculty of Medicine and Dentistry, Hněvotínská 5, Olomouc CZ-77515, Czech Republic

## Abstract

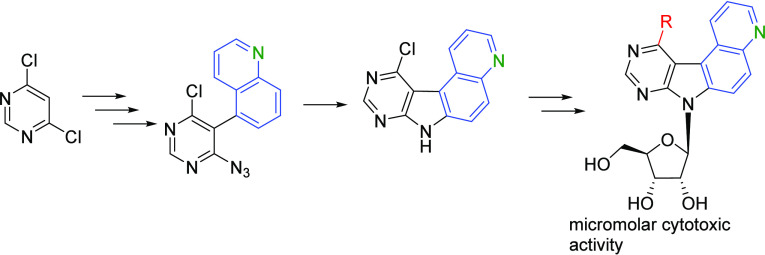

A series of quinolino-fused 7-deazapurine (pyrimido[5′,4′:4,5]pyrrolo[3,2-*f*]quinoline) ribonucleosides were designed and synthesized. The synthesis of the key 11-chloro-pyrimido[5′,4′:4,5]pyrrolo[3,2-*f*]quinoline was based on the Negishi cross-coupling of iodoquinoline with zincated 4,6-dichloropyrimidine followed by azidation and thermal or photochemical cyclization. Vorbrüggen glycosylation of the tetracyclic heterocycle followed by cross-coupling or substitution reactions at position 11 gave the desired set of final nucleosides that showed moderate to weak cytostatic activity and fluorescent properties. The corresponding fused adenosine derivative was converted to the triphosphate and successfully incorporated to RNA using *in vitro* transcription with T7 RNA polymerase.

## Introduction

Base-modified nucleosides are an important class of biologically active molecules that display antiviral,^1^ anticancer,^2^ or antiparasitic^3^ activities. Several clinically used drugs for the treatment of leukemia or tumors are based on this type of compounds.^4^ Despite the recent progress in other types of anticancer treatments,^5^ there is still a need for new types of base-modified nucleosides to find new mechanisms of action that may overcome the drug resistance and decrease toxicity.^6^

**Figure 1:**
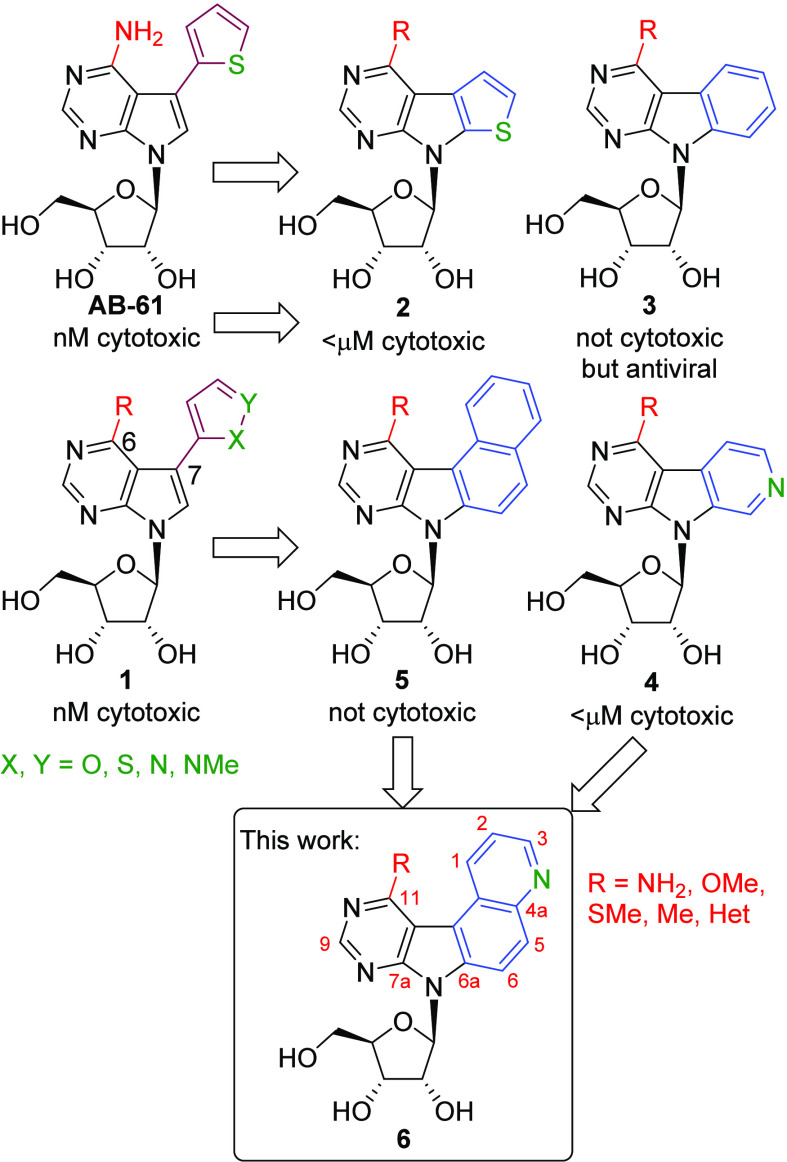
Structures and biological activity of previously synthesized substituted and fused deazapurine nucleosides and target structures of this study.

Particularly interesting are modified 7-deazapurine nucleosides, known for their broad biological activities.^7^ During our systematic research, we discovered 7-(het)aryl-7-deazapurine ribonucleosides, exemplified by 7-thienyl-7-deazaadenosine **AB-61**,^8^ which are active against a broad spectrum of cancer cell lines and show excellent selectivity against nonmalignant cells. Investigation of its mechanism of action revealed that it is phosphorylated only in cancer cells to ribonucleoside triphosphate, which is then incorporated to DNA, where it causes double-strand breaks leading to apoptosis.^9^ Later on, we found^10^ that even other 6-substituted analogues **1** bearing methoxy, methylsulfanyl, methylamino, dimethylamino, or methyl groups at position 6 retain a similar level of cytotoxic activity. Then we studied diverse deazapurines with fused aromatic or heterocyclic rings and found that the furo-^11^ or thieno-fused^12^ 7-deazapurine nucleosides **2** are also very potent cytostatics, whereas the corresponding benzo-fused analogs (pyrimidoindoles) **3**(13) are noncytotoxic but exert moderate antiviral activity. Introducing a single nitrogen atom into a specific position on the fused phenyl ring gave pyrido-fused derivatives **4**,^14^ which showed submicromolar cytotoxic activity and a similar mechanism of action involving DNA damage and apoptosis. When we increased the size of the heteroaromatic nucleobase and prepared tetracyclic naphtho-fused **5**(15) or even some bulkier pentacyclic^16^ deazapurine nucleosides, they showed only weak cytotoxic activity. To further investigate if the introduction of a nitrogen atom into the fused tetracyclic ring-system can improve the cytotoxic activity and to extend the SAR of this class of compounds, we designed and synthesized a series of novel quinolino-fused 7-deazapurine ribonucleosides (**6**) (Figure 1).

## Results and Discussion

### Chemistry

**Scheme 1:**
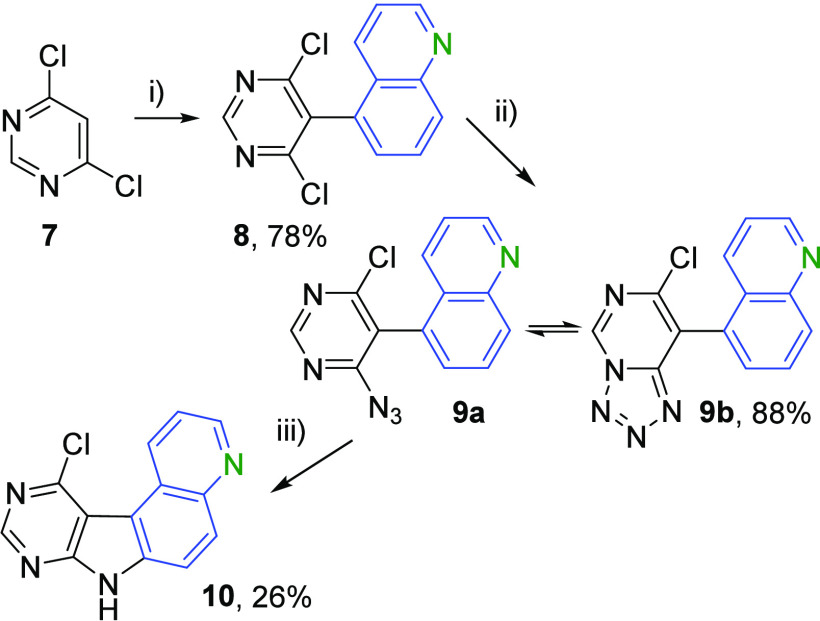
Synthesis of Key Intermediate **10** Reagents and conditions: (i) (1) (TMP)_2_Zn·2MgCl_2_·2LiCl, THF, 0 °C, 1 h, then 20 °C, 1 h; (2) 5-iodoquinoline, Pd(PPh_3_)_4_, THF, 65 °C, 24 h; (ii) NaN_3_, LiCl, DMF, 20 °C, 24 h; (iii) pyrene, UV-light (254 nm, 4 W), THF, 20 °C, 72 h.

Based on our previous experience with related fused deazapurine heterocycles^11,12,15,16^ and the availability of starting materials, we proposed a three-step reaction sequence to access the desired quinolino-fused deazapurine key intermediate **10**. The approach was based on the Negishi coupling of the zincated 4,6-dichloropyrimidine with 5-iodoquinoline and nucleophilic azidation followed by thermal, metal-catalyzed, or photochemical cyclization. (Scheme 1).

In the first step, the Turbo-Hauser base (TMP)_2_Zn·MgCl_2_·LiCl (TMP = 2,2,6,6-tetramethylpiperidyl) was generated *in situ* from (TMP)MgCl·LiCl and ZnCl_2_ followed by the addition of 4,6-dichloropyrimidine **7** to form the corresponding 5-zincated 4,6-dichloropyrimidine intermediate,^17^ which then underwent the Negishi cross-coupling with 5-iodoquinoline in the presence of Pd(PPh_3_)_4_ in THF at 65 °C for 24 h. The initial conditions, which have been successfully used in our group previously,^12^ use 1.0 equiv of **7** and 1.1 equiv of 5-iodoquinoline, resulting in **8** with 66% yield and the recovery of 21% of starting 5-iodoquinoline. A change to 2.0 equiv of **7** and 1.0 equiv of 5-iodoquinoline resulted in full conversion with 78% yield of **8**. Scale-up reactions in multigram scale gave good 50–78% yields.

In the second step, the substituted dichloropyrimidine **8** underwent aromatic nucleophilic substitution with sodium azide in the presence of LiCl in THF, giving **9** in 88% yield. The equilibrium between azide **9a** and tetrazole **9b** is highly dependent on the polarity of the solvent.^18^ In nonpolar solvents such as benzene-*d*_6_ or CDCl_3_, only the azide form **9a** can be observed, whereas in more polar solvents such as THF-*d*_8_, DMF-*d*_7_, or DMSO-*d*_6_, both forms, **9a** and **9b,** can be observed in various ratios (see Table S1 in the SI)

In the third step, the azide **9a** can be cyclized by three different cyclization reactions: (1) thermal cyclization, (2) catalytic cyclization with different rhodium catalysts, and (3) photocyclization with UV light. The thermal cyclization of **9** in 1,4-dibromobenzene at 170 °C for 10 min gave the desired tetracyclic nucleobase **10** with 8% yield, and 59% of the starting material **9** was recovered. Prolonging the reaction time to 30 min increased the yield by only 3% and reduced the amount of recovered starting material to only 21%. Similar yields were achieved by heating the azide **9** in a microwave reactor in toluene to 170 °C for 60 min; only 10–14% of product **10** was obtained, and all starting azide **9** was consumed (see Table 1). These results suggest that the azide **9** is also decomposing at this temperature to unidentified side products.

**Table 1:** Thermal Cyclization of **9**

entry	conditions	temp (°C)	time (min)	**10**	recovered **9**
1	6 equiv 1,4-dibromobenzene	170	10	8%	59%
2	6 equiv 1,4-dibromobenzene	170	30	11%	21%
3	m.w., toluene (0.025 M)	170	60	10%	0
4	m.w., toluene (0.05 M)	170	60	14%	0

As the thermal cyclization gave only low yields and most of the starting material was just decomposed, we tried the second option: rhodium-catalyzed cyclization. We tested three different catalysts: Rh_2_esp_2_,^19^ rhodium octanoate dimer (Rh_2_(O_2_CC_7_H_15_)_4_), and rhodium heptafluorobutyrate dimer (Rh_2_(O_2_CC_3_F_7_)_4_)^20^ in toluene or in toluene/TFA (1:1) with and without molecular sieves. But none of the reaction conditions resulted in the formation of **10** (see Table S2 in the SI)

We then tried the photocyclization of **9** under our standard conditions^11,12,15^ in TFA with UV light (254 nm, 4 W) for 48 h, but it resulted only in decomposition of the azide **9**. We then tried DCM and THF as a solvent and used different photosensitizers (see Table S3 in the SI). The best result was achieved by using THF with UV light (254 nm, 4 W) and 1.0 equiv of pyrene, a singlet photosensitizer, for 72 h, which resulted in 26% of the desired nucleobase **10**. The overall yield of this three-step reaction cascade toward the quinolino-fused 7-deazapurine **10** was 18%.

**Scheme 2:**
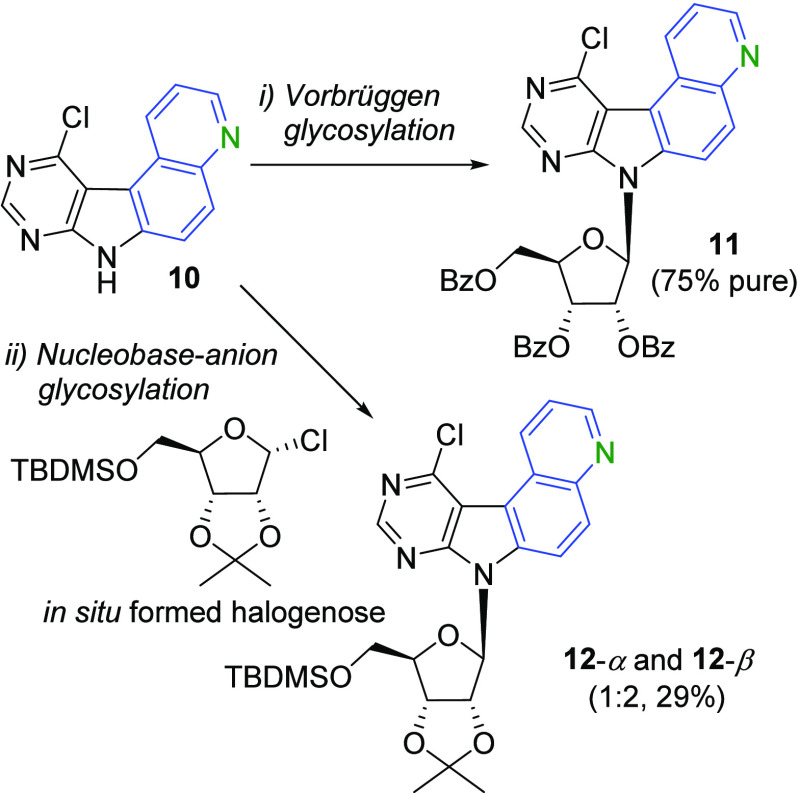
Synthesis of **12**-β and **12**-α Reagents and conditions: (i) (1) BSA, MeCN, 60 °C, 15 min, (2) 1-*O*-acetyl-2,3,5-tri-*O*-benzoyl-β-d-ribofuranose, TMSOTf, MeCN, 60 °C, 48 h; (ii) (1) KOH, TDA-1, THF, 20 °C, 30 min, then 0 °C; (2) 2,3-*O*-isopropylidene-5-*O*-*tert*-butyldimethylsilyl-d-ribofuranose, CCl_4_, HMPT, THF, −30 °C, prestirred for 1 h, then 20 °C, 36 h.

The quinolino-fused nucleobase **10** was subjected to the Vorbrüggen glycosylation,^21^ which is known to be the best option for heteroaryl-fused nucleosides.^12,14^ The nucleobase **10** was first silylated in position 7 with *N*,*O*-bis(trimethylsilyl)acetamide (BSA) and then underwent glycosylation with 1-*O*-acetyl-2,3,5-tri-*O*-benzoyl-β-d-ribofuranose in the presence of trimethylsilyl trifluoromethanesulfonate (TMSOTf), producing the key nucleoside **11** as a pure β-anomer in 52% yield (Scheme 2). An analytical sample was isolated in pure form and fully characterized. However, the purification in a larger scale was difficult, and hence, we used the crude material (ca. 75% pure) directly in the next step. The stereochemistry of **11** was confirmed by H,H-ROESY using the relations between H-6 of the nucleobase and H-2′ and H-3′ as well as between H-1′ and H-4′ of the sugar moiety.

We also tried the anion base glycosylation. First, the required halogenose was formed *in situ* from the 2,3-*O*-isopropylidene-5-*O*-*tert*-butyldimethylsilyl-d-ribofuranose^22^ with CCl_4_ and tris(dimethylamino)phosphine (HMPT). Nucleobase **10** was deprotonated by KOH and added to the halogenose together with tris[2-(2-methoxyethoxy)ethyl]amine (TDA-1). The reaction gave a mixture of **12**-β and **12**-α (2:1) with 29% overall yield (Scheme 2). The stereochemistry of the β-anomer **12**-β was also confirmed by H,H-ROESY using the same relations between H-6 of the nucleobase and H-2′ and H-3′ as well as between H-1′ and H-4′ of the sugar moiety. The α-anomer **12**-α was confirmed by using the relations between H-6 of the nucleobase, H-4′, and the methyl in the isopropylidene protecting group as well as between H-1′, H-2′, and H-3′ of the sugar moiety.

**Scheme 3:**
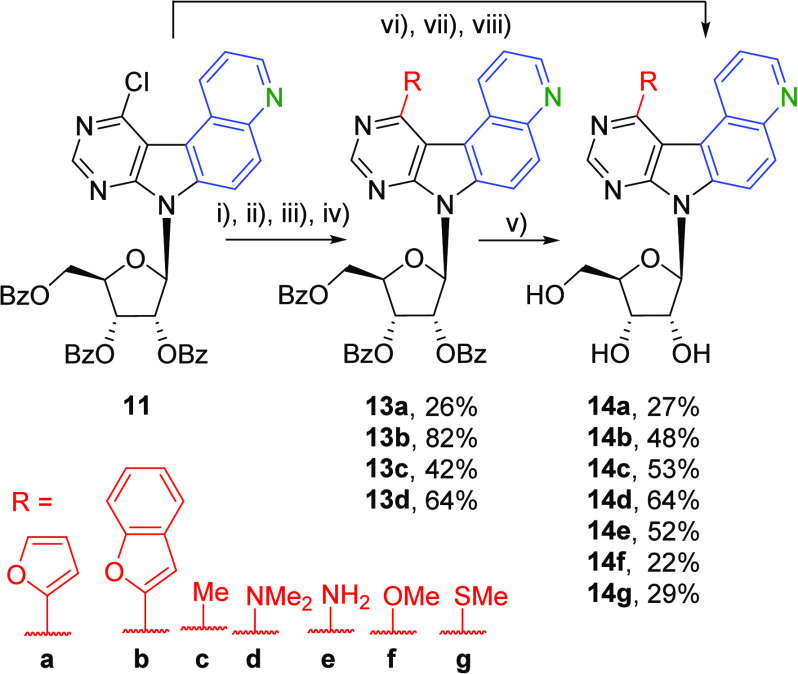
Synthesis of **14a**–**g** Reagents and conditions: (i) 2-(tributylstannyl)furan, PdCl_2_(PPh_3_)_2_, DMF, 100 °C, 24 h; (ii) benzofuran-2-ylboronic acid, K_2_CO_3_, Pd(PPh_3_)_4_, toluene, 100 °C, 24 h; (iii) AlMe_3_ (2.0 M in toluene), Pd(PPh_3_)_4_, THF, 65 °C, 3 h; (iv) Me_2_NH (2.0 M in THF), propan-2-ol, 60 °C, 24 h; (v) NaOMe (25 wt % in MeOH), MeOH, 60 °C, 18–24 h; (vi) aq. NH_3_/1,4-dioxane (5:2), 120 °C, 24 h; (vii) NaOMe (25 wt % in MeOH), MeOH, 60 °C, 4 h; (viii) NaSMe, THF, 60 °C, 18 h.

The crude (75% pure) nucleoside intermediate **11** was used in a series of reactions for derivatization in position 11 and final removal of benzoyl protecting groups from the ribose to give the desired 11-substituted quinolino-fused 7-deazapurine ribonucleosides **14a**–**g** (Scheme 3). The Stille cross-coupling of **11** with 2-(tributylstannyl)furan in the presence of PdCl_2_(PPh_3_)_2_ in DMF at 100 °C for 24 h gave the 2-furyl derivative **13a** (26%). The Suzuki–Miyaura cross-coupling of **11** with 2-benzofurylboronic acid gave the 2-benzofuryl derivative **13b** in high 82% yield. The cross-coupling of **11** with AlMe_3_ and Pd(PPh_3_)_4_ in THF at 65 °C for 3 h gave the methyl derivative **13c** with 42% yield. The nucleophilic substitution of **11** with dimethylamine in 2-propanol at 60 °C for 24 h gave the *N*,*N*-dimethylamino derivative **13d** (64%). Then, the sugar moiety of the protected nucleosides **13a**–**d** was deprotected with NaOMe in methanol at 60 °C for 18 h, resulting in the free nucleosides **14a**–**d** (27–64%). The nucleosides **14e**–**g** were obtained from **11** in a single step as the derivatization in position 11 and the deprotection of the sugar moiety happened simultaneously. Treating **11** with aqueous ammonia/1,4-dioxane (5:2) in a screw-cap pressure vial at 120 °C for 18 h resulted in the formation of the free amino derivative **14e** (52%). The reaction of **11** with NaOMe in MeOH at 60 °C for 4 h gave the free methoxy derivative **14f** in 22% yield. The reaction with NaSMe in THF at 60 °C for 18 h gave the free methylsulfanyl derivative **14g** (29%).

### Spectroscopic Properties of Quinolino-Fused 7-Deazapurine Nucleosides

Both the naphtho- and the pyrido-fused 7-deazapurine ribonucleoside derivatives^14,15^ show interesting fluorescent properties. Anisolo-fused 7-deazapurine 2′-deoxyribonucleosides have been used as nucleic acid probes.^23^ Therefore, we studied the photophysical properties of the nucleosides **14a**–**g** by measuring their absorption and emission spectra in methanol (Table 2). We then determined their molar extinction coefficient ε as well as their quantum yields Φ_f_ (see **S4** in the SI).^24^ The nucleosides **14a** and **14e** exhibited fluorescence with moderate Φ_f_ of 4.6–7.3%. Intermediate quantum yields were observed with nucleosides **14d,f,g** (Φ_f_ = 14–26%). The methyl derivative **14c** exhibited fluorescence with very good Φ_f_ of 40%. The benzofuryl derivative **14b** even exhibited an excellent fluorescence quantum yield Φ_f_ of 51%.

**Table 2:** UV and Fluorescence Maxima of Nucleosides **14a**–**g** in MeOH^a^

	absorption	emission	quantum yield
compd	max. λ_abs_ (ε) [nm (M^–1^ cm^–1^)]	max. λ_em_ [nm]	Φ_f_
**14a**	255 (27,500), *330* (5400)	436	0.073
**14b**	259 (38,200), *355* (13,600)	466	0.510
**14c**	259 (50,300), *319* (12,900), 353 (4800)	383	0.402
**14d**	256 (29,800), 298 (7700), *339* (7900), 359 (7100)	439, 610	0.136
**14e**	219 (13,600), *330* (6800)	426	0.046
**14f**	255 (38,000), 282 (16,300), *315* (9900), 346 (5700)	375, 502	0.163
**14g**	252 (36,600), *329* (10,100), 359 (6600)	390	0.255

a UV and fluorescence maxima were measured in MeOH. The used excitation wavelengths for fluorescence are in *italics*. Fluorescence quantum yields Φ_f_ 's were determined using quinine sulfate in 0.1 M H_2_SO_4_ as a standard (Φ_f_ = 0.546 at 25 °C).^24^

#### Biological Profiling

All the titled nucleosides **14a**–**g** were tested for their *in vitro* cytotoxic activity. The following cancer cell lines were used for the study: A549 (lung cancer), CCRF-CEM (acute T-lymphoblastic leukemia), HCT116 and HCT116p53^–^ (colon carcinoma, parental and p53 deficient), K562 (chronic myelogenous leukemia), and U2OS (bone osteosarcoma) using a colorimetric MTS assay.^25^ Additionally, HeLa (cervical cancer), HepG2 (hepatocellular liver carcinoma), and HL60 (acute promyelocytic leukemia) cell lines were tested using the luminescent CellTiter-Glo assay. For comparison, nonmalignant fibroblast cell lines (BJ and MRC-5) were included in the MTS assay, whereas noncancerous human dermal fibroblasts (NHDF) were assessed with the CellTiter-Glo assay.^26^ Initial screenings were done at 50 μM concentration for the MTS assay and 10 μM for the CellTiter-Glo assay. All the results are summarized in Table 3.

**Table 3:** Cytotoxic Activities of Nucleosides **14a**–**g**

	MTS assay: IC_50_ (μM)								CellTiter-Glo assay: IC_50_ (μM)			
compd	BJ	MRC-5	A549	CCRF-CEM	HCT116	HCT116p53–	K562	U2OS	NHDF	HeLa	HepG2	HL60
**14a**	>50	>50	>50	38.5	>50	>50	>50	>50	>10	>10	>10	>10
**14b**	>50	>50	>50	18.6	36.0	43.2	>50	>50	>10	>10	2.8	>10
**14c**	>50	>50	26.4	6.5	17.0	17.0	11.9	13.1	>10	>10	>10	6.7
**14d**	>50	>50	>50	>50	>50	>50	>50	>50	>10	>10	>10	>10
**14e**	>50	>50	>50	8.5	18.9	17.3	10.0	17.1	>10	>10	8.6	5.2
**14f**	>50	>50	27.4	8.0	9.8	23.1	40.2	17.3	>10	>10	>10	5.2
**14g**	36.8	36.8	17.0	5.1	9.3	16.2	18.6	15.0	>10	>10	>10	9.3

Of all the title nucleosides, the dimethylamino derivative **14d** is the only one that did not show any cytotoxicity whatsoever, which is consistent with all other heteroaryl-fused nucleosides.^11,12,14,15^ Both furyl and benzofuryl derivatives **14a** and **14b**, respectively, showed only weak activity against the CCRF-CEM leukemia line; **14b** also exhibited activity against both HCT116/HCT115p53– colon carcinoma lines and pronounced effect on the HepG2 hepatocellular carcinoma cell line, with an IC_50_ value of 2.8 μM indicating a specificity not observed in **14a**. The nucleoside **14g** bearing SMe group in position 11 displayed some moderate cytotoxic activity against a spectrum of tested cell lines including nonmalignant BJ and MRC-5, thus showing no significant selectivity toward cancerous cells. The most promising nucleosides in this series are methyl **14c**, amino **14e**, and methoxy **14f** derivatives, which all showed comparable activities against several cancer cell lines, with CCRF-CEM and HL60 being the most sensitive one with single-digit micromolar IC_50_ values and no cytotoxicity against nonmalignant cell lines BJ, MRC-5, and NHDF. Although the nucleosides **14c**, **14e**, and **14f** are more potent against the CCRF-CEM line compared to their naphtho-fused analogs (6–8 vs 20–23 μM),^15^ their activities are still 2 orders of magnitude lower than their tricyclic thieno-,^12^ furo-,^11^*N*-methylpyrrolo-,^11^ and pyrido-fused^14^ analogs. This is in agreement with our previous findings^15,16,27,28^ that nucleosides with tetracyclic nucleobases are already too bulky to be activated by phosphorylation and to interact with their biological target(s).

### Biochemistry

**Scheme 4:**
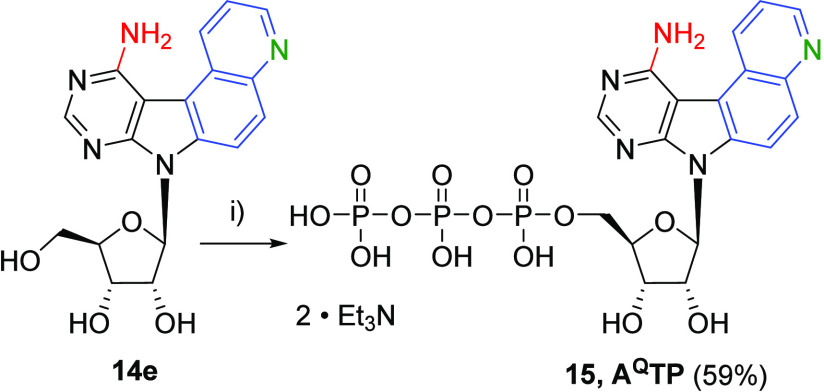
Synthesis of **15** Reagents and conditions: (i) (1) POCl_3_, PO(OMe)_3_, 0 °C, 2 h; (2) (HNBu_3_)_2_H_2_P_2_O_7_, Bu_3_N, MeCN, 0 °C, 1 h.

The amino derivative **14e** was used as an adenosine analogue to study its incorporation by *in vitro* transcription and its fluorescent properties. First, **14e** was triphosphorylated at 5′-OH according to standard procedures,^29^ resulting in the triphosphate **15** (**A**^**Q**^**TP**) with good 59% yield (Scheme 4)

**Figure 2:**
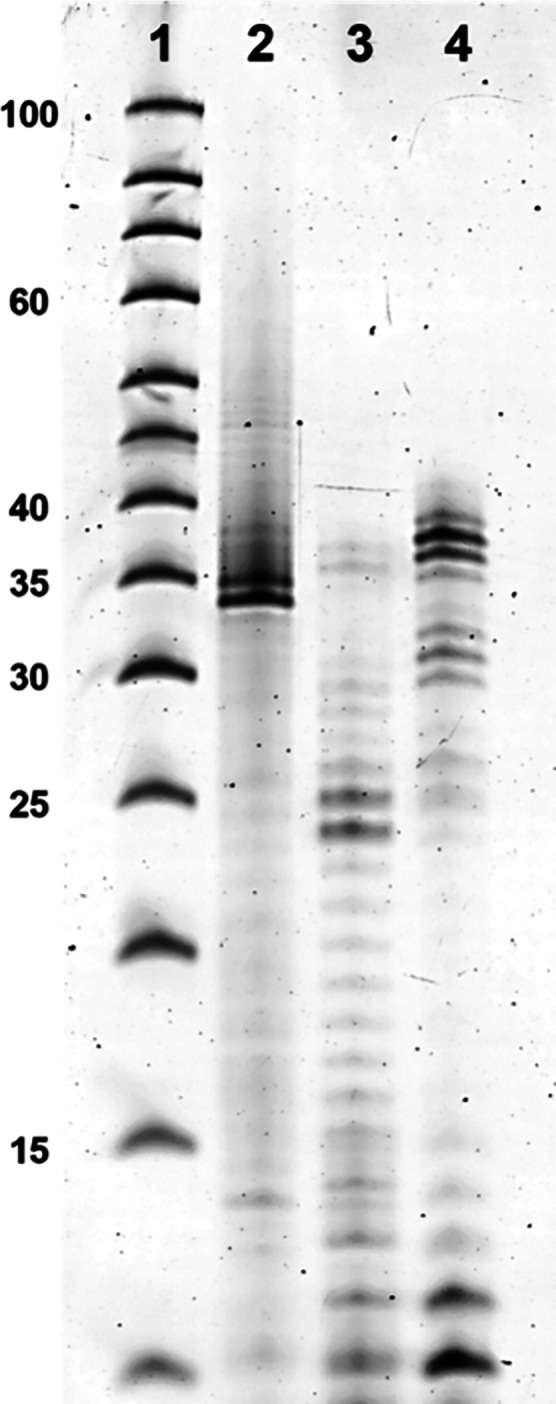
Denaturing PAGE analysis of the *in vitro* transcription reaction with T7 RNA polymerase and **35DNA_A7** template that provides seven incorporations of the modified nucleotide. Lane 1: RNA ladder, lane 2: positive control (all natural NTPs), lane 3: negative control (CTP, GTP, UTP), and lane 4: modification (**A**^**Q**^**TP**, CTP, GTP, UTP).

**A**^**Q**^**TP** was then used as a substrate for the T7 RNA polymerase in the *in vitro* transcription (IVT) experiments.^30^ We used **35DNA_A7** DNA template encoding for 35-mer RNA containing seven **A**^**Q**^ modifications (Table 4). We used the T7 High Yield RNA Synthesis Kit with a high concentration of NTPs (7.5 mM each) but without any further additives. The reaction time at 37 °C was 16 h. The positive control experiment was performed with all four natural NTPs giving nonmodified transcript **35RNA_A7** (Figure 2, lane 2). The negative control contained only the natural CTP, GTP, and UTP in the absence of ATP (Figure 2, lane 3). The real IVT experiment was performed with **A**^**Q**^**TP** and three natural NTPs (Figure 2, lane 4). The transcription products were visualized by denaturing 20% denaturing PAGE (Figure 2) and characterized by LC–MS (see Figures S1 and S2 in the SI). We observed the formation of a full-length RNA resulting in the modified RNA **35RNA_A**^**Q**^**7** containing seven **A**^**Q**^ modifications and partial formation of an *n* + 1 product containing an additional guanosine at the 3′-end of the RNA strand as a result of nontemplated addition. Unfortunately, also some truncated products were observed indicating that the incorporation of this bulky modified nucleotide by the T7 RNA polymerase was less efficient compared to standard 7-substituted 7-deaza-ATP derivatives. We also studied the absorption and emission spectra of the triphosphate **15** (**A**^**Q**^**TP**) and the oligonucleotide **35RNA_A**^**Q**^**7** in water, but the fluorescence was very weak, suggesting that this nucleotide is not the best choice for fluorescent labeling of RNA (see Table S5 in the SI).

**Table 4:** Sequences of Oligonucleotides and RNA Products

oligonucleotide	sequence	role in the study
**35DNA_A7**	5′-TAATACGACTCACTATAGGGCTTGCACGTGAATCGCTCTTAATGGATCGCGA-3′3	DNA template
	′-ATTATGCTGAGTGATATCCCGAACGTGCACTTAGCGAGAATTACCTAGCGC[mT]-5′	
**35RNA_A7**	5′-pppGGGCUUGC**A**CGUG**AA**UCGCUCUU**AA**UGG**A**UCGCG**A**	Positive control
**35RNA_A**^**Q**^**7**	5′-pppGGGCUUGC**A**^**Q**^CGUG**A**^**Q**^**A**^**Q**^UCGCUCUU**A**^**Q**^**A**^**Q**^UGG**A**^**Q**^UCGCG**A**^**Q**^	Modified RNA

## Conclusions

We developed the synthesis of the quinolino-fused 7-deazapurine **10** with 18% yield over three steps. The Negishi cross-coupling required an increased amount of the zincated pyrimidine **7** to give the coupled product **8** in a high yield of 78%, which was converted to the azide **9a** in 88% yield. The cyclization of **9** required extensive reaction screening. The thermal cyclization gave only 11% yield. The photocyclization in TFA resulted in the decomposition of **9**. The photocyclization in THF with pyrene, a singlet photosensitizer, resulted in the formation of **10** with 26% yield. We compared two glycosylation methods, and although the Vorbrüggen glycosylation gave the benzoylated nucleoside **11** as a pure β-anomer with only 75% purity (but 52% yield), it was still better than the anion base glycosylation, which gave the mixture of both anomers **12**-β and **12**-α in ratio 2:1 and only 29% total yield. The key intermediate **11** was used for final derivatization and deprotection of seven 11-substituted quinolino-fused 7-deazapurine ribonucleosides (**14a**–**g**). The fused nucleosides exerted fluorescence with moderate to excellent yields (3–51%).

Nucleosides bearing methyl **14c**, amino **14e**, and methoxy **14f** groups in position 11 on the nucleobase showed moderate cytotoxic activity against several cancer cell lines (especially CCRF-CEM with IC_50_ values of 6–8 μM) and no cytotoxicity against nonmalignant fibroblasts. This makes them more potent than their naphtho-fused analogs, suggesting a certain positive effect of a nitrogen atom in the fused ring; however, they are still not potent enough for any further development. Moreover, this series of quinolino-fused nucleosides provides another evidence that the tetracyclic nucleobases are already too bulky for interaction with their biological target, most likely for efficient intracellular phosphorylation and then incorporation into DNA and/or RNA.

The amino derivative **14e** was triphosphorylated to **15** (**A**^**Q**^**TP**) and used as an ATP analog in the *in vitro* transcription of using the T7 RNA polymerase. Unlike in case of the corresponding naphtho-fused analog,^15^**A**^**Q**^**TP** was a moderately efficient substrate for the polymerase, and we observed the formation of the corresponding full-length RNA containing seven modifications accompanied by some truncated and extended products. The moderate substrate activity and weak fluorescence do not qualify this nucleotide for a useful RNA modification and fluorescent label.

## Experimental Part

**Figure 3:**
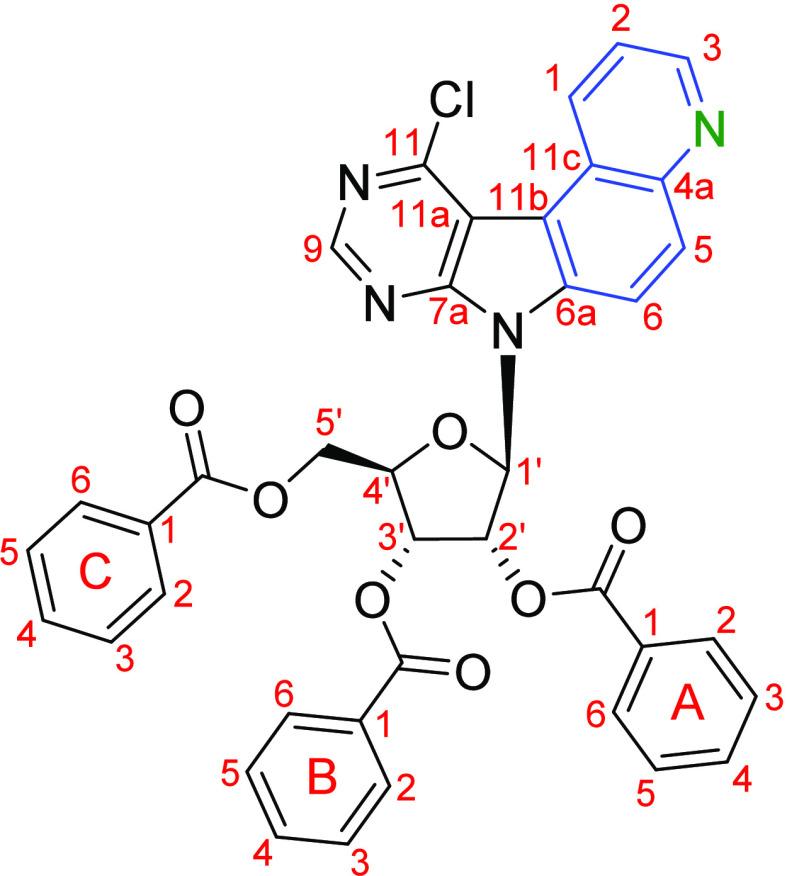
Numbering used in the assignment of NMR signals (example: key intermediate **11**).

Unless otherwise stated, all reactions were carried out under an argon atmosphere. An oil bath was used for reactions requiring heating. Thin-layer chromatography (TLC) was performed on Merck silica gel 60 F-254 aluminum sheets. Visualization was obtained by UV light (λ_max_ = 254 or 366 nm). High-performance flash chromatography (HPFC) was conducted with a Combi Flash R_f_ instrument from Teledyne Isco Inc. using SiO_2_ (particle size 0.040–0.063 mm, 230–400 mesh) from Fluorochem in refillable flash columns or HP C18 Redi Sep R_f_gold flash columns with the solvent gradient indicated in the corresponding procedures. Preparative high-pressure liquid chromatography (prep. HPLC) was performed on a Waters 2535 Quaternary Gradient System with a fraction collector. Melting points (m.p.) were measured by Bohunka Šperlichová at Charles University Prague on a Büchi Melting Point B-545 apparatus using open glass capillaries and are uncorrected. Optical rotation of final nucleosides was measured by the analytical laboratory at IOCB Prague using an AUTOPOL IV automatic polarimeter from Rudolph Research Analytical at 20 °C and 589 nm. The sample concentration *c* is given in g mL^–1^. ^1^H and ^13^C{^1^H} NMR spectra were measured on a Bruker Avance III 500 MHz spectrometer at 25 °C in CDCl_3_ referenced to the residual solvent signal (δ_H_ = 7.26 ppm, δ_C_ = 77.16 ppm), in deuterated dimethyl sulfoxide (DMSO-*d*_6_) referenced to the residual solvent signal (δ_H_ = 2.50 ppm, δ_C_ = 39.52 ppm], or in D_2_O with *t*BuOH-*d*_10_ as the external standard [δ_H_ = 1.25 ppm, δ_C_ = 31.6 ppm]. ^31^P NMR NMR spectra were referenced externally to the signal of H_3_PO_4_. Coupling constants (*J*) are given in Hz, and the multiplets are described as s (singlet), d (doublet), t (triplet), q (quartet), m (multiplet), and b (broad). ^13^C{^1^H} NMR experiments were broadband proton-decoupled and were performed using APT pulse sequence. DFQ-COSY, HSQC, and HMBC experiments were used to assign the ^1^H and ^13^C NMR signals where required. ROESY experiments were used to confirm the relative stereochemistry of nucleosides **11** and **12**. To simplify the assignment, the benzoyl group attached to the 2′-hydroxy group of the ribofuranose ring was given the letter A, the one attached to the 3′-hydroxy group is considered B, and the benzoyl group at 5′-OH is called C (Figure 3). All spectra can be found in Supporting Information S6. In the ROESY spectra for nucleosides **11**, **12**-β, and **12**-α, the important relations for the determination of the stereochemistry are highlighted. Infrared spectra (IR) were recorded on a Bruker ALPHA FT-IR spectrometer with a single-reflection Platinum ATR module. The compounds were measured in their initial state of appearance at 20 °C, and their absorption bands were reported in wavenumbers (*ṽ*) in the range between 4000 and 400 cm^–1^. Intensities are described as strong (s), medium (m), and weak (w). UV/vis spectra were measured on a Varian Cary 100 Bio UV–visible spectrophotometer in the range 250–800 nm using transparent 1.5 mL quartz cuvettes. Fluorescence spectra were recorded on a Fluoromax 4 spectrofluorimeter from HORIBA Scientific. The sample concentration was adjusted to have a UV absorbance of 0.05–0.10. The excitation was performed at the absorption maximum with the highest wavelength λ_abs_ with the slit set at 2 nm. The emission spectra were recorded from λ_abs_ + 20 nm to 2 × λ_abs_ – 20 nm with a 2 nm slit opening. High-resolution mass spectrometry (HR-MS) was measured by the MS-Service at IOCB Prague on an LTQ Orbitrap XL instrument from Thermo Fisher Scientific using electrospray ionization (ESI). The purity of the final nucleosides (>95%) was confirmed by UPLC-MS on an Agilent 1260 Infinity II LC system with an Agilent 1260 Photodiode Array Detector using a Kinetex EVO C_18_ 100 Å column (2.1 × 150 mm) from Phenomenex*.* Samples were dissolved in DMSO (1 μL injection volume). Biological activity screening was performed as described previously.^8−16,25−28^

Single-stranded DNA oligonucleotides for the preparation of the double-stranded DNA template **35DNA_A7** were purchased from Generi Biotech. The T7 Hight Yield RNA Kit, DNase I, EDTA (50 mM), and Monarch RNA purification kit (50 μg) were purchased from New England Biolabs. RNase/DNase free solutions for biochemical reactions were prepared using Milli-Q water that was treated with DEPC and sterilized by autoclaving. The precision RNA mass marker 10–100 nt was purchased in Future Synthesis. The denaturing PAGE gel was analyzed by fluorescence (λ_ex_ = 532 nm) using a Typhoon FLA 9500 from GE Healthcare Life Sciences. LC-ESI-MS analysis of oligonucleotides was carried out on an Agilent 1260 Infinity II LC system with an Agilent InfinityLab LS/MSD XT Detector using a BioZen C_18_ 100 Å column (2.1 × 150 mm) from Phenomenex with the mobile phases A (12.2 mM Et_3_N, 300 mM HFIP in water) and B (12.2 mM Et_3_N, 300 mM HFIP in 100% MeOH) and a gradient from 95:5 to 0:100 within 10 min. Deconvolutions of the LC-ESI-MS spectra were carried out using a UniDec program.

### 5-(4,6-Dichloropyrimidin-5-yl)quinoline (**8**)

Dry ZnCl_2_ (2.44 g, 17.89 mmol) was treated with a solution of (TMP)MgCl·LiCl (35.7 mL, 1.0 M solution in THF/toluene, 35.7 mmol) and stirred at 20 °C for 24 h. After cooling to 0 °C, a solution of 4,6-dichloropyrimidine (4.43 g, 29.73 mmol) in THF (5.0 mL) was slowly added. The mixture was stirred for 2 h and treated with a solution of 5-iodoquinoline (3.79 g, 14.86 mmol) and Pd(PPh_3_)_4_ (1.72 g, 1.49 mmol) in THF (20.0 mL). The mixture was stirred at 65 °C for 24 h, treated with water (2 mL), and evaporated. HPFC (SiO_2_; cHex/EtOAc, gradient 0 → 25% EtOAc) gave **8** (3.19 g, 78%) as a pale-yellow solid. *R*_f_ = 0.45 (SiO_2_; cHex/EtOAc 2:1); mp = 179 °C; ^1^H NMR (500 MHz, CDCl_3_): δ = 7.83 (dd, *J*_*6,7*_ = 7.2 Hz, *J*_*6,8*_ = 1.0 Hz, 1 H; H-6), 7.91 (dd, *J*_*3,4*_ = 8.6 Hz, *J*_*3,2*_ = 5.1 Hz, 1 H; H-3), 8.20 (dd, *J*_*7,8*_ = 8.7 Hz, *J*_*7,6*_ = 7.2 Hz, 1 H; H-7), 8.30 (d, *J*_*4,3*_ = 8.5 Hz, 1 H; H-4), 9.01 (s, 1 H; H-2′), 9.09 (d, *J*_*8,7*_ = 8.7 Hz, 1 H; H-8), 9.15 ppm (dd, *J*_*2,3*_ = 5.1 Hz, *J*_*2,4*_ = 1.5 Hz, 1 H; H-2); ^13^C{^1^H} NMR (125.7 MHz, CDCl_3_): δ = 122.30 (C-3), 124.89 (C-8), 126.73 (C-4a), 129.31 (C-5′), 131.68 (C-5), 132.00 (C-6), 134.39 (C-7), 139.65 (C-8a), 141.50 (C-4), 144.21 (C-2), 158.86 (C-2′), 162.41 ppm (2 C; C-4′, C-6′); IR (ATR, neat): *ṽ* = 3053 (w), 2991 (w), 2750–1700 (br w), 1601 (w), 1571 (w), 1548 (w), 1511 (m), 1500 (s), 1403 (s), 1377 (m), 1351 (m), 1311 (w), 1225 (m), 1162 (w), 1095 (w), 1036 (w), 1022 (w), 977 (w), 954 (m), 846 (w), 829 (w), 802 (s), 784 (s), 743 (m), 663 (m), 642 (w), 589 (w), 572 (w), 540 (w), 510 (w), 473 (w), 459 (w), 441 (w), 429 cm^–1^ (w); HR MS (ESI) for C_13_H_8_N_3_^35^Cl_2_^+^ [M(^35^Cl) + H]^+^: calcd 276.00953, found 276.00892; for C_13_H_8_N_3_^35^Cl^37^Cl^+^ [M(^35^Cl^37^Cl) + H]^+^: calcd 278.006578, found 278.00582; for C_13_H_8_N_3_^37^Cl_2_^+^ [M(^37^Cl) + H]^+^: calcd 280.00363, found 280.00275.

### 5-(4-Azido-6-chloropyrimidin-5-yl)quinoline (**9a**)/5-(7-Chlorotetrazolo[1,5-*c*]pyrimidin-8-yl)quinoline (**9b**)

Compound **8** (4.07 g, 14.73 mmol), sodium azide (957.4 mg, 14.73 mmol), and lithium chloride (628.2 mg, 14.82 mmol) were dissolved in THF (60 mL) and stirred in the dark at 20 °C for 24 h. The mixture was treated with water (1 mL) and concentrated. HPFC (SiO_2_; cHex/EtOAc, gradient 0 → 50% EtOAc) gave **9** (3.6496 g, 88%) as an off-white solid. *R*_f_ = 0.64 (SiO_2_; cHex/EtOAc 1:1); mp = 154–158 °C (decomp.); ^1^H NMR of **9a** (500 MHz, CDCl_3_): δ = 7.43 (dd, *J*_*3,4*_ = 8.5 Hz, *J*_*3,2*_ = 4.2 Hz, 1 H; H-3), 7.45 (dd, *J*_*6,7*_ = 7.1 Hz, *J*_*6,8*_ = 1.0 Hz, 1 H; H-6), 7.70 (bd, *J*_*4,3*_ = 8.5 Hz, 1 H; H-4), 7.83 (dd, *J*_*7,8*_ = 8.5 Hz, *J*_*7,6*_ = 7.1 Hz, 1 H; H-7), 8.28 (bd, *J*_*8,7*_ = 8.5 Hz, 1 H; H-8), 8.82 (s, 1 H; H-2’), 8.99 ppm (dd, *J*_*2,3*_ = 4.2 Hz, *J*_*2,4*_ = 1.6 Hz, 1 H; H-2); ^13^C NMR of **9a** (125.7 MHz, CDCl_3_): δ = 120.86 (C-5′), 121.97 (C-3), 126.24 (C-4a), 128.65 (C-6), 129.30 (C-7), 129.39 (C-5), 131.15 (C-8), 133.15 (C-4), 148.02 (C-8a), 150. 70 (C-2’), 147.59 (C-2), 157.83 (C-2’), 161.82 and 163.04 ppm (2 C; C-4′, C-6’); **9b** was not observed in CDCl_3_; ^1^H NMR of **9a** (500 MHz, DMSO-*d*_6_): δ = 7.53 (dd, *J*_*3,4*_ = 8.5 Hz, *J*_*3,2*_ = 4.2 Hz, 1 H; H-3), 7.62 (dd, *J*_*6,7*_ = 7.1 Hz, *J*_*6,8*_ = 1.2 Hz, 1 H; H-6), 7.88 (dd, *J*_*7,8*_ = 8.5 Hz, *J*_*7,6*_ = 7.1 Hz, 1 H; H-7), 7.98 (ddd, *J*_*4,3*_ = 8.5 Hz, *J*_*4,2*_ = 1.6 Hz, *J*_*4,8*_ = 0.9 Hz, 1 H; H-4), 8.15 (dt, *J*_*8,7*_ = 8.5 Hz, *J*_*8,6*_ = *J*_*8,4*_ = 1.0 Hz, 1 H; H-8), 8.97 (dd, *J*_*2,3*_ = 4.2 Hz, *J*_*2,4*_ = 1.7 Hz, 1 H; H-2), 8.99 ppm (s, 1 H; H-2’); ^13^C NMR of **9a** (125.7 MHz, DMSO-*d*_6_): δ = 120.46 (C-5′), 122.22 (C-3), 125.84 (C-4a), 128.59 (C-6), 129.24 (C-7), 129.59 (C-5), 130.34 (C-8), 133.35 (C-4), 147.53 (C-8a), 150.89 (C-2), 157.86 (C-2’), 160.43 and 162.35 ppm (2 C; C-4′, C-6’); ^1^H NMR of **9b** (500 MHz, DMSO-*d*_6_): δ = 7.52 (dd, *J*_*3,4*_ = 8.5 Hz, *J*_*3,2*_ = 4.1 Hz, 1 H; H-3), 7.77 (dd, *J*_*6,7*_ = 7.1 Hz, *J*_*6,8*_ = 1.2 Hz, 1 H; H-6), 7.99 (dd, *J*_*7,8*_ = 8.5 Hz, *J*_*7,6*_ = 7.1 Hz, 1 H; H-7), 8.08 (ddd, *J*_*4,3*_ = 8.5 Hz, *J*_*4,2*_ = 1.6 Hz, *J*_*4,8*_ = 1.0 Hz, 1 H; H-4), 8.27 (dt, *J*_*8,7*_ = 8.5 Hz, *J*_*8,6*_ = *J*_*8,4*_ = 1.1 Hz, 1 H; H-8), 9.00 (dd, *J*_*2,3*_ = 4.2 Hz, *J*_*2,4*_ = 1.7 Hz, 1 H; H-2), 10.41 ppm (s, 1 H; H-5′); ^13^C NMR of **9b** (125.7 MHz, DMSO-*d*_6_): δ = 120.00 (C-8’), 122.11 (C-3), 125.95 (C-4a), 128.57 (C-5), 128.82 (C-6), 129.33 (C-7), 131.07 (C-8), 133.72 (C-4), 139.91 (C-5′), 147.29 (C-9’), 147.59 (C-8a), 151.13 (C-2), 151.58 ppm (C-7’); IR (ATR, neat): *ṽ* = 2294 (w), 2203 (w), 2137 (s), 2041 (w), 1599 (w), 1571 (w), 1550 (w), 1524 (s), 1499 (m), 1425 (w), 1402 (s), 1358 (s), 1316 (m), 1305 (s), 1225 (w), 1200 (w), 1178 (w), 1145 (s), 1078 (w), 1058 (w), 1023 (w), 978 (w), 954 (m), 919 (w), 896 (s), 847 (w), 830 (w), 802 (s), 793 (s), 771 (s), 751 (m), 736 (m), 694 (w), 663 (w), 637 (m), 586 (m), 539 (m), 510 (m), 492 (w), 459 (w), 437 cm^–1^ (m); HR MS (ESI) for C_13_H_8_N_6_^35^Cl^+^ [M(^35^Cl) + H]^+^: calcd 283.04990, found 283.04945; for C_13_H_8_N_6_^37^Cl^+^ [M(^37^Cl) + H]^+^: calcd 285.04695, found 285.04643.

### 11-Chloro-7*H*-pyrimido[5′,4′:4,5]pyrrolo[3,2-*f*]quinoline (**10**)

Two batches were prepared at the same time. For each batch, a solution of **10** (300.0 mg, 1.06 mmol) and pyrene (214.6 mg, 1.06 mmol) in THF (42.4 mL) was irradiated under UV light (254 nm, 4 W) at r.t. under ambient atmosphere. Every 12 h, the reaction mixtures were sonicated for 30 s to remove any precipitation from the light source. After 72 h, the batches were combined, concentrated *in vacuo,* and purified by HPFC (SiO_2_; DCM/EtOAc, gradient 0 → 100% EtOAc), giving **10** (137.6 mg, 26%) as a light-brown solid. *R*_f_ = 0.11 (SiO_2_; cHex/EtOAc 1:1); mp = 170–185 °C (decomp.); ^1^H NMR (500 MHz, DMSO-*d*_6_): δ = 7.69 (dd, 1 H, *J*_*2,1*_ = 8.7 Hz, *J*_*2,3*_ = 4.2 Hz; H-2), 8.00 (bd, 1 H, *J*_*6,5*_ = 9.0 Hz; H-6), 8.19 (bd, 1 H, *J*_*5,6*_ = 9.0 Hz; H-5), 8.80 (s, 1 H; H-9), 8.90 (dd, 1 H, *J*_*3,2*_ = 4.2 Hz, *J*_*3,1*_ = 1.5 Hz; H-3), 9.83 (bd, 1 H, *J*_*1,2*_ = 8.7 Hz; H-1), 13.43 ppm (s, 1 H; N*H*); ^13^C NMR (125.7 MHz, DMSO-*d*_6_): δ = 110.43 (C-11b), 112.79 (C-11a), 116.10 (C-6), 121.56 (C-2), 123.70 (C-11c), 131.58 (C-5), 133.51 (C-1), 136.95 (C-6a), 145.22 (C-4a), 147.59 (C-3), 150.54 (C-11), 152.39 (C-9), 155.33 ppm (C-4a); IR (ATR, neat): *ṽ* = 3500–2250 (br w), 1727 (m), 1684 (m), 1590 (m), 1562 (m), 1542 (m), 1503 (m), 1465 (m), 1439 (m), 1412 (m), 1385 (m), 1367 (m), 1306 (m), 1234 (s), 1188 (m), 1161 (m), 1094 (m), 1055 (m), 1016 (m), 978 (m), 947 (s), 845 (m), 806 (s), 785 (s), 767 (s), 693 (m), 663 (s), 631 (s), 586 (s), 542 (s), 509 (s), 468 (m), 435 (s), 425 cm^–1^ (m); HR MS (ESI) for C_13_H_8_N_4_^35^Cl^+^ [M(^35^Cl) + H]^+^: calcd 255.04375, found 255.04323; for C_13_H_8_N_4_^37^Cl^+^ [M(^37^Cl) + H]^+^: calcd 257.04080, found 257.04018.

### 11-Chloro-7-(2,3,5-tri-*O*-benzoyl-β-d-ribofuranosyl)pyrimido[5′,4′:4,5]pyrrolo[3,2-*f*]quinoline (**11**)

A suspension of nucleobase **10** (220.9 mg, 0.87 mmol) in MeCN (26.5 mL) was treated with BSA (0.32 mL, 1.31 mmol) and stirred at 60 °C for 15 min. 1-*O*-Acetyl-2,3,5-tri-*O*-benzoyl-β-d-ribofuranose (875.6 mg, 1.74 mmol; dried under a vacuum at 60 °C for 6 h) was added under argon flow followed by TMSOTf (0.32 mL, 1.74 mmol). The mixture was stirred at 60 °C for 48 h, treated with water (25 mL), concentrated *in vacuo*, and extracted with EtOAc (2 × 25 mL). The combined organic layers were washed with saturated NaHCO_3_ (50 mL) and water (50 mL), dried over MgSO_4_, and concentrated *in vacuo*. Purification by HPFC (SiO_2_; cHex/EtOAc, gradient 0 → 60% EtOAc) gave impure **11** (419.1 mg, 75% pure, 52%) as a light gray foam. A small amount was repurified by preparative TLC (C_18_; pure MeCN), resulting in a white foam prior to characterization. *R*_f_ = 0.40 (SiO_2_; cHex/EtOAc 1:1); ^1^H NMR (500 MHz, CDCl_3_): δ = 4.76 (dd, *J*_*gem*_ = 12.3 Hz, *J*_*5′a,4’*_ = 3.9 Hz, 1 H; H-5′a), 4.87 (dt, *J*_*4′,3′*_ = 6.1 Hz, *J*_*4′,5′a*_ = *J*_*4′,5′b*_ = 3.4 Hz, 1 H; H-4’), 4.99 (dd, *J*_*gem*_ = 12.3 Hz, *J*_*5′b,4’*_ = 2.9 Hz, 1 H; H-5′b), 6.44 (t, *J*_*3′,2’*_ = *J*_*3′,4’*_ = 6.3 Hz, 1 H; H-3′), 6.67 (dd, *J*_*2′,3′*_ = 6.5 Hz, *J*_*2′,1’*_ = 5.1 Hz, 1 H; H-2’), 7.02 (d, *J*_*1′,2’*_ = 5.1 Hz, 1 H; H-1’), 7.34, 7.42, and 7.45 (3 × m, 3 × 2 H; H-A3, H-A5, H–B3, H–B5, H–C3, H–C5), 7.53, 7.59, and 7.61 (3 × m, 3 H; H-A4, H–B4, H–C4), 7.61 (dd, *J*_*2,1*_ = 8.9 Hz, *J*_*2,3*_ = 4.1 Hz, 1 H; H-2), 7.88, 8.03, and 8.07 (3 × m, 3 × 2 H; H-A2, H-A6, H–B2, H–B6, H–C2, H–C6), 8.12 (d, *J*_*5,6*_ = 9.3 Hz, 1 H; H-5), 8.17 (d, *J*_*6,5*_ = 9.3 Hz, 1 H; H-6), 8.77 (s, 1 H; H-9), 8.97 (dd, *J*_*3,2*_ = 4.1 Hz, *J*_*3,1*_ = 1.3 Hz, 1 H; H-3), 10.00 ppm (d, *J*_*1,2*_ = 8.9 Hz, 1 H; H-1); ^13^C{^1^H} NMR (125.7 MHz, CDCl_3_): δ = 63.35 (C-5′), 70.89 (C-3′), 73.03 (C-2’), 80.23 (C-4’), 87.01 (C-1’), 113.13 (C-11b), 114.65 (C-6), 114.83 (C-11a), 121.63 (C-2), 124.53 (C-11c), 128.64 (C-A1), 128.66, 128.71, and 128.77 (3 × 2 C; C-A3, C-A5, C–B3, C–B5, C–C3, C–C5), 128.92 and 129.49 (2 C; C–B1, C–C1), 129.85, 129.93, and 130.00 (3 × 2 C; C-A2, C-A6, C–B2, C–B6, C–C2, C–C6), 132.72 (C-5), 133.71, 133.87, and 133.90 (3 C; C-A4, C–B4, C–C4), 134.95 (C-1), 136.77 (C-6a), 146.15 (C-4a), 148.75 (C-3), 152.28 (C-11), 152.40 (C-9), 155.40 (C-7a), 165.37, 165.60, and 166.27 ppm (3 C; C = O); IR (ATR, neat): *ṽ* = 2921 (w), 2851 (w), 1721 (m), 1601 (w), 1585 (w), 1560 (w), 1533 (w), 1518 (w), 1467 (w), 1450 (w), 1406 (w), 1375 (w), 1314 (w), 1262 (s), 1243 (m), 1176 (w), 1157 (w), 1090 (m), 1068 (m), 1024 (m), 945 (w), 847 (w), 810 (m), 787 (m), 768 (w), 707 (s), 686 (m), 617 (w), 546 (w), 509 (w), 461 (w), 436 (w), 421 cm^–1^ (w); HR MS (ESI) for C_39_H_28_N_4_O_7_^35^Cl^+^ [M(^35^Cl) + H]^+^: calcd 699.16465, found 699.16342; for C_39_H_28_N_4_O_7_^37^Cl^+^ [M(^37^Cl) + H]^+^: calcd 701.16170, found 701.16101.

### 11-Chloro-7-(2,3-*O*-isopropylidene-5-*O*-*tert*-butyldimethylsilyl-β-d-ribofuranosyl)pyrimido[5′,4′:4,5]pyrrolo[3,2-*f*]quinoline (**12-β**)/11-Chloro-7-(2,3-*O*-isopropylidene-5-*O*-*tert*-butyldimethylsilyl-α-d-ribofuranosyl)pyrimido[5′,4′:4,5]pyrrolo[3,2-*f*]quinoline (**12-α**)

A solution of previously prepared 2,3-*O*-isopropylidene-5-*O*-*tert*-butyldimethylsilyl-d-ribofuranose^22^ (179.6 mg, 0.59 mmol) in THF (2.9 mL) was cooled to −30 °C and treated first with CCl_4_ (77 μL, 0.79 mmol) and then dropwise with HMPT (141 μL, 0.79 mmol). The desired halosugar was formed during 1 h. Nucleobase **10** (100.3 mg, 0.40 mmol) and powdered KOH (44.5 mg, 0.79 mmol) in MeCN (2.5 mL) were treated with TDA-1 (0.13 mL, 0.40 mmol) and stirred for 30 min. The halosugar solution was added via a syringe at 0 °C. The mixture was first stirred at 0 °C for 30 min and then at r.t. for 36 h. The mixture was treated with water (0.25 mL) and concentrated. HPFC (SiO_2_; cHex/EtOAc, gradient 0 → 25% EtOAc) gave the pure β-anomer **12-β** (14.4 mg, 7%), a light beige solid, which was used for characterization, together with a mixed fraction containing an anomeric mixture **12-β/-α** 0.8:0.2 (32.4 mg, 15%) as a light-yellow solid and **12-α** (16.4 mg, 8%) as a sticky yellow solid. Additionally, some starting material **10** (16.0 mg, 16%) was recovered.

#### 12-β

*R*_f_ = 0.55 (SiO_2_; cHex/EtOAc 2:1); ^1^H NMR (500 MHz, CDCl_3_): δ = 0.11 and 0.12 (2 × s, 2 × 3 H; Si*Me*_2_), 0.95 (s, 9 H; C*Me*_3_), 1.39 and 1.70 (2 × s, 2 × 3 H; OC*Me*_2_), 3.91 (dd, *J*_*gem*_ = 11.4 Hz, *J*_*5′a,4’*_ = 3.7 Hz, 1 H; H-5′a), 4.01 (dd, *J*_*gem*_ = 11.4 Hz, *J*_*5′b,4’*_ = 3.3 Hz, 1 H; H-5′b), 4.31 (q, *J*_*4′,3′*_ = *J*_*4′,5′a*_ = *J*_*4′,5′b*_ = 3.7 Hz, 1 H; H-4’), 5.21 (dd, *J*_*3′,2’*_ = 6.9 Hz, *J*_*3′,4’*_ = 4.3 Hz, 1 H; H-3′), 5.41 (dd, *J*_*2′,3′*_ = 6.9 Hz, *J*_*2′,1’*_ = 4.0 Hz, 1 H; H-2’), 6.92 (d, *J*_*1′,2’*_ = 4.0 Hz, 1 H; H-1’), 7.62 (dd, *J*_*2,1*_ = 8.7 Hz, *J*_*2,3*_ = 4.2 Hz, 1 H; H-2), 8.28 (d, *J*_*6,5*_ = 9.2 Hz, 1 H; H-6), 8.32 (d, *J*_*5,6*_ = 9.2 Hz, 1 H; H-5), 8.84 (s, 1 H; H-9), 8.98 (dd, *J*_*3,2*_ = 4.2 Hz, *J*_*3,1*_ = 1.6 Hz, 1 H; H-3), 10.02 ppm (dd, *J*_*1,2*_ = 8.7 Hz, *J*_*1,2*_ = 1.6 Hz, 1 H; H-1); ^13^C{^1^H} NMR (125.7 MHz, CDCl_3_): δ = −5.25 and −5.10 (2 C; Si*Me*_2_), 18.71 (Si*C*Me_3_), 25.68 and 27.56 (2 C; OC*Me*_2_), 26.17 (Si*C*Me_3_), 62.69 (C-5′), 80.00 (C-3′), 82.87 (C-2’), 85.32 (C-4’), 88.82 (C-1’), 113.07 (C-11b), 114.38 (C-11a), 115.54 (O*C*Me_2_), 115.99 (C-6), 121.56 (C-2), 124.47 (C-11c), 132.52 (C-5), 134.91 (C-1), 136.58 (C-6a), 146.14 (C-4a), 148.65 (C-3), 152.08 (C-11), 152.43 (C-9), 155.32 ppm (C-7a); IR (ATR, neat): *ṽ* = 2929 (w), 2856 (w), 1737 (very w), 1610 (w), 1588 (w), 1559 (w), 1531 (w), 1518 (m), 1468 (m), 1440 (w), 1427 (w), 1382 (w), 1372 (w), 1316 (w), 1242 (m), 1211 (m), 1136 (m), 1080 (s), 1007 (m), 974 (w), 946 (w), 929 (m), 888 (w), 832 (s), 810 (s), 777 (s), 670 (w), 627 (w), 567 (w), 546 (w), 512 (w), 469 (w), 432 cm^–1^ (w); HR MS (ESI) for C_27_H_34_N_4_O_4_^35^ClSi^+^ [M(^35^Cl) + H]^+^: calcd 541.20379, found 541.20359; for C_27_H_34_N_4_O_4_^37^ClSi^+^ [M(^37^Cl) + H]^+^: calcd 543.20084, found 543.20082; C_27_H_33_N_4_O_4_^35^ClSiNa^+^ [M(^35^Cl) + Na]^+^: calcd 563.18573, found 563.18567; for C_27_H_33_N_4_O_4_^37^ClSiNa^+^ [M(^37^Cl) + Na]^+^: calcd 565.18278, found 565.18286.

#### 12-α

*R*_f_ = 0.44 (SiO_2_; cHex/EtOAc 2:1); ^1^H NMR (500 MHz, CDCl_3_): δ = 0.16 and 0.19 (2 × s, 2 × 3 H; Si*Me*_2_), 1.03 (s, 9 H; C*Me*_3_), 1.27 and 1.34 (2 × s, 2 × 3 H; OC*Me*_2_), 3.91 (dd, *J*_*gem*_ = 10.9 Hz, *J*_*5′a,4’*_ = 1.8 Hz, 1 H; H-5′a), 4.01 (dd, *J*_*gem*_ = 10.9 Hz, *J*_*5′b,4’*_ = 2.4 Hz, 1 H; H-5′b), 4.66 (t, *J*_*4′,5′a*_ = *J*_*4′,5′b*_ = 2.2 Hz, 1 H; H-4’), 5.04–5.08 (m, 2 H; H-2′, H-3′), 7.45 (m, 1 H; H-1’), 7.57 (dd, *J*_*2,1*_ = 8.7 Hz, *J*_*2,3*_ = 4.2 Hz, 1 H; H-2), 8.24 (d, *J*_*5,6*_ = 9.2 Hz, 1 H; H-5), 8.57 (d, *J*_*6,5*_ = 9.2 Hz, 1 H; H-6), 8.76 (s, 1 H; H-9), 8.93 (dd, *J*_*3,2*_ = 4.2 Hz, *J*_*3,1*_ = 1.6 Hz, 1 H; H-3), 10.00 ppm (dm, *J*_*1,2*_ = 8.7 Hz, 1 H; H-1); ^13^C{^1^H} NMR (125.7 MHz, CDCl_3_): δ = −5.33 and −5.34 (2 C; Si*Me*_2_), 18.35 (Si*C*Me_3_), 23.51 and 25.38 (2 C; OC*Me*_2_), 26.08 (Si*C*Me_3_), 66.24 (C-5′), 80.79 (C-3′), 82.49 (C-2’), 83.07 (C-4’), 88.78 (C-1’), 112.55 (C-11b), 113.47 (O*C*Me_2_), 113.95 (C-11a), 120.07 (C-6), 121.08 (C-2), 124.25 (C-11c), 130.88 (C-5), 134.88 (C-1), 38.37 (C-6a), 146.02 (C-4a), 148.15 (C-3), 151.60 (C-11), 152.12 (C-9), 154.86 ppm (C-7a); IR (ATR, neat): *ṽ* = 2929 (w), 2856 (w), 1610 (w), 1588 (w), 1558 (w), 1532 (m), 1515 (m), 1469 (m), 1438 (w), 1383 (w), 1375 (w), 1337 (w), 1316 (w), 1272 (w), 1241 (s), 1206 (m), 1161 (m), 1124 (m), 1076 (s), 1053 (m), 1026 (w), 989 (m), 973 (m), 939 (m), 912 (m), 881 (w), 833 (s), 810 (s), 777 (s), 728 (s), 674 (w), 640 (w), 608 (w), 578 (w), 544 (w), 515 (w), 472 (w), 440 cm^–1^ (w); HR MS (ESI) for C_27_H_34_N_4_O_4_^35^ClSi^+^ [M(^35^Cl) + H]^+^: calcd 541.20379, found 541.20366; for C_27_H_34_N_4_O_4_^37^ClSi^+^ [M(^37^Cl) + H]^+^: calcd 543.20084, found 543.20080; C_27_H_33_N_4_O_4_^35^ClSiNa^+^ [M(^35^Cl) + Na]^+^: calcd 563.18573, found 563.18558; for C_27_H_33_N_4_O_4_^37^ClSiNa^+^ [M(^37^Cl) + Na]^+^: calcd 565.18278, found 565.18288.

### 11-(Furan-2-yl)-7-(2,3,5-tri-*O*-benzoyl-β-d-ribofuranosyl)pyrimido[5′,4′:4,5]pyrrolo[3,2-*f*]quinoline (**13a**)

A solution of the crude nucleoside **11** (427.1 mg, 75%, 0.46 mmol) in DMF (4.6 mL) was treated with PdCl_2_(PPh_3_)_2_ (32.4 mg, 0.05 mmol) and 2-(tributylstannyl)furan (0.18 mL, 0.55 mmol) and stirred at 100 °C for 24 h. Purification by HPFC (SiO_2_; cHex/EtOAc, gradient 0 → 25% EtOAc) gave **13a** (85.3 mg, 26%) as a yellow film. *R*_f_ = 0.39 (SiO_2_; cHex/EtOAc 1:1); ^1^H NMR (500 MHz, CDCl_3_): δ = 4.78 (dd, *J*_*gem*_ = 12.2 Hz, *J*_*5′a,4’*_ = 4.2 Hz, 1 H; H-5′a), 4.89 (ddd, *J*_*4′,3′*_ = 6.3 Hz, *J*_*4′,5′a*_ = 4.1 Hz, *J*_*4′,5′b*_ = 3.1 Hz, 1 H; H-4’), 4.99 (dd, *J*_*gem*_ = 12.2 Hz, *J*_*5′b,4’*_ = 3.0 Hz, 1 H; H-5′b), 6.48 (t, *J*_*3′,2’*_ = *J*_*3′,4’*_ = 6.3 Hz, 1 H; H-3′), 6.71 (bdd, *J*_*2′,3′*_ = 6.5 Hz, *J*_*2′,1’*_ = 4.9 Hz, 1 H; H-2’), 6.75 (dd, *J*_*4,3*_ = 3.5 Hz, *J*_*4,5*_ = 1.8 Hz, 1 H; H-4-furyl), 6.99 (d, *J*_*1′,2’*_ = 4.9 Hz, 1 H; H-1’), 7.25 (d, *J*_*3,4*_ = 3.5 Hz, H-3-furyl), 7.34 (m, 2 H; H-A3, H-A5), 7.39 (m, 1 H; H-6), 7.42 and 7.45 (2 × m, 2 × 2 H; H–B3, H–B5, H–C3, H–C5), 7.53 (m, 1 H; H-A4), 7.56–7.63 (m, 3 H, H-5-furyl, H–B4, H–C4), 7.85 (m, 1 H; H-1), 7.90, 8.04, and 8.08 (3 × m, 3 × 2 H; H-A2, H-A6, H–B2, H–B6, H–C2, H–C6), 8.23 (d, *J*_*6,5*_ = 9.2 Hz, H-6), 8.35 (bm, 1 H; H-5), 8.87 (dd, *J*_*3,2*_ = 4.5 Hz, *J*_*3,1*_ = 1.6 Hz, 1 H; H-3), 9.01 ppm (s, 1 H; H-9); ^13^C{^1^H} NMR (125.7 MHz, CDCl_3_): δ = 63.51 (C-5′), 71.03 (C-3′), 73.24 (C-2’), 80.24 (C-4’), 87.03 (C-1’), 111.57 (C-11a), 113.30 (C-4-furyl), 114.01 (C-3-furyl), 114.11 (C-11b), 116.10 (C-6), 120.84 (C-2), 124.78 (C-11c), 128.7 (C-A1), 128.67, 128.71, and 128.77 (6 C; C-A3, C-A5, C–B3, C–B5, C–C3, C–C5), 128.94 and 129.52 (2 C; C–B1, C–C1), 129.86, 129.94, and 130.00 (6 C; C-A2, C-A6, C–B2, C–B6, C–C2, C–C6), 133.69, 133.86, and 133.91 (3 C; C-A4, C–B4, C–C4), 137.44 (C-6a), 145.18 and 145.20 (2 C; C-3, C-5-furyl), 150.00 (C-11), 151.97 (C-2-furyl), 153.19 (C-9), 155.76 (C-7a), 165.47, 165.61, and 166.32 ppm (3 C; C = O), 3 C (C-1, C-4a, C-5) not detected; IR (ATR, neat): *ṽ* = 3065 (w), 2919 (w), 2852 (w), 1724 (s), 1601 (w), 1584 (w), 1562 (m), 1538 (w), 1518 (m), 1491 (w), 1464 (w), 1451 (m), 1375 (w), 1315 (w), 1264 (s), 1177 (w), 1161 (w), 1116 (s), 1093 (s), 1069 (m), 1025 (m), 932 (w), 885 (w), 850 (w), 813 (w), 798 (w), 751 (s), 708 (m), 687 (w), 633 (w), 617 (w), 595 (w), 548 (w), 463 (w), 444 (w), 416 cm^–1^ (w); HR MS (ESI) for C_43_H_31_N_4_O_8_^+^ [M + H]^+^: calcd 731.21419, found 731.21329; for C_43_H_30_N_4_O_8_Na^+^ [M + Na]^+^: calcd 753.19613, found 753.19531.

### 11-(Benzofuran-2-yl)-7-(2,3,5-tri-*O*-benzoyl-β-d-ribofuranosyl)pyrimido[5′,4′:4,5]pyrrolo[3,2-*f*]quinoline (**13b**)

A solution of the impure nucleoside **11** (300.2 mg, 75%, 0.32 mmol) in toluene (4.3 mL) was treated with benzofuran-2-ylboronic acid (104.6 mg, 0.65 mmol), K_2_CO_3_ (113.7 mg, 0.82 mmol), and Pd(PPh_3_)_4_ (38.2 mg, 0.33 mmol). After stirring at 100 °C for 24 h, the suspension was filtered through a Celite plug (2 cm). Purification by HPFC (SiO_2_; cHex/EtOAc, gradient 0 → 25% EtOAc) gave **13b** (206.6 mg, 82%) as a bright-yellow solid. *R*_f_ = 0.62 (SiO_2_; cHex/EtOAc 1:1); mp = 93 °C; ^1^H NMR (500 MHz, CDCl_3_): δ = 4.80 (dd, *J*_*gem*_ = 12.2 Hz, *J*_*5′a,4’*_ = 4.0 Hz, 1 H; H-5′a), 4.88 (m, 1 H; H-4’), 5.00 (dd, *J*_*gem*_ = 12.2 Hz, *J*_*5′b,4’*_ = 2.9 Hz, 1 H; H-5′b), 6.49 (t, *J*_*3′,2’*_ = *J*_*3′,4’*_ = 6.1 Hz, 1 H; H-3′), 6.74 (dd, *J*_*2′,3′*_ = 6.5 Hz, *J*_*2′,1’*_ = 5.3 Hz, 1 H; H-2’), 6.94 (dd, *J*_*2,1*_ = 8.6 Hz, *J*_*2,3*_ = 4.2 Hz, 1 H; H-2), 7.08 (d, *J*_*1′,2’*_ = 5.3 Hz, 1 H; H-1’), 7.29 (m, 1 H; H-7-benzofuryl), 7.32–7.39 (m, 4 H; H-A3, H-A5, H-5-benzofuryl, H-6-benzofuryl), 7.41 (m, 2 H; H–B3, H–B5), 7.46 (m, 2 H; H–C3, H–C5), 7.53 (m, 1 H; H-A4), 7.56 (d, *J*_*3,LR*_ = 0.9 Hz, 1 H; H-3-benzofuryl), 7.59 and 7.60 (2 × m, 2 × 1 H; H–B4, H–C4), 7.66 (bd, *J*_*1,2*_ = 8.6 Hz, 1 H; H-1), 7.76 (m, 1 H; H-4-benzofuryl), 7.92 (m, 2 H; H-A2, H-A6), 8.04 (m, 2 H; H–B2, H–B6), 8.07 (d, *J*_*5,6*_ = 9.2 Hz, 1 H; H-5), 8.12 (m, 2 H; H–C2, H–C6), 8.17 (d, *J*_*5,6*_ = 9.2 Hz, 1 H; H-6), 8.80 (dd, *J*_*3,2*_ = 4.2 Hz, *J*_*3,1*_ = 1.6 Hz, 1 H; H-3), 9.07 ppm (s, 1 H; H-9); ^13^C NMR (125.7 MHz, CDCl_3_): δ = 63.62 (C-5′), 71.04 (C-3′), 73.03 (C-2’), 80.25 (C-4’), 86.69 (C-1’), 110.18 (C-3-benzofuryl), 112.26 (C-7-benzofuryl), 112.41 (C-11a), 114.04 (C-11b), 114.71 (C-6), 120.60 (C-2), 122.53 (C-4-benzofuryl), 123.93 (C-5-benzofuryl), 124.33 (C-11c), 126.43 (C-6-benzofuryl), 128.35 (C-3a-benzofuryl), 128.64 and 128.69 (4 C; C-A3, C-A5, C–B3, C–B5), 128.74 (C-A1), 128.77 (2 C; C–C3, C–C5), 129.00 (C–B1), 129.57 (C–C1), 129.91, 129.95, and 131.01 (6 C; C-A2, C-A6, C–B2, C–B6, C–C2, C–C6), 132.11 (C-5), 133.64 and 133.83 (3 C, C-A4, C–B4, C–C4), 134.43 (C-1), 137.31 (C-6a), 145.86 (C-4a), 148.44 (C-3), 149.48 (C-11), 152.70 (C-9), 153.66 (C-2-benzofuryl), 155.48 (C-7a-benzofuryl), 155.89 (C-7a), 165.40 (C = O A), 165.63 (C = O B), 166.35 ppm (C = O C); IR (ATR, neat): *ṽ* = 3062 (w), 2932 (w), 1723 (s), 1601 (m), 1585 (w), 1561 (m), 1533 (w), 1516 (m), 1464 (w), 1450 (m), 1424 (w), 1377 (w), 1315 (m), 1257 (s), 1165 (m), 1109 (s), 1092 (s), 1068 (s), 1025 (m), 933 (w), 885 (m), 851 (w), 813 (w), 797 (m), 751 (w), 708 (s), 687 (m), 634 (w), 616 (w), 578 (w), 547 (w), 516 (w), 492 (w), 445 (w), 430 (w), 407 cm^–1^ (w); HR MS (ESI) for C_47_H_33_N_4_O_8_^+^ [M + H]^+^: calcd 781.22984, found 781.22987; for C_47_H_32_N_4_O_8_Na^+^ [M + Na]^+^: calcd 803.21178, found 803.21190.

### 11-Methyl-7-(2,3,5-tri-*O*-benzoyl-β-d-ribofuranosyl)pyrimido[5′,4′:4,5]pyrrolo[3,2-f]quinoline (**13c**)

A solution of the impure nucleoside **11** (267.7 mg, 75%, 0.29 mmol) in THF (7.2 mL) was treated with Pd(PPh_3_)_4_ (18.2 mg, 0.02 mmol) and Me_3_Al (0.43 mL, 2.0 M in toluene, 0.86 mmol). After stirring at 65 °C for 3 h, the resulting mixture was treated with MeOH (1 mL) before being concentrated *in vacuo*. Purification by HPFC (SiO_2_; cHex/EtOAc, gradient 0 → 50% EtOAc) gave **13c** (81.6 mg, 42%) as a brown sticky solid. *R*_f_ = 0.39 (SiO_2_; cHex/EtOAc 1:1); ^1^H NMR (500 MHz, CDCl_3_): δ = 3.32 (s, 3 H; C*Me*), 4.77 (dd, *J*_*gem*_ = 12.2 Hz, *J*_*5′a,4’*_ = 4.0 Hz, 1 Hz; H-5′a), 4.85 (bddd, *J*_*4′,3′*_ = 6.0 Hz, *J*_*4′,5′a*_ = 4.0 Hz, *J*_*4′,5′b*_ = 2.9 Hz, 1 Hz; H-4’), 4.98 (dd, *J*_*gem*_ = 12.2 Hz, *J*_*5′b,4’*_ = 2.9 Hz, 1 Hz; H-5′b), 6.47 (t, *J*_*3′,2’*_ = *J*_*3′,4’*_ = 6.3 Hz, 1 Hz; H-3′), 6.69 (dd, *J*_*2′,3′*_ = 6.6 Hz, *J*_*2′,1’*_ = 5.1 Hz, 1 Hz; H-2’), 7.05 (d, *J*_*1′,2’*_ = 5.1 Hz, 1 Hz; H-1’), 7.33, 7.40, and 7.46 (3 × m, 3 × 2 H: H-A3, H-A5, H–B3, H–B5, H–C3, H–C5), 7.53, 7.58, and 7.61 (3 × m, 3 × 1 H, H-A4, H–B4, H–C4), 7.58 (dd, *J*_*2,1*_ = 8.7 Hz, *J*_*2,3*_ = 4.2 Hz, H-2), 7.90, 8.02, and 8.10 (3 × m, 3 × 2 H; H-A2, H-A6, H–B2, H–B6, H–C2, H–C6), 8.06 (d, *J*_*5,6*_ = 9.2 Hz, H-5), 8.17 (d, *J*_*6,5*_ = 9.2 Hz, 1 Hz; H-6), 8.92 (s, 1 H; H-9), 8.95 (dd, *J*_*3,2*_ = 4.2 Hz, *J*_*3,1*_ = 1.6 Hz, 1 Hz; H-3), 9.23 ppm (bd, *J*_*1,2*_ = 8.7 Hz, 1 Hz; H-1); ^13^C{^1^H} NMR (125.7 MHz, CDCl_3_): δ = 28.89 (C*Me*), 63.58 (C-5′), 70.97 (C-3′), 73.03 (C-2’), 80.07 (C-4’), 86.69 (C-1’), 114.66 (C-11b), 114.93 (C-6), 115.10 (C-11a), 121.37 (C-2), 124.35 (C-11c), 128.62, 128.67, and 128.74 (6 C; C-A3, C-A5, C–B3, C–B5, C–C3, C–C5), 128.75, 129.00, and 129.58 (3 C; C-A1, C–B1, C–C1), 129.90, 129.93, and 129.99 (6 C; C-A2, C-A6, C–B2, C–B6, C–C2, C–C6), 131.29 (C-5), 133.27 (C-1), 133.62 and 133.80 (3 C; C-A4, C–B4, C–C4), 136.12 (C-4a), 146.05 (C-4a), 148.30 (C-3), 153.01 (C-9), 154.49 (C-7a), 159.80 (C-11), 165.38, 165.61, and 166.34 ppm (3 C; C = O); IR (ATR, neat): *ṽ* = 3062 (w), 2923 (w), 2853 (w), 1719 (m), 1601 (w), 1584 (w), 1554 (w), 1519 (w), 1492 (w), 1468 (w), 1451 (w), 1376 (w), 1314 (w), 1261 (s), 1176 (m), 1116 (m), 1092 (s), 1067 (s), 1024 (m), 1000 (m), 939 (m), 803 (w), 757 (w), 706 (s), 617 (m), 600 (m), 540 (m), 481 (m), 423 (m), 402 cm^–1^ (m); HR MS (ESI) for C_40_H_31_N_4_O_7_^+^ [M + H]^+^: calcd 679.21928, found 679.21830; for C_40_H_30_N_4_O_7_Na^+^ [M + Na]^+^: calcd 701.20123, found 701.20033.

### 11-(*N*,*N*-Dimethylamino)-7-(2,3,5-tri-*O*-benzoyl-β-d-ribofuranosyl)pyrimido[5′,4′:4,5]pyrrolo[3,2-*f*]quinoline (**13d**)

A suspension of the impure nucleoside **11** (250.9 mg, 75%, 0.27 mmol) in 2-propanol (10.7 mL) was treated with dimethylamine (0.27 mL, 2.0 M in THF, 0.54 mmol) and stirred at 60 °C for 24 h. Purification by HPFC (SiO_2_; Hex/EtOAc, gradient 0 → 30% EtOAc) gave **13d** (126.4 mg, 64%) as a sunflower-yellow solid. *R*_f_ = 0.40 (SiO_2_; cHex/EtOAc 1:1); mp = 240 °C; ^1^H NMR (500 MHz, CDCl_3_): δ = 3.08 (s, 6 H; N*Me*_2_), 4.78 (dd, *J*_*gem*_ = 12.0 Hz, *J*_*5′a,4’*_ = 4.1 Hz, 1 Hz; H-5′a), 4.83 (m, 1 Hz; H-4’), 4.96 (dd, *J*_*gem*_ = 12.0 Hz, *J*_*5′b,4’*_ = 2.9 Hz, 1 Hz; H-5′b), 6.47 (t, *J*_*3′,2’*_ = *J*_*3′,4’*_ = 6.2 Hz, 1 Hz; H-3′), 6.67 (bdd, *J*_*2′,3′*_ = 6.5 Hz, *J*_*2′,1’*_ = 5.2 Hz, 1 Hz; H-2’), 7.01 (d, *J*_*1′,2’*_ = 5.2 Hz, 1 Hz; H-1’), 7.34 (m, 2 Hz; H-A3, H-A5), 7.38 (m, 2 H; H–B3, H–B5), 7.46 (m, 2 H; H–C3, H–C5), 7.52 (dd, *J*_*2,1*_ = 8.5 Hz, *J*_*2,3*_ = 4.2 Hz, 1 Hz; H-2), 7.52 (m, 1 H; H-A4), 7.56 (m, 1 H; H–B4), 7.60 (m, 1 H; H–C4), 7.92 (m, 2 H; H-A2, H-A6), 7.94 (d, *J*_*5,6*_ = 9.2 Hz, 1 Hz; H-5), 8.00 (m, 2 H; H–B2, H–B6), 8.10 (d, *J*_*6,5*_ = 9.2 Hz, 1 Hz; H-6), 8.12 (m, 2 H, H–C2, H–C6), 8.61 (s, 1 H; H-9), 8.90 (dd, *J*_*7,6*_ = 4.2 Hz, *J*_*7,5*_ = 1.6 Hz, 1 Hz; H-7), 9.12 ppm (bdd, *J*_*1,2*_ = 8.6 Hz, *J*_*1,3*_ = 1.5 Hz, H-1); ^13^C NMR (125.7 MHz, CDCl_3_): δ = 41.76 and 41.86 (N*Me*_2_), 63.82 (C-5′), 71.03 (C-3′), 73.13 (C-2’), 80.07 (C-4’), 86.66 (C-1’), 102.70 (C-11a), 114.70 (C-6), 115.15 (C-11b), 120.57 (C-2), 124.50 (C-11c), 128.60, 128.62, and 128.74 (6 C; C-A3, C-A5, C–B3, C–B5, C–C3, C–C5), 128.88 (C-A1), 129.04 (C–B1), 129.17 (C-5), 129.65 (C–C1), 129.94, 129.95, and 129.98 (6 C; C-A2, C-A6, C–B2, C–B6, C–C2, C–C6), 133.56 and 133.73 (3 C; C-A4, C–B4, C–C4), 134.03 (C-6a), 134.89 (C-1), 145.79 (C-4a), 148.16 (C-3), 152.58 (C-9), 155.96 (C-7a), 163.13 (C-11), 165.38 (C = O A), 165.59 (C = O B), 166.40 ppm (C = O C); IR (ATR, neat): *ṽ* = 2920 (w), 2852 (w), 1724 (m), 1561 (m), 1517 (w), 1450 (w), 1420 (m), 1372 (w), 1315 (w), 1261 (s), 1176 (m), 1091 (s), 1068 (s), 1024 (s), 950 (m), 879 (w), 854 (w), 798 (m), 708 (s), 686 (m), 650 (m), 617 (m), 555 (m), 451 (s), 408 cm^–1^ (m); HR MS (ESI) for C_41_H_34_N_5_O_7_^+^ [M + H]^+^: calcd 708.24582, found 708.24575; for C_41_H_33_N_5_O_7_Na^+^ [M + Na]^+^: calcd 730.22777, found 730.22777.

### 11-(Furan-2-yl)-7-(β-d-ribofuranosyl)pyrimido[5′,4′:4,5]pyrrolo[3,2-*f*]quinoline (**14a**)

A suspension of **13a** (94.6 mg, 0.13 mmol) in MeOH (6.5 mL) was treated with NaOMe (14 μL, 25 wt % in MeOH, 0.08 mmol) and stirred at 60 °C for 24 h. Concentration *in vacuo* followed by coevaporation with MeOH (3 × 10 mL) and purification by HPFC (C_18_; water/MeOH, gradient 0 → 100% MeOH) gave **14a** (14.6 mg, 27%) as a brown film. *R*_f_ = 0.44 (SiO_2_; DCM/MeOH 9:1); [α]_D_^20^ = −65.7 (*c* = 0.023 in DMSO); ^1^H NMR (500 MHz, DMSO-*d*_6_): δ = 3.68–3.84 (m, 2 H; H-5′), 4.07 (q, *J*_*4′,3′*_ = *J*_*4′,5′*_ = 3.4 Hz, 1 H; H-4’), 4.31 (m, 1 H; H-3′), 4.86 (m, 1 H; H-2’), 5.19–5.43 (m, 3 H; OH-2′, OH-3′, OH-5′), 6.70 (d, *J*_*1′,2’*_ = 7.4 Hz, 1 H; H-1’), 6.89 (dd, *J*_*4,3*_ = 3.4 Hz, *J*_*4,5*_ = 1.8 Hz, 1 H; H-4-furyl), 7.336 (bd, *J*_*3,4*_ = 3.4 Hz, 1 H; H-3-furyl), 7.344 (bdd, *J*_*2,1*_ = 8.6 Hz, *J*_*2,3*_ = 4.2 Hz, 1 H; H-2), 7.50 (bdd, *J*_*1,2*_ = 8.7 Hz, *J*_*1,3*_ = 1.6 Hz, 1 H; H-1), 7.92 (dd, *J*_*5,4*_ = 1.9 Hz, *J*_*5,3*_ = 0.7 Hz, 1 H; H-5-furyl), 8.16 (d, *J*_*5,6*_ = 9.3 Hz, 1 H; H-5), 8.61 (d, *J*_*6,5*_ = 9.3 Hz, 1 H; H-6), 8.83 (dd, *J*_*3,2*_ = 4.2 Hz, *J*_*3,1*_ = 1.6 Hz, 1 H; H-3), 9.03 ppm (s, 1 H; H-9); ^13^C NMR (125.7 MHz, DMSO-*d*_6_): δ = 61.57 (C-5′), 70.05 (C-3′), 71.24 (C-2’), 85.79 (C-4’), 87.09 (C-1’), 110.72 (C-11a), 112.98 (C-11b), 113.20 and 113.34 (2 C; C-3-furyl, C-4-furyl), 117.06 (C-6), 120.94 (C-2), 123.48 (C-11c), 130.69 (C-5), 133.05 (C-1), 136.68 (C-6a), 145.03 (C-4a), 145.82 (C-5-furyl), 148.06 (C-3), 148.95 (C-11), 151.65 (C-2-furyl), 152.34 (C-9), 155.43 ppm (C-7a); IR (ATR, neat): *ṽ* = 3500–2500 (br w), 1669 (w), 1592 (m), 1558 (m), 1518 (m), 1466 (m), 1426 (m), 1372 (w), 1324 (m), 1242 (m), 1160 (m), 1119 (m), 1090 (m), 1045 (s), 1022 (s), 999 (s), 929 (m), 884 (m), 822 (m), 799 (m), 761 (m), 626 (m), 593 (m), 547 cm^–1^ (m); UV/vis (MeOH): λ_max_ (ε) = 255 (27500), 330 nm (5400 M^–1^ cm^–1^); HR MS (ESI) for C_22_H_19_N_4_O_5_^+^ [M + H]^+^: calcd 419.13555, found 419.13527; for C_22_H_18_N_4_O_5_Na^+^ [M + Na]^+^: calcd 441.11749, found 441.11721.

### 11-(Benzofuran-2-yl)-7-(β-d-ribofuranosyl)pyrimido[5′,4′:4,5]pyrrolo[3,2-*f*]quinoline (**14b**)

A suspension of **13b** (180.0 mg, 0.23 mmol) in MeOH (12.8 mL) was treated with NaOMe (14 μL, 25 wt % in MeOH, 0.08 mmol) and stirred at 60 °C for 18 h. Concentration *in vacuo* followed by coevaporation with MeOH (3 × 10 mL) and purification by HPFC (C_18_; water/MeOH, gradient 0 → 100% MeOH) gave **14b** (51.4 mg, 48%) as a bright-yellow solid. *R*_f_ = 0.46 (SiO_2_; DCM/MeOH 9:1); mp = 124 °C; [α]_D_^20^ = 46.4 (*c* = 0.115 in DMSO); ^1^H NMR (500 MHz, DMSO-*d*_6_): δ = 3.76 (bdt, *J*_*gem*_ = 12.0 Hz, *J*_*5′a,OH*_ = *J*_*5′,4’*_ = 4.3 Hz, 1 H; H-5′a), 3.81 (ddd, *J*_*gem*_ = 12.0 Hz, *J*_*5′b,OH*_ = 4.7 Hz, *J*_*5′b,4’*_ = 3.4 Hz, 1 H; H-5′b), 4.09 (q, *J*_*4′,3′*_ = *J*_*4′,5′*_ = 3.3 Hz, 1 H; H-4’), 4.33 (dt, *J*_*3′,2’*_ = 6.1 Hz, *J*_*3′,OH*_ = *J*_*3′,4’*_ = 3.1 Hz, 1 H; H-3′), 4.88 (q, *J*_*2′,1’*_ = *J*_*2′,OH*_ = *J*_*2′,3′*_ = 6.0 Hz, 1 H; H-2’), 5.28–5.33 (m, 2 H; OH-3′, OH-5′), 5.40 (d, *J*_*OH,2’*_ = 6.1 Hz, 1 H; OH-2’), 6.74 (d, *J*_*1′,2’*_ = 7.4 Hz, 1 H; H-1’), 6.96 (dd, *J*_*6,5*_ = 8.5 Hz, *J*_*6,7*_ = 4.2 Hz, 1 H; H-6), 7.34 (m, 1 H; H-7-benzofuryl), 7.39–7.44 (m, 2 H; H-5-benzofuryl, H-6-benzofuryl), 7.50 (bddd, *J*_*1,2*_ = 8.6 Hz, *J*_*1,3*_ = 1.7 Hz, *J*_*1,5*_ = 0.7 Hz, 1 H; H-1), 7.82 (d, *J*_*3,LR*_ = 0.9 Hz, 1 H; H-3-benzofuryl), 7.90 (m, 1 H; H-4-benzofuryl), 8.20 (dd, *J*_*5,6*_ = 9.2 Hz, *J*_*5,1*_ = 0.7 Hz, 1 H; H-5), 8.66 (d, *J*_*6,5*_ = 9.2 Hz, 1 H; H-6), 8.78 (dd, *J*_*3,2*_ = 4.2 Hz, *J*_*3,1*_ = 1.6 Hz, 1 H; H-3), 9.13 ppm (s, 1 H; H-9); ^13^C NMR (125.7 MHz, DMSO-*d*_6_): δ = 61.59 (C-5′), 70.07 (C-3′), 71.30 (C-2’), 85.84 (C-4’), 87.13 (C-1’), 109.59 (C-3-benzofuryl), 111.20 (C-11a), 111.84 (C-7-benzofuryl), 112.85 (C-11b), 117.09 (C-6), 120.45 (C-2), 122.70 (C-4-benzofuryl), 123.39 (C-11c), 123.97 (C-5-benzofuryl), 126.42 (C-6-benzofuryl), 127.99 (C-3a-benzofuryl), 131.03 (C-5), 133.19 (C-1), 137.04 (C-6a), 144.99 (C-4a), 148.14 (C-3), 148.56 (C-11), 152.39 (C-9), 153.48 (C-2-benzofuryl), 154.66 (C-7a-benzofuryl), 155.57 ppm (C-7a); IR (ATR, neat): *ṽ* = 3278 (br w), 2922 (w), 1655 (w), 1590 (w), 1559 (m), 1536 (m), 1517 (s), 1467 (m), 1447 (m), 1427 (m), 1370 (w), 1346 (w), 1299 (m), 1256 (m), 1167 (m), 1115 (s), 1088 (s), 1045 (s), 1017 (m), 960 (m), 932 (m), 915 (m), 883 (m), 853 (m), 815 (s), 796 (s), 751 (s), 697 (m), 634 (m), 612 (s), 580 (s), 568 (m), 548 (s), 518 (s), 498 (m), 485 (m), 451 (m), 432 (s), 419 (m), 405 cm^–1^ (m); UV/vis (MeOH): λ_max_ (ε) = 259 (38200), 355 (13600 M^–1^ cm^–1^); HR MS (ESI) for C_26_H_21_N_4_O_5_^+^ [M + H]^+^: calcd 469.15120, found 469.15095; for C_26_H_20_N_4_O_5_Na^+^ [M + Na]^+^: calcd 491.13314, found 491.13297.

### 11-Methyl-7-(β-d-ribofuranosyl)pyrimido[5′,4′:4,5]pyrrolo[3,2-*f*]quinoline (**14c**)

A suspension of **13c** (60.0 mg, 0.08 mmol) in MeOH (4.4 mL) was treated with NaOMe (5 μL, 25 wt % in MeOH, 0.04 mmol) and stirred at 60 °C for 18 h. Concentration *in vacuo* followed by coevaporation with MeOH (3 × 10 mL) and purification by HPFC (C_18_; water/MeOH, gradient 0 → 100% MeOH) gave **14c** (17.0 mg, 53%) as an off-white solid. *R*_f_ = 0.34 (SiO_2_; DCM/MeOH 9:1); mp = 256 °C; [α]_D_^20^ = −47.9 (*c* = 0.073 in DMSO); ^1^H NMR (500 MHz, DMSO-*d*_6_): δ = 3.30 (s, 3 H; C*Me*), 3.73 (bdd, *J*_*gem*_ = 11.9 Hz, *J*_*5′a,OH*_ = 5.5 Hz, *J*_*5′a,4’*_ = 3.8 Hz, 1 H; H-5′a), 3.77 (ddd, *J*_*gem*_ = 11.9 Hz, *J*_*5′b,OH*_ = 5.1 Hz, *J*_*5′b,4’*_ = 3.2 Hz, 1 H; H-5′b), 4.04 (q, *J*_*4′,3′*_ = *J*_*4′,5′*_ = 3.4 Hz, 1 H; H-4’), 4.29 (td, *J*_*3′,2’*_ = *J*_*3′,OH*_ = 5.3 Hz, *J*_*3′,4’*_ = 3.1 Hz, 1 H; H-3′), 4.83 (q, *J*_*2′,1’*_ = *J*_*2′,OH*_ = *J*_*2′,3′*_ = 6.5 Hz, 1 H; H-2’), 5.24 (d, *J*_*OH,3′*_ = 4.8 Hz, 1 H; OH-3′), 5.27–5.31 (m, 2 H; OH-2′, OH-5′), 6.70 (d, *J*_*1′,2’*_ = 7.4 Hz, 1 H; H-1’), 7.74 (dd, *J*_*2,1*_ = 8.6 Hz, *J*_*2,3*_ = 4.1 Hz, 1 H; H-2), 8.18 (bd, *J*_*5,6*_ = 9.2 Hz, 1 H; H-5), 8.61 (d, *J*_*6,5*_ = 9.2 Hz, 1 H; H-6), 8.91 (s, 1 H; H-9), 8.94 (dd, *J*_*3,2*_ = 4.1 Hz, *J*_*3,1*_ = 1.5 Hz, 1 H; H-3), 9.32 ppm (bd, *J*_*1,2*_ = 8.6 Hz, 1 H; H-1); ^13^C NMR (125.7 MHz, DMSO-*d*_6_): δ = 28.28 (C*Me*), 61.57 (C-5′), 70.00 (C-3′), 71.12 (C-2’), 85.65 (C-4’), 86.98 (C-1’), 113.56 and 113.68 (2; C-11a, C-11b), 117.03 (C-6), 121.52 (C-2), 123.44 (C-11c), 130.10 (C-5), 133.29 (C-1), 135.70 (C-6a), 145.15 (C-4a), 148.03 (C-3), 152.48 (C-9), 154.08 (C-7a); 159.82 ppm (C-11); IR (ATR, neat): *ṽ* = 3481 (w), 3225m–2275 (bw), 1587 (w), 1559 (m), 1521 (s), 1470 (m), 1441 (m), 1407 (w), 1370 (m), 1321 (m), 1307 (w), 1270 (w), 1245 (m), 1174 (m), 1124 (m), 1110 (m), 1080 (m), 1058 (s), 1033 (s), 1024 (s), 966 (m), 946 (w), 922 (m), 882 (m),858 (w), 819 (s), 790 (m), 771 (w), 729 (w), 688 (w), 660 (m), 632 (w), 603 (m), 581 (w), 564 (w), 538 (m), 514 (m), 438 cm^–1^ (m); UV/vis (MeOH): λ_max_ (ε) = 259 (50400), 319 (12900), 353 nm (4700 M^–1^ cm^–1^); HR MS (ESI) for C_19_H_19_N_4_O_4_^+^ [M + H]^+^: calcd 367.14063, found 367.14030; for C_19_H_18_N_4_O_4_Na^+^ [M + Na]^+^: calcd 389.12257, found 389.12229.

### 11-(*N*,*N*-Dimethylamino)-7-(β-d-ribofuranosyl)pyrimido[5′,4′:4,5]pyrrolo[3,2-*f*]quinoline (**14d**)

A suspension of **13d** (100.0 mg, 0.1413 mmol) in MeOH (7.1 mL) was treated with NaOMe (16 μL, 25 wt % in MeOH, 0.084 mmol) and stirred at 60 °C for 24 h. Concentration *in vacuo* followed by coevaporation with MeOH (3 × 10 mL) and purification by HPFC (C_18_; water/MeOH, gradient 0 → 100% MeOH) gave **14d** (35.5 mg, 64%) as a light-yellow solid. *R*_f_ = 0.42 (SiO_2_; DCM/MeOH 9:1); mp = 240 °C; [α]_D_^20^ = −5.0 (*c* = 0.119 in DMSO); ^1^H NMR (500 MHz, DMSO-*d*_6_): δ = 3.03 and 3.05 (2 × s, 2 × 3 H; N*Me*_2_), 3.70 (ddd, *J*_*gem*_ = 11.9 Hz, *J*_*5′a,OH*_ = 6.0 Hz, *J*_*5′a,4’*_ = 3.8 Hz, 1 H; H-5′a), 3.76 (ddd, *J*_*gem*_ = 11.9 Hz, *J*_*5′b,OH*_ = 4.8 Hz, *J*_*5′b,4’*_ = 3.2 Hz, 1 H; H-5′b), 4.03 (q, *J*_*4′,3′*_ = *J*_*4′,5′*_ = 3.3 Hz, 1 H; H-4’), 4.27 (dt, *J*_*3′,2’*_ = 6.3 Hz, *J*_*3′,4’*_ = *J*_*3′,OH*_ = 3.3 Hz, 1 H; H-3′), 4.87 (q, *J*_*2′,1’*_ = *J*_*2′,OH*_ = *J*_*2′,3′*_ = 6.3 Hz, 1 H; H-2’), 5.25 (d, *J*_*OH,3′*_ = 4.4 Hz, 1 H; OH-3′), 5.31 (d, *J*_*OH,2’*_ = 6.1 Hz, 1 H; OH-2’), 5.38 (t, *J*_*OH,5′*_ = 5.4 Hz, 1 H; OH-5′), 6.58 (d, *J*_*1′,2’*_ = 7.3 Hz, 1 H; H-1’), 7.68 (dd, *J*_*6,5*_ = 8.6 Hz, *J*_*6,7*_ = 4.2 Hz, 1 H; H-2), 8.07 (d, *J*_*9,10*_ = 9.2 Hz, 1 H; H-5), 8.47 (d, *J*_*10,9*_ = 9.2 Hz, 1 H; H-6), 8.55 (s, 1 H; H-9), 8.90 (dd, *J*_*7,6*_ = 4.2 Hz, *J*_*7,5*_ = 1.7 Hz, 1 H; H-3), 9.07 ppm (dm, *J*_*5,6*_ = 8.6 Hz,1 H; H-1); ^13^C NMR (125.7 MHz, DMSO-*d*_6_): δ = 40.99 and 41.40 (2 C; N*Me*_2_), 61.76 (C-5′), 70.20 (C-3′), 71.28 (C-2’), 85.67 (C-4’), 87.28 (C-1’), 101.06 (C-11a), 113.82 (C-11b), 116.54 (C-6), 120.80 (C-2), 123.50 (C-11c), 128.18 (C-5), 133.80 (C-6a), 134.15 (C-1), 144.99 (C-4a), 147.86 (C-3), 151.94 (C-9), 155.52 (C-7a), 162.41 ppm (C-11); IR (ATR, neat): *ṽ* = 3500–2000 (br w), 1561 (s), 1519 (s), 1462 (m), 1418 (m), 1396 (m), 1373 (m), 1352 (m), 1304 (m), 1284 (m), 1234 (m), 1182 (m), 1156 (m), 1121 (s), 1054 (s), 1031 (s), 993 (m), 958 (m), 919 (m), 901 (m), 877 (m), 858 (s), 817 (s), 797 (m), 736 (m), 714 (m), 694 (m), 661 (m), 634 (s), 599 (s), 584 (s), 553 (s), 540 (s), 522 (m), 484 (m), 444 (m), 431 (m), 415 cm^–1^ (m); UV/vis (MeOH): λ_max_ (ε) = 256 (29800), 298 (7700), 339 (7900), 359 nm (7100 M^–1^ cm^–1^); HR MS (ESI) for C_20_H_22_N_5_O_4_^+^ [M + H]^+^: calcd 396.16718, found 396.16689; for C_20_H_21_N_5_O_4_Na^+^ [M + Na]^+^: calcd 418.14912, found 418.14882.

### 11-Amino-7-(β-d-ribofuranosyl)pyrimido[5′,4′:4,5]pyrrolo[3,2-*f*]quinoline (**14e**)

A solution of the impure nucleoside **11** (245.0 mg, 75%, 0.26 mmol) in 1,4-dioxane (0.9 mL) was treated with aq. ammonia (2.0 mL, 25 wt % in water, 13.14 mmol) in a pressure glass tube. The mixture was stirred at 120 °C for 24 h, and the solution was cooled and concentrated *in vacuo*. Purification by HPFC (C_18_; water/MeOH, gradient 0 → 100% MeOH) gave **14e** (50.0 mg, 52%) as a beige solid. *R*_f_ = 0.31 (SiO_2_; DCM/MeOH 9:1); mp = 210–235 °C (decomp.); [α]_D_^20^ = 26.3 (*c* = 0.024 in DMSO); ^1^H NMR (500 MHz, DMSO-*d*_6_): δ = 3.69 (ddd, *J*_*gem*_ = 11.9 Hz, *J*_*5′a,OH*_ = 6.3 Hz, *J*_*5′a,4’*_ = 3.6 Hz, 1 H; H-5′a), 3.76 (ddd, *J*_*gem*_ = 11.9 Hz, *J*_*5′b,OH*_ = 4.6 Hz, *J*_*5′b,4’*_ = 3.1 Hz, 1 H; H-5′b), 4.02 (q, *J*_*4′,3′*_ = *J*_*4′,5′a*_ = *J*_*4′,5′b*_ = 3.2 Hz, 1 H; H-4’), 4.25 (td, *J*_*3′,2’*_ = *J*_*3′,OH*_ = 5.0 Hz, *J*_*3′,4’*_ = 2.8 Hz, 1 H; H-3′), 4.85 (q, *J*_*2′,1’*_ = *J*_*2′,OH*_ = *J*_*2′,3′*_ = 6.6 Hz, 1 H; H-2’), 5.20 (d, *J*_*OH,3′*_ = 4.8 Hz, 1 H; OH-3′), 5.26 (d, *J*_*OH,2’*_ = 6.7 Hz, 1 H; OH-2’), 5.47 (bt, *J*_*OH,5′a*_ = *J*_*OH,5′b*_ = 5.5 Hz, 1 H; OH-5′), 6.54 (d, *J*_*1′,2’*_ = 7.4 Hz, 1 H; H-1’), 7.24 (bs, 2 H; NH_2_), 7.66 (dd, *J*_*2,1*_ = 8.5 Hz, *J*_*2,3*_ = 4.2 Hz, 1 H; H-2), 8.03 (d, *J*_*5,6*_ = 9.2 Hz, 1 H; H-5), 8.37 (s, 1 H; H-9), 8.41 (d, *J*_*6,5*_ = 9.2 Hz, 1 H; H-6), 8.88 (dd, *J*_*3,2*_ = 4.2 Hz, *J*_*3,1*_ = 1.6 Hz, 1 H; H-3), 9.19 ppm (bd, *J*_*1,2*_ = 8.5 Hz, 1 H; H-1); ^13^C NMR (125.7 MHz, DMSO-*d*_6_): δ = 61.82 (C-5′), 70.26 (C-3′), 71.23 (C-2’), 85.68 (C-4’), 87.21 (C-1’), 98.72 (C-11a), 114.42 (C-11b), 116.32 (C-6), 120.66 (C-2), 122.90 (C-11c), 127.61 (C-5), 133.75 (C-1), 133.76 (C-6a), 144.99 (C-4a), 147.60 (C-3), 153.38 (C-9), 154.87 (C-7a), 159.13 ppm (C-11); IR (ATR, neat): *ṽ* = 3600–22500 (br w), 1633 (m), 1589 (m), 1574 (m), 1558 (m), 1522 (m), 1465 (m), 1441 (m), 1368 (m), 1317 (m), 1288 (m), 1188 (m), 1117 (s), 1035 (s), 961 (m), 930 (m), 903 (m), 888 (m), 859 (w), 811 (m), 793 (s), 767 (m), 697 (m), 672 (s), 602 (s), 578 (s), 541 (s), 511 (s), 466 (s), 424 cm^–1^ (s); UV/vis (MeOH): λ_max_ (ε) = 291 (13600), 330 nm (6800 M^–1^ cm^–1^); UV/vis (water): λ_max_ (ε) = 290 (11200), 333 nm (5700 M^–1^ cm^–1^); HR MS (ESI) for C_18_H_18_N_5_O_4_^+^ [M + H]^+^: calcd 368.13588, found 368.13508; for C_18_H_17_N_5_O_4_Na^+^ [M + Na]^+^: calcd 390.11782, found 390.11711.

### 11-Methoxy-7-(β-d-ribofuranosyl)pyrimido[5′,4′:4,5]pyrrolo[3,2-*f*]quinoline (**14f**)

A suspension of the impure nucleoside **11** (207.8 mg, 75%, 0.22 mmol) in MeOH (17.8 mL) was treated with sodium methoxide (0.25 mL, 30 wt % in MeOH, 1.31 mmol) and stirred at 60 °C for 4 h. Purification by HPFC (C_18_; water/MeOH, gradient 0 → 100% MeOH) gave **14f** (24.7 mg, 22%) as a beige solid. *R*_f_ = 0.58 (SiO_2_; DCM/MeOH 9:1); mp = 190–205 °C (decomp.); [α]_D_^20^ = −36.9 (c = 0.096 in DMSO); ^1^H NMR (600 MHz, DMSO-*d*_6_): δ = 3.72 (dd, 1 H, *J*_*gem*_ = 12.0 Hz, *J*_*5′a,4’*_ = 3.8 Hz; H-5′a), 3.97 (dd, 1 H, *J*_*gem*_ = 12.0 Hz, *J*_*5′b,4’*_ = 3.3 Hz; H-5′b), 4.04 (q, 1 H, *J*_*4′,3′*_ = *J*_*4′,5′*_ = 3.4 Hz; H-4’), 4.29 (dd, 1 H, *J*_*3′,2’*_ = 5.8 Hz, *J*_*3′,4’*_ = 3.1 Hz; H-3′), 4.34 (s, 3 H; O*Me*), 4.84 (dd, 1 H, *J*_*2′,1’*_ = 7.3 Hz, *J*_*2′,3′*_ = 5.8 Hz; H-2’), 6.65 (d, 1 H, *J*_*1′,2’*_ = 7.3 Hz; H-1’), 7.71 (dd, 1 H, *J*_*2,1*_ = 8.6 Hz, *J*_*2,3*_ = 4.2 Hz; H-2), 8.11 (dd, 1 H, *J*_*5,6*_ = 9.2 Hz, *J*_*5,1*_ = 0.7 Hz; H-5), 8.55 (d, 1 H, *J*_*6,5*_ = 9.2 Hz; H-6), 8.73 (s, 1 H; H-9), 8.92 (dd, 1 H, *J*_*3,2*_ = 4.2 Hz, *J*_*3,1*_ = 1.7 Hz; H-3), 9.77 ppm (ddd, 1 H, *J*_*1,2*_ = 8.6 Hz, *J*_*1,3*_ = 1.6 Hz, *J*_*1,5*_ = 0.7 Hz; H-1); 3 H (OH-2′, OH-3′, OH-5′) not detectable due to water content in the NMR sample; ^13^C NMR (150.9 MHz, DMSO-*d*_6_): δ = 54.59 (O*Me*), 61.62 (C-5′), 70.05 (C-3′), 71.32 (C-2’), 85.69 (C-4’), 87.35 (C-1’), 100.45 (C-11b), 113.40 (C-11a), 116.88 (C-6), 121.70 (C-2), 123.45 (C-11c), 128.91 (C-5), 133.95 (C-1), 134.35 (C-6a), 145.07 (C-4a), 148.24 (C-3), 153.18 (C-9), 155.62 (C-7a), 162.63 ppm (C-11); IR (ATR, neat): *ṽ* = 3203 (br m), 2929 (br m), 1673 (br w), 1592 (m), 1573 (m), 1556 (s), 1521 (s), 1468 (s), 1442 (m), 1429 (m), 1375 (m), 1311 (s), 1272 (m), 1207 (m), 1181 (m), 1115 (s), 1068 (s), 1039 (s), 984 (s), 960 (s), 918 (m), 887 (m), 857 (m), 812 (s), 793 (s), 715 (m), 685 (m), 636 (s), 586 (m), 542 (s), 506 (s), 466 (m), 435 (m), 410 cm^–1^ (m); UV/vis (MeOH): λ_max_ (ε) = 255 (38000), 282 (16300), 315 nm (9900 M^–1^ cm^–1^); HR MS (ESI) for C_19_H_19_N_4_O_5_^+^ [M + H]^+^: calcd 383.13555, found 383.13510; for C_19_H_18_N_4_O_5_Na^+^ [M + Na]^+^: calcd 405.11749, found 405.11707.

### 11-Methylthio-7-(β-d-ribofuranosyl)pyrimido[5′,4′:4,5]pyrrolo[3,2-*f*]quinoline (**14g**)

A solution of the impure nucleoside **11** (200.3 mg, 75%, 0.22 mmol) in THF (8.6 mL) was treated with NaSMe (92.3 mg, 1.32 mmol) and stirred at 60 °C for 18 h. Purification by HPFC (C_18_; water/MeOH, gradient 0 → 100% MeOH) gave **14g** (24.5 mg, 29%) as a pale-yellow solid. *R*_f_ = 0.50 (SiO_2_; DCM/MeOH 9:1); mp = 228–242 °C (decomp.); [α]_D_^20^ = −18.0 (*c* = 0.067 in DMSO); ^1^H NMR (500 MHz, DMSO-*d*_6_): δ = 2.84 (s, 3 H, S*Me*), 3.73 (bdd, *J*_*gem*_ = 12.0 Hz, *J*_*5′a,4’*_ = 3.7 Hz, 1 H; H-5′a), 3.79 (d, *J*_*gem*_ = 12.0 Hz, 1 H; H-5′b), 4.04 (q, *J*_*4′,3′*_ = *J*_*4′,5′*_ = 3.4 Hz, 1 H; H-4’), 4.29 (dd, *J*_*3′,2’*_ = 5.9 Hz, *J*_*3′,4’*_ = 3.1 Hz, 1 H; H-3′), 4.81 (dd, *J*_*2′,1’*_ = 7.3 Hz, *J*_*2′,3′*_ = 5.9 Hz, 1 H; H-2’), 5.23–5.47 (m, 3 H; OH-2′, OH-3′, OH-5′), 6.71 (d, *J*_*1′,2’*_ = 7.3 Hz, 1 H; H-1’), 7.74 (dd, *J*_*2,1*_ = 8.6 Hz, *J*_*2,3*_ = 4.2 Hz, 1 H; H-2), 8.17 (bd, *J*_*5,6*_ = 9.3 Hz, 1 H; H-5), 8.62 (d, *J*_*6,5*_ = 9.2 Hz, 1 H; H-6), 8.89 (s, 1 H; H-9), 8.94 (dd, *J*_*3,2*_ = 4.2 Hz, *J*_*3,1*_ = 1.6 Hz, 1 H; H-3), 9.95 ppm (dm, *J*_*1,2*_ = 8.7 Hz, 1 H; H-1); ^13^C NMR (125.7 MHz, DMSO-*d*_6_): δ = 13.86 (S*Me*); 61.54 (C-5′), 69.96 (C-3′), 71.24 (C-2’), 85.73 (C-4’), 87.15 (C-1’), 111.69 (C-11a), 113.26 (C-11b), 117.10 (C-6), 120.83 (C-2), 122.88 (C-11c), 130.15 (C-5), 133.82 (C-1), 135.15 (C-6a), 145.24 (C-4a), 148.12 (C-3), 151.84 (C-9), 152.80 (C-6a), 161.70 ppm (C-11); IR (ATR, neat): *ṽ* = 3600–2000 (m), 1665 (w),1591 (w), 1547 (m), 1516 (s), 1465 (m), 1437 (m), 1413 (m), 1369 (m), 1337 (m), 1303 (m), 1281 (w), 1260 (s), 1214 (m), 1178 (m), 1160 (m), 1123 (s), 1080 (s), 1067 (s), 1001 (s), 958 (m), 932 (s), 913 (m), 886 (m), 852 (m), 838 (s), 812 (s), 785 (s), 725 (m), 687 (s), 672 (s), 650 (s), 627 (s), 579 (s), 542 (s), 517 (s), 459 (s), 434 (s), 419 cm^–1^ (s); UV/vis (MeOH): λ_max_ (ε) = 252 (36600), 329 (10100), 359 nm (6600 M^–1^ cm^–1^); HR MS (ESI) for C_19_H_19_N_4_O_4_S^+^ [M + H]^+^: calcd 399.11270, found 399.11247; for C_19_H_18_N_4_O_4_SNa^+^ [M + Na]^+^: calcd 421.09464, found 421.09451.

### 11-Amino-7-(β-d-ribofuranosyl)pyrimido[5′,4′:4,5]pyrrolo[3,2-*f*]quinoline 5′-*O*-Triphosphate Bistriethylammonium Salt (**15**, **A^Q^TP**)

A solution of **14e** (25.1 mg, 0.07 mmol; dried under a vacuum at 45 °C overnight) in PO(OMe)_3_ (1.0 mL) was cooled to 0 °C and treated with POCl_3_ (13 μL, 0.14 mmol). After 2 h, the solution turned sunflower-yellow, and TLC showed the completion of the transformation toward the monophosphate (*R*_f_ = 0.00; SiO_2_, DCM/MeOH 9:1). In a separate flask, a solution of bis(tributylammonium) pyrophosphate (187.2 mg, 0.34 mmol) in MeCN (1.0 mL) was treated with NBu_3_ (0.08 mL, 0.34 mmol) and stirred for 5 min before being transferred to the monophosphate solution via a syringe. The resulting pale-yellow solution was stirred at 0 °C (water/ice) for 1 h until TLC showed the completion of the reaction (*R*_f_ = 0.32; SiO_2_, *i*PrOH/water/NH_4_OH 11:2:7). The mixture was concentrated *in vacuo* at 38 °C, coevaporated with water (2 × 10 mL), and dissolved in water (25 mL) followed by washing with CHCl_3_ (3 × 25 mL) to remove any traces of PO(OMe)_3_. Purification by prep. HPLC (Sepharose; 21.2 × 165 mm, flow rate = 12 mL min^–1^, water/800 mM TEAB, gradient 100:0 for 5 min, from 100:0 to 0:100 within 60 min) gave triphosphate **15** (32.4 mg, 59%) as a pale-yellow solid. Prep. HPLC: *t*_R_ = 30 min (Sepharose, 21.2 × 165 mm, flow rate = 12 mL min^–1^, water/800 mM TEAB, gradient 100:0 for 5 min, from 100:0 to 0:100 within 60 min); ^1^H NMR (500 MHz, D_2_O): δ = 4.40 (m, 1 H; H-4’), 4.42–4.48 (m, 2 H; H-5′), 4.70 (dd, *J*_*3′,2’*_ = 6.4 Hz, *J*_*3′,4’*_ = 4.1 Hz, 1 H; H-3′), 4.88 (t, *J*_*2′,1’*_ = *J*_*2′,3′*_ = 6.8 Hz, 1 H; H-2’), 6.33 (d, *J*_*1′,2’*_ = 7.1 Hz, 1 H; H-1’), 7.36 (m, 1 H; H-2), 7.69 (d, *J*_*5,6*_ = 9.2 Hz, 1 H; H-5), 8.02 (s, 1 H; H-11), 8.04 (d, *J*_*6,5*_ = 9.2 Hz, 1 H; H-6), 8.23 (bd, *J*_*1,2*_ = 7.9 Hz, 1 H; H-1), 8.55 ppm (m, 1 H; H-3); ^13^C NMR (125.7 MHz, D_2_O): δ = 67.45 (d, *J*_*C,P*_ = 5.6 Hz, 1 C; C-5′), 71.16 (C-3′), 73.20 (C-2’), 85.18 (d, *J*_*C,P*_ = 9.1 Hz, 1 C; C-4’), 88.85 (C-1’), 99.91 (C-11a), 115.86 (C-11b), 119.14 (C-6), 123.02 (C-2), 123.79 (C-11c), 128.14 (C-5), 134.89 (C-6a), 135.87 (C-1), 144.58 (C-4a), 148.72 (C-3), 154.30 (C-9), 155.61 (C-7a), 158.71 ppm (C-11); ^31^P NMR (202.4 MHz, D_2_O): δ = −21.74 (t, *J*_*β,α*_ = *J*_*β,γ*_ = 19.6 Hz, 1 P; P_β_), – 10.59 (d, *J*_*α,β*_ = 19.5 Hz, 1 P; P_α_), – 7.56 ppm (d, *J*_*γ,β*_ = 19.7 Hz, 1 P; P_γ_); UV/vis (water): λ_max_ (ε) = 258 (21000), 289 (12200), 333 nm (6600 M^–1^ cm^–1^); HR MS (ESI) for C_18_H_19_N_5_O_13_P_3_^+^ [M – H]^+^: calcd 606.01922, found 606.01923.

### Preparation of dsDNA template for Transcription (35DNA_A7)

A solution of complementary single-stranded DNA oligonucleotide (100 μM each) in water was heated up to 95 °C for 5 min in a thermal cycler and then slowly cooled down to 25 °C. The resulting dsDNA (50 μM) was used as the template **35DNA_A7** for the transcription reaction.

### Transcription Experiment with T7 Polymerase

Four *in vitro* transcription reactions were performed in parallel using the HiScribe T7 High yield RNA synthesis Kit: positive control, negative control, modification, and negative control for spectroscopy. Each reaction mixture (100 μL) contained Tris buffer (40 mM, pH 7.9), the three natural NTPs (7.5 mM each), the dsDNA template **35DNA_A7** (1 μg) and the T7 RNA polymerase (7.5 μL). Additionally, the positive control contained natural ATP (7.5 mM), the negative control contained water instead of ATP or **A**^**Q**^**TP**, the modification contained **A**^**Q**^**TP** (7.5 mM), and the negative control for spectroscopy contained the modified **A**^**Q**^**TP** (7.5 mM) but no T7 polymerase. All four reaction mixtures were incubated at 37 °C for 16 h. Then, the reactions were stopped by treatment with DNase I (0.1 U/μL) at 37 °C for 15 min followed by treatment with EDTA (0.05 M) at 70 °C for 10 min. Afterward, the mixtures were purified by the Monarch RNA Cleanup Kit (50 μg) resulting in solutions of 40 μL each.

Aliquots of the first three reactions (50 ng) were separated by denaturating PAGE (20%) with urea at 23 mV for 1 h and visualized by fluorescence imaging.

Aliquots of the positive control (**35RNA_A7**) and the modified RNA (**35RNA_A**^**Q**^**7**) were analyzed by UPLC-ESI-MS confirming the full transcription without any misincorporation (see Figures S1 and S2 in Supporting Information).

## Supporting Information Available

Azide/tetrazole equilibrium, cyclization of azide **9**, HPLC purity, UV and fluorescence spectra, MS spectra of oligonucleotides and copies of NMR spectra (PDF)
