# Supplementary material for: Synthesis and Biological Profiling of Quinolino-Fused 7-Deazapurine Nucleosides
Source: ACS Omega. 2024 Apr 27;9(18):20557–70. doi: 10.1021/acsomega.4c02031 (PMC11080019; doi:10.1021/acsomega.4c02031)
Supplement: Supplementary file 1 — ao4c02031_si_001.pdf [file ao4c02031_si_001.pdf]

Supporting Information

For

Synthesis and Biological Profiling of Quinolino-fused 7-Deazapurine Nucleosides

Marianne Fleuti,[a,b] Tania Sanchez-Quirante,[a,b] Lenka Poštová Slavětínská,[b] Eva Tloušťová,[b] Michal Tichý,[b] Soňa Gurská,[c] Petr Džubák,[c] Marián Hajdúch,[c] Michal Hocek*[a,b]

a *Department of Organic Chemistry, Faculty of Science, Charles University in Prague, Hlavova 8, CZ-12843 Prague 2, Czech Republic.*

b *Institute of Organic Chemistry and Biochemistry, Czech Academy of Sciences, Flemingovo nam. 2, CZ-16610 Prague 6, Czech Republic.*

c *Institute of Molecular and Translational Medicine, Palacky University and University Hospital in Olomouc, Faculty of Medicine and Dentistry, Hněvotínská 5, CZ-77515 Olomouc, Czech Republic.*

** author for correspondence: e-mail: hocek@uochb.cas.cz*

Content:

S1. Azide/Tetrazole equilibrium of intermediate **9** S2

S2. Cyclization of **9** towards the tetracyclic nucleobase **10** S2

S3. HPLC purity of final nucleosides **14a-g** S5

S4. UV & fluorescence spectra S5

S4.1. Measurements in MeOH S6

S4.2. Measurements in water S9

S5. Biochemistry S11

S5.1 UV and fluorescence maxima S11

S5.2. MS spectra of oligonucleotides S12

S6. NMR spectra S13

S7. References S37

S1. Azide/Tetrazole equilibrium of intermediate 9

In the intermediate **9**, the equilibrium between the azide **9a** and the tetrazole form **9b** highly depends on the polarity of the solvent. This equilibrium can be easily observed by ^{1}H NMR when looking at the shift of H-2 in the pyrimidine moiety.

The NMR study showed a preference of the azide **9a** in apolar solvents (benzene-d_6, $CDCl_3$) resp. in protonating solvents such as TFA (protonation of N-1 prevents tetrazole formation) which corresponds to the findings in our previous publication.[1] In THF-d_8, DMSO-d_6 and in DMF-d_7 both forms, azide **9a** and tetrazole **9b**, are detected in various ratios. (**Table S1**)

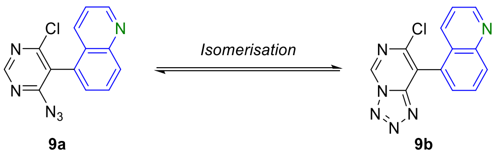

Table S1: ^{1}H NMR measurements to determine the azide/tetrazole equilibrium of **9**.

Measurement in	Azide **9a** (δ_{H-2} [ppm])	Tetrazole **9b** (δ_{H-2} [ppm])	Ratio **9a** : **9b**
Benzene-d_6	8.43	-	100 : 0
$CDCl_3$	8.82	-	100 : 0
THF-d_8	8.82	10.03	5 : 1
DMF-d_7	9.03	10.51	32 : 68
DMSO-d_6	8.99	10:40	23 : 77
TFA-d_1	9.08	-	100 : 0

The presence of the azide **9a** in $CDCl_3$ has been confirmed by IR measurement ($\tilde{\nu}$ = 2138 cm^{-1}).

S2. Cyclization of azide 9 towards the tetracyclic nucleobase 10

Three different cyclization options have previously been used to synthesize hetaryl-fused nucleobases: (1) thermal cyclization,[2] (2) catalytic cyclization with different rhodium catalysts[3] and (3) photocyclization with UV light.[2–4]

First, the azide/tetrazole equilibrium of **9** was shifted towards the azide **9a** – the reactive species for the cyclization – either by choosing an apolar solvent (see **Table S1** above) or by protonation of N-1 in the pyrimidine moiety. (**Scheme S1**)

Next, the reactive nitrene species is formed by decomposition of **9a** and release of N_2. Finally, intramolecular C–H insertion can occur resulting in the desired tetracyclic nucleobase **10**.

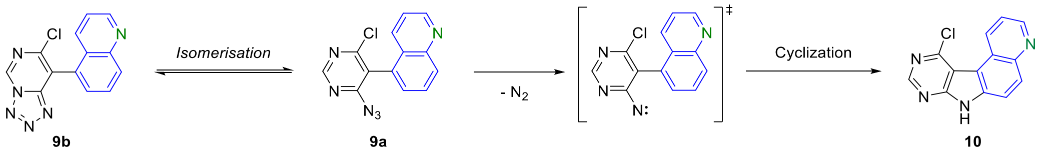

Scheme S1: Cyclization of **9**.

For the (2) Rh-catalyzed cyclization of **9**, three different catalysts have been tested: Rh_2esp_2,[5] Rhodium octanoate dimer ($Rh_2(O_2CC_7H_{15})_4$) and Rhodium heptafluorobutyrate dimer ($Rh_2(O_2CC_3F_7)_4$).[6] Previously in our group, the solvent system toluene/TFA (1:1) has been used to shift the azide/tetrazole equilibrium towards the azide for such reactions.[7] As the NMR study in benzene-d_6 showed the equilibrium of **9a**:**9b** to be 100:0, pure toluene as a solvent was tested as well (**Table S2**).

The conversion of the reaction mixture was determined by ^{1}H NMR in DMSO-d_6 after concentration *in vacuo*, addition of DMSO-d_6 and filtration through a syringe filter (nylon membrane, 0.45 µm pore size) by comparing H-2 in the pyrimidine moiety. The signal of H-2 in tetrazole **9b** is δ_{H-2} = 10.40 ppm. The signal of H-2 in nucleobase **10** is shifted upfield (δ_{H-2} = 8.80 ppm).

Table S2: Initial reaction screening for the Rh-catalyzed cyclization of **9**.

Entry	Conditions	Temp [°C]	Time	conversion by crude NMR
1	10 mol% $Rh_2(esp)_2$, toluene (0.2 M)	90	24 h	no reaction (**9** only)
2	10 mol% $Rh_2(esp)_2$, 50 wt% mol. sieves (4 Å), toluene (0.2 M)	90	24 h	no reaction (**9** only)
3	10 mol% $Rh_2(esp)_2$, toluene/TFA 1:1 (0.2 M)	90	24 h	decomposition
4	10 mol% $Rh_2(esp)_2$, 50 wt% mol. sieves (4 Å), toluene/TFA 1:1 (0.2 M)	90	24 h	decomposition
5	10 mol% $Rh_2(O_2CC_7H_{15})_4$, toluene (0.2 M)	90	24 h	no reaction (**9** only)
6	10 mol% $Rh_2(O_2CC_7H_{15})_4$, 50 wt% mol. sieves (4 Å), toluene (0.2 M)	90	24 h	no reaction (**9** only)
7	10 mol% $Rh_2(O_2CC_7H_{15})_4$, toluene/TFA 1:1 (0.2 M)	90	24 h	decomposition
8	10 mol% $Rh_2(O_2CC_7H_{15})_4$, 50 wt% mol. sieves (4 Å), toluene/TFA 1:1 (0.2 M)	90	24 h	decomposition
9	10 mol% $Rh_2(O_2CC_3F_7)_4$, toluene (0.2 M)	90	24 h	no reaction (**9** only)
10	10 mol% $Rh_2(O_2CC_3F_7)_4$, 50 wt% mol. sieves (4 Å), toluene (0.2 M)	90	24 h	no reaction (**9** only)
11	10 mol% $Rh_2(O_2CC_3F_7)_4$, toluene/TFA 1:1 (0.2 M)	90	24 h	decomposition
12	10 mol% $Rh_2(O_2CC_3F_7)_4$, 50 wt% mol. sieves (4 Å), toluene/TFA 1:1 (0.2 M)	90	24 h	decomposition

Independent of the catalyst, all the reactions in toluene did not work at all, only starting material was recovered. On the other hand, all the reactions in toluene/TFA led to decomposition of the starting azide **9**. No product **10** was observed in any rhodium-catalyzed cyclization reaction (**Table S2**).

Previously in our group, the (3) photocyclization has been successful towards a variety of hetaryl-fused 7-deazapurines.[2–4]

The initial photocyclization of **9** under UV light (λ_{max} = 254 nm, 4 W) in TFA at r.t. under ambient atmosphere did not give the desired product **10** after 48 hours, instead it resulted in decomposition. (**Table S3**, Entry 1)

Table S3: Photocyclization of **9** toward the tetracyclic nucleobase **10**.

Entry	Conditions	Temp [°C]	Time	**10**	recovered **9**
1	UV (λ_{max} = 254 nm), TFA (0.05 M)	20	48 h	0%	-
2	UV (λ_{max} = 254 nm), DCM (0.025 M)	20	96 h	39%, inseparable mixture of **10** and **16** (3:2)	13%
3	UV (λ_{max} = 275 nm), DCM (0.025 M)	20	96 h	19%, inseparable mixture of **10** and **16** (3:2)	7%
4	5 mol% $Ir(ppy)_3$, UV (λ_{max} = 400 nm), DCM (0.025 M)	20	96 h	0% (31% of **16**)	
5	5 mol% $[Ir(ppy)_2(tbbpy)]PF_6$, UV (λ_{max} = 400 nm), DCM (0.025 M)	20	6 d	0%	
6	UV (λ_{max} = 254 nm), THF (0.025 M)	-10, then 20	96 h	44%, inseparable mixture of **10** and **16** (4:1)	
7	UV (λ_{max} = 254 nm), THF (0.025 M)	20	6 days	19%	-
8	1.0 equiv. pyrene, UV (λ_{max} = 254 nm), THF (0.025 M)	20	72 h	26%	-

The photocyclization of **9** was repeated in DCM under argon resulting in an inseparable mixture of nucleobase **10** and side product **16**. (**Table S3**, Entry 2 and 3) Interestingly, the amine **16** has the same R_f value as the nucleobase **10**. So, a selective reaction towards **10** without the formation of the amine **16** is pivotal. When using a photosensitizer, such as $Ir(ppy)_3$, the triplet nitrene can be selectively accessed which resulted in the exclusive formation of the amine **16** (31%). (**Table S3**, Entry 4). Apparently, the cyclization of **9** proceeds via a singlet nitrene towards the nucleobase **10**. If the triplet nitrene is formed, it results in the formation of the amine **16**. (**Scheme S2**)

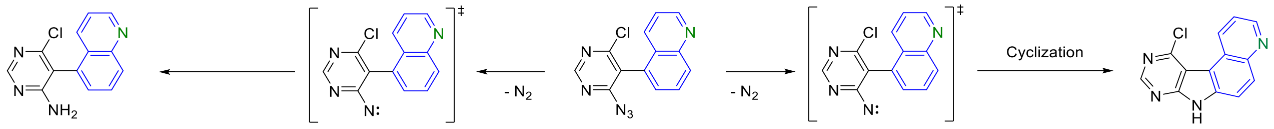

Scheme S2: Decomposition of the azide **9a** and formation of the singlet nitrene resulting in the cyclized product 10. If triplet nitrene is formed, the reaction results in the formation of the amine **16**.

One previous photocyclization in our group was performed in THF at –20 °C.[2c] The same conditions were applied and the reaction was still incomplete after 4 days. Warming the reaction to r.t. and stirring it for additional 48 hours resulted in an inseparable mixture of the nucleobase **10** and the amine **16** (4:1, Entry 6). The photocyclization in THF at r.t. resulted in 19% of **10** after 6 days (Entry 7). When using pyrene, a singlet photosensitizer, the reaction was complete after 72 hours and resulted in 26% of **10** (Entry 8).

S3. HPLC purity of final nucleosides 14a-g

The purity of final nucleosides **14a-g** was confirmed by analytical UPLC on an *Agilent 1260 Infinity II LC* system with a *Agilent 1260 Photodiode Array Detector* using a *KinetexEVO* C_{18} *100 Å* column (2.1 × 150 mm) from *Phenomenex*. Samples were dissolved in DMSO (1 µL injection volume). Two methods were used (flow 0.2 mL/min):

A) water (0.1% formic acid)/MeOH gradient 100:0 for 0.5 min, from 100:0 to 0:100 within 7 min, 0:100 for 2 min, from 0:100 to 100:0 within 0.1 min, 100:0 for 4 min

B) water (0.1% formic acid)/MeCN gradient 100:0 for 0.5 min, from 100:0 to 0:100 within 7 min, 0:100 for 2 min, from 0:100 to 100:0 within 0.1 min, 100:0 for 4 min

Table S4: HPLC purity of final nucleoside **14a-g**.

Compound	Method A (water → MeOH)		Method B (water → MeCN)	
	Retention time t_R [min]	Purity [%]	Retention time t_R [min]	Purity [%]
**14a**	8.30	95.14	6.47	95.42
**14b**	9.59	95.45	7.38	96.10
**14c**	7.19	99.26	5.87	98.70
**14d**	7.58	96.24	6.16	97.48
**14e**	6.43	96.47	5.58	95.15
**14f**	7.87	99.22	7.87	99.24
**14g**	8.96	95.13	7.57	96.79

S4. UV & fluorescence spectra

UV/Vis spectra of the final nucleosides **14a-g** were measured in methanol (HPLC grade) on a *Varian Cary 100 Bio* UV-Visible spectrophotometer in the range 250–800 nm using transparent 1.5 mL quartz cuvettes. The absorption coefficient ε was calculated using the Lambert-Beer equation $A = c \cdot l \cdot \varepsilon$, where A is the absorbance of the sample, c the exact concentration of the sample, l the length of the cuvette (1 cm). The samples were measured at three different concentrations and its average absorption coefficient was calculated.

Fluorescence spectra were recorded on a *Fluoromax 4* spectrofluorimeter from *HORIBA Scientific*. The sample concentration was adjusted to have an UV absorbance of 0.05–0.10. The excitation was performed at the absorption maximum with the highest wavelength λ_{abs} with the slit set at 2 nm. The emission spectra were recorded from $\lambda_{abs} + 20\ nm$ to $2 \times \lambda_{abs} - 20\ nm$ with a 2 nm slit opening. For each nucleoside **14a–g**, three samples of the same concentration were prepared and measured.

For nucleosides with an absorption maximum above 300 nm, their fluorescence quantum yield Φ_f was determined with quinine sulfate as a standard (equation S1),[8]

$$\Phi_{f,x} = \Phi_{f,QS} \left[\frac{F_x \left(1-10^{-A_x}\right)}{F_{QS} \left(1-10^{-A_{QS}}\right)} \right] \frac{\eta_x^2}{\eta_{QS}^2} \qquad \text{(Eq. S1)}$$

where $\Phi_{f,x}$ is the quantum yield of the sample x, $\Phi_{f,QS}$ is the quantum yield of the standard quinine sulfate in 0.1 M H_2SO_4 (Lit.[9]: 0.546), F_x and F_{QS} are the integrals of the emission spectra between $\lambda_{abs} + 20\ nm$ to $2 \times \lambda_{abs} - 20\ nm$, A_x and A_{QS} are the absorbance coefficients maxima during the

UV/Vis measurement ($\lambda_{abs} = \lambda_{ex}$), η_X is the refractive index of methanol at the excitation wavelength,[10] η_{QS} is the refractive index of water at the excitation wavelength.[11]

Standard deviations for the extinction coefficient ε and for the fluorescence quantum yield Φ_f are calculated using the *STEV.P* function in excel.

S4.1. Measurements in MeOH

11-(Furan-2-yl)-7-(*β*-D-ribofuranosyl)pyrimido[5',4':4,5]pyrrolo[3,2-*f*]quinoline (14a)

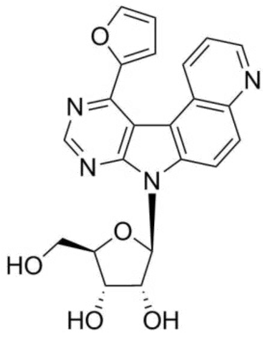

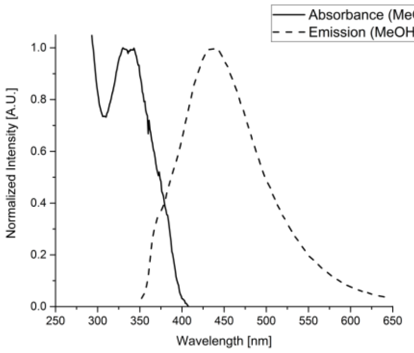

Absorption<br>max. λ_{abs} [nm]	Extinction coefficient<br>ε [M^{-1} cm^{-1}]	Emission<br>max. λ_{em} [nm]	Fluorescence Quantum yield<br>Φ_f
330	5400 ± 600	436	0.073 ± 0.025
255	27500 ± 600		

11-(Benzofuran-2-yl)-7-(*β*-D-ribofuranosyl)pyrimido[5',4':4,5]pyrrolo[3,2-*f*]quinoline (14b)

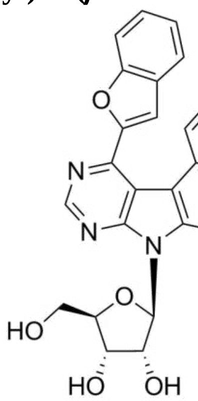

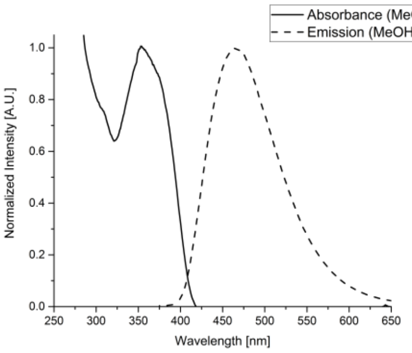

Absorption<br>max. λ_{abs} [nm]	Extinction coefficient<br>ε [M^{-1} cm^{-1}]	Emission<br>max. λ_{em} [nm]	Fluorescence Quantum yield<br>Φ_f
355	13600 ± 500	466	0.510 ± 0.172
259	38200 ± 1000		

11-Methyl-7-(*β*-D-ribofuranosyl)pyrimido[5',4':4,5]pyrrolo[3,2-*f*]quinoline (14c)

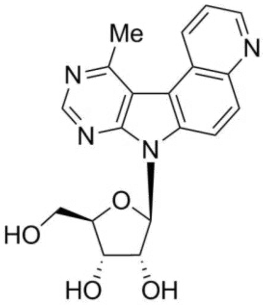

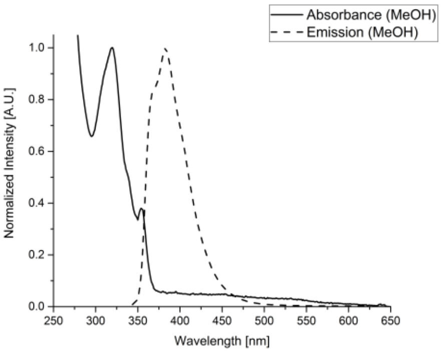

Absorption max. λ_{abs} [nm]	Extinction coefficient ε [M^{-1} cm^{-1}]	Emission max. λ_{em} [nm]	Fluorescence Quantum yield Φ_f
353	4800 ± 100		
319	12900 ± 100	383	0.4024 ± 0.013
259	50300 ± 300		

11-(*N*,*N*-Dimethylamino)-7-(*β*-D-ribofuranosyl)pyrimido[5',4':4,5]pyrrolo[3,2-*f*]quinoline (14d)

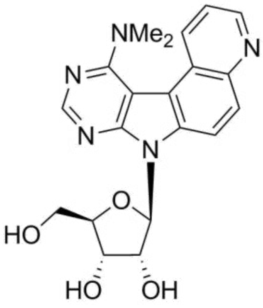

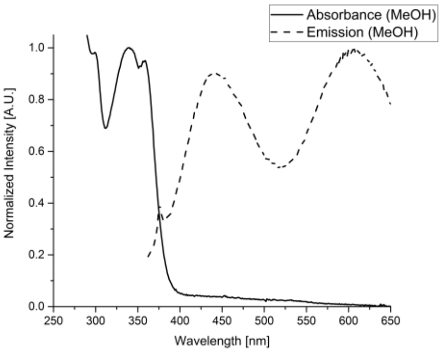

Absorption max. λ_{abs} [nm]	Extinction coefficient ε [M^{-1} cm^{-1}]	Emission max. λ_{em} [nm]	Fluorescence Quantum yield Φ_f
359	7100 ± 100		
339	7900 ± 100	439 and 610	0.136 ± 0.005
298	7700 ± 100		
256	29800 ± 200		

11-Amino-7-(*β*-D-ribofuranosyl)pyrimido[5',4':4,5]pyrrolo[3,2-*f*]quinoline (14e)

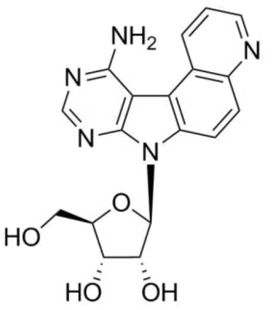

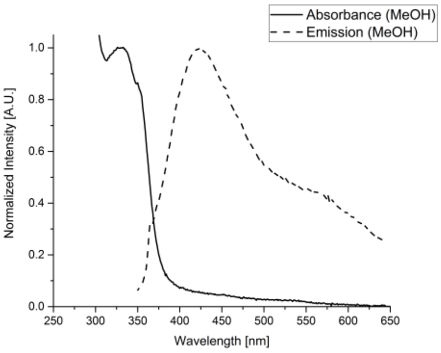

Absorption max. λ_{abs} [nm]	Extinction coefficient ε [M^{-1} cm^{-1}]	Emission max. λ_{em} [nm]	Fluorescence Quantum yield Φ_f
330	6800 ± 300	426	0.046 ± 0.002
291	13600 ± 400		

11-Methoxy-7-(*β*-D-ribofuranosyl)pyrimido[5',4':4,5]pyrrolo[3,2-*f*]quinoline (14f)

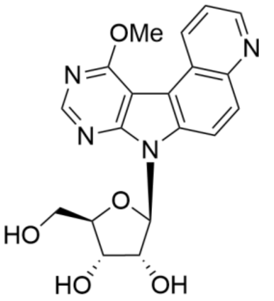

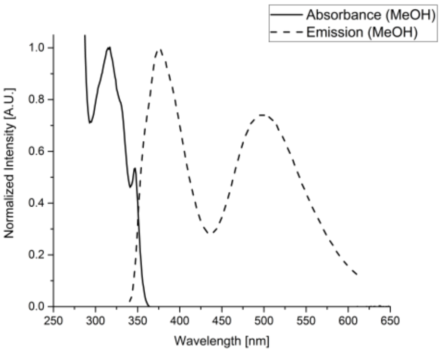

Absorption max. λ_{abs} [nm]	Extinction coefficient ε [M^{-1} cm^{-1}]	Emission max. λ_{em} [nm]	Fluorescence Quantum yield Φ_f
346	5700 ± 500		
315	9900 ± 700	376 and 499	0.163 ± 0.037
282	16300 ± 1100		
255	38000 ± 2300		

11-Methylthio-7-(*β*-D-ribofuranosyl)pyrimido[5',4':4,5]pyrrolo[3,2-*f*]quinoline (14g)

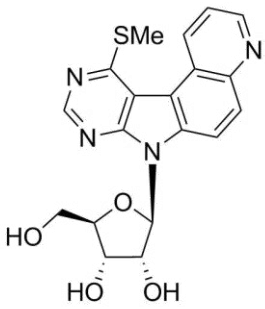

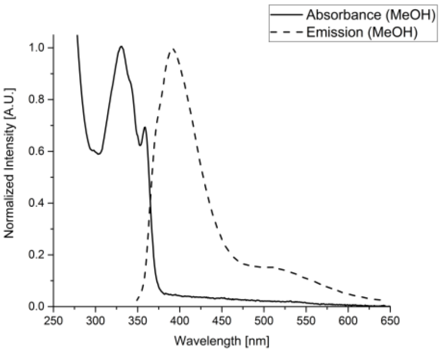

Absorption max. λ_{abs} [nm]	Extinction coefficient ε [M^{-1} cm^{-1}]	Emission max. λ_{em} [nm]	Fluorescence Quantum yield Φ_f
359	6600 ± 400		
329	10100 ± 600	390	0.255 ± 0.015
252	36600 ± 1800		

S6.2. Measurements in water

The nucleoside **14e**, the triphosphate **15** and all biochemistry products were measured in MilliQ water as described above. The fluorescence quantum yield Φ_f was determined with an excitation wavelength of λ_{ex} = 333 nm and a 2 nm slit opening. The emission spectra were recorded from 353 nm to 646 nm with the same 2 nm slit opening.

11-Amino-7-(*β*-D-ribofuranosyl)pyrimido[5',4':4,5]pyrrolo[3,2-*f*]quinoline (14e)

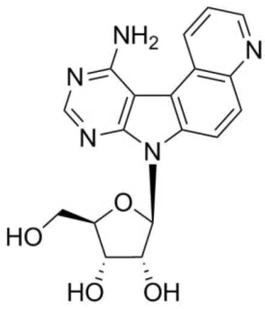

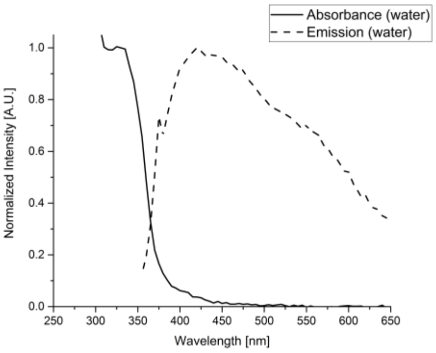

Absorption max. λ_{abs} [nm]	Extinction coefficient ε [M^{-1} cm^{-1}]	Emission max. λ_{em} [nm]	Fluorescence Quantum yield Φ_f
333	5700 ± 100	420	0.02855 ± 0.00079
290	11200 ± 100		

11-Amino-7-(*β*-D-ribofuranosyl)pyrimido[5',4':4,5]pyrrolo[3,2-*f*]quinoline 5'-*O*-Triphosphate Bistriethylammonium Salt (15, A^{Q}TP)

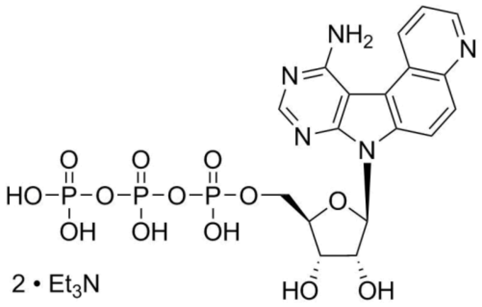

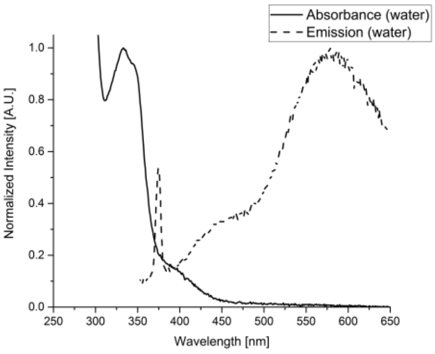

Absorption<br>max. λ_{abs} [nm]	Extinction coefficient<br>ε [M^{-1} cm^{-1}]	Emission<br>max. λ_{em} [nm]	Fluorescence Quantum yield<br>Φ_f
333	6600 ± 100	579	0.00004 ± 0.00001
289	12600 ± 400		
258	21000 ± 500		

35RNA_A7 (positive control)

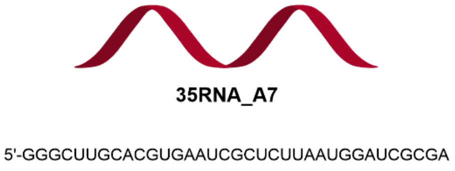

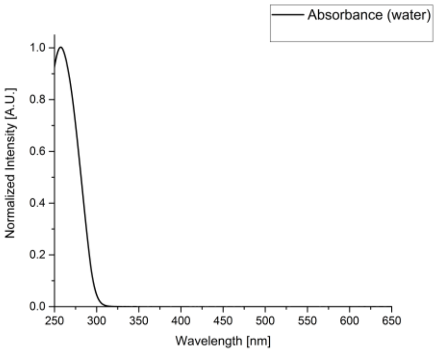

Absorption<br>max. λ_{abs} [nm]	Emission<br>max. λ_{em} [nm]	Fluorescence Quantum yield<br>Φ_f
256		

35RNA_A^{Q}7

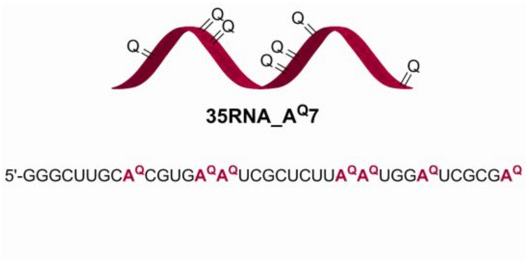

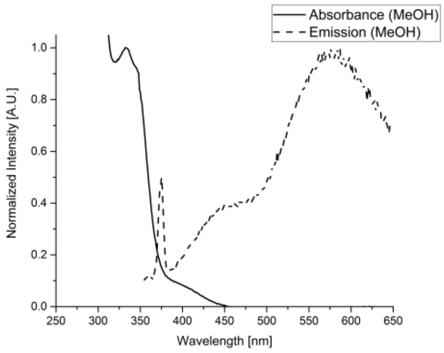

Absorption<br>max. λ_{abs} [nm]	Emission<br>max. λ_{em} [nm]	Fluorescence Quantum yield<br>Φ_f
333	576	0.01120 ± 0.00009
256		

S5. Biochemistry

S5.1. UV and fluorescence maxima

Table S5: UV and fluorescence maxima of nucleoside **14e**, triphosphate **15** (**A^{Q}TP**), natural ATP and all transcription products in water.

	Absorption<br>max. λ_{abs} (ε) [nm (M^{-1} cm^{-1})]	Emission<br>max. λ_{em} [nm]	Quantum Yield<br>Φ_f
Nucleoside **14e**	290 (11200), 333 (5700)	420	0.02855
Triphosphate **15** (**A^{Q}TP**)	258 (21000), 290 (12600), 333 (6600)	579	0.00005
Natural ATP	256 (6300)	-	
Positive control (**35RNA_A7**)	256	-	
Negative control	-	-	
Modification (**35RNA_A^{Q}7**)	256, 333	576	0.01120

UV and fluorescence maxima were measured in MilliQ water. The emission spectra were recorded after excitation at λ_{ex} = 333 nm. The molar extinction coefficient ε was not calculated for the transcription products as their absolute concentration cannot be determined by *Nanodrop*. Fluorescence quantum yields Φ_f were determined using quinine sulfate in 0.1 M H_2SO_4 as a standard (Φ_f = 0.546 at 25 °C).[8]

S5.2. MS spectra of oligonucleotides

LC-ESI-MS analysis of oligonucleotides was carried out on an *Agilent 1260 Infinity II LC* system with an *Agilent InfinityLab LS/MSD XT* Detector using a *BioZen* C_{18} *100 Å* column (2.1 × 150 mm) from *Phenomenex* with the mobile phases A (12.2 mM Et_3N, 300 mM HFIP in water) and B (12.2 mM Et_3N, 300 mM HFIP in 100% MeOH) and a gradient from 95:5 to 0:100 within 10 min. Deconvolutions of the LC-ESI-MS spectra were carried out using a *UniDec* program.

Positive Control: 35RNA_A7

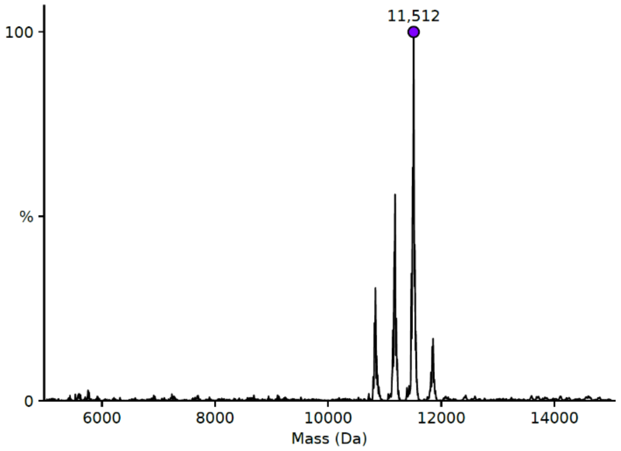

Figure S1: MS spectrum after LC-ESI-MS analysis of the positive control (**35RNA_A7**) in the transcription experiment. Deconvoluted mass spectrum.

	Sequence	Calcd. Mass [Da]	Found mass [Da]
**35RNA_A7**	5’-pppGGGCUUGC**A**CGUG**AA**UCGCUCUU**AA**UGG**A**UCGCG**A**	11471	11512 [+K^+]

ppp – triphosphate residue at the 5’ terminus.

Modified RNA: 35RNA_A^{Q}7

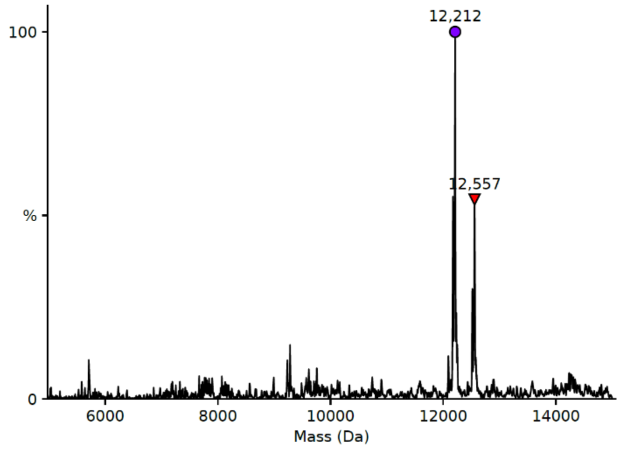

Figure S2: MS spectrum after LC-ESI-MS analysis of the modified **35RNA_A^{Q}7** in the transcription experiment. Deconvoluted mass spectrum.

	Sequence	Calcd. Mass [Da]	Found mass [Da]
**35RNA_A^{Q}7**	5’-pppGGGCUUGC**A**^{Q}CGUG**A**^{Q}**A**^{Q}UCGCUCUU**A**^{Q}**A**^{Q}UGG**A**^{Q}**A**^{Q}UCGCG**A**^{Q}	12174	12212 [+K^+]<br>12557 [+G,+K^+]

ppp – triphosphate residue at the 5’ terminus.

S6. NMR spectra

5-(4,6-Dichloropyrimidin-5-yl)quinoline (8)

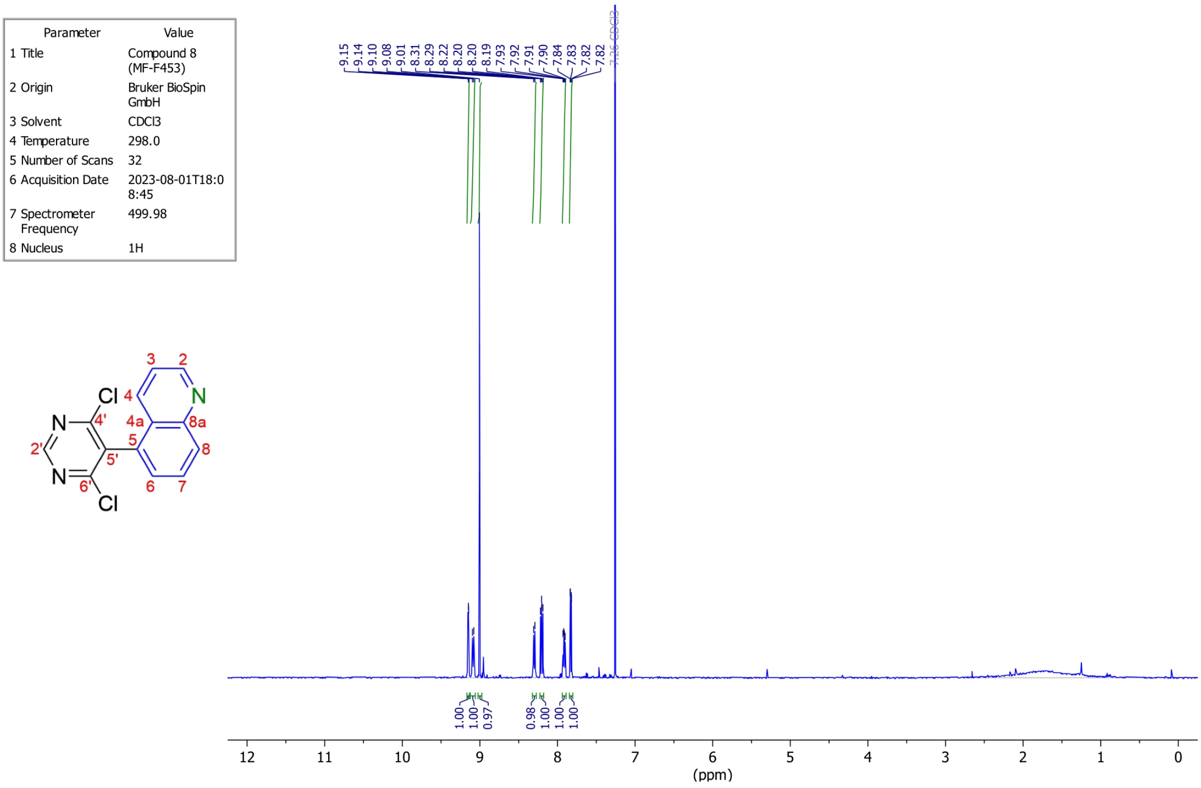

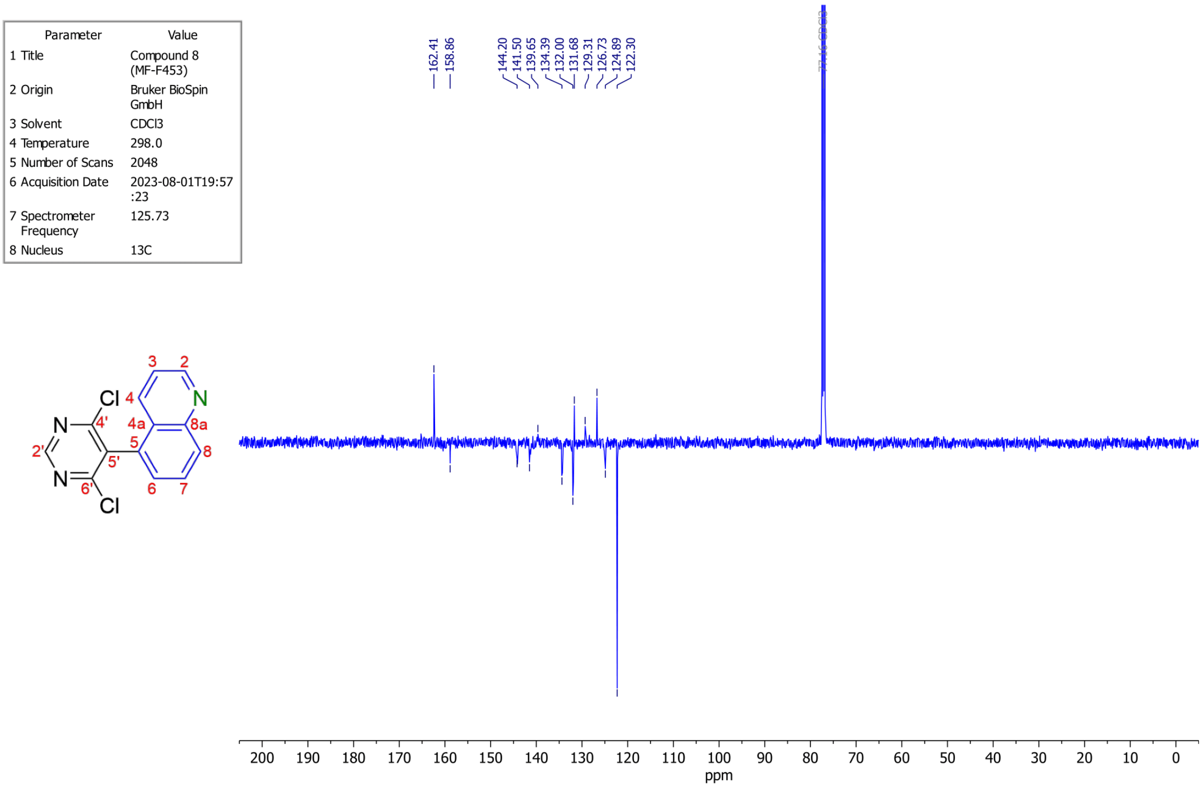

Figure S3. ^{1}H and ^{13}C NMR spectra of compound **8** in $CDCl_3$.

5-(4-Azido-6-chloropyrimidin-5-yl)quinoline (9a)

Parameter	Value
1 Title	Compound 9a (MF-E350)
2 Origin	Bruker BioSpin GmbH
3 Solvent	CDCl3
4 Temperature	298.0
5 Number of Scans	32
6 Acquisition Date	2022-03-05T19:38:00
7 Spectrometer Frequency	499.98
8 Nucleus	1H

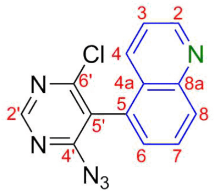

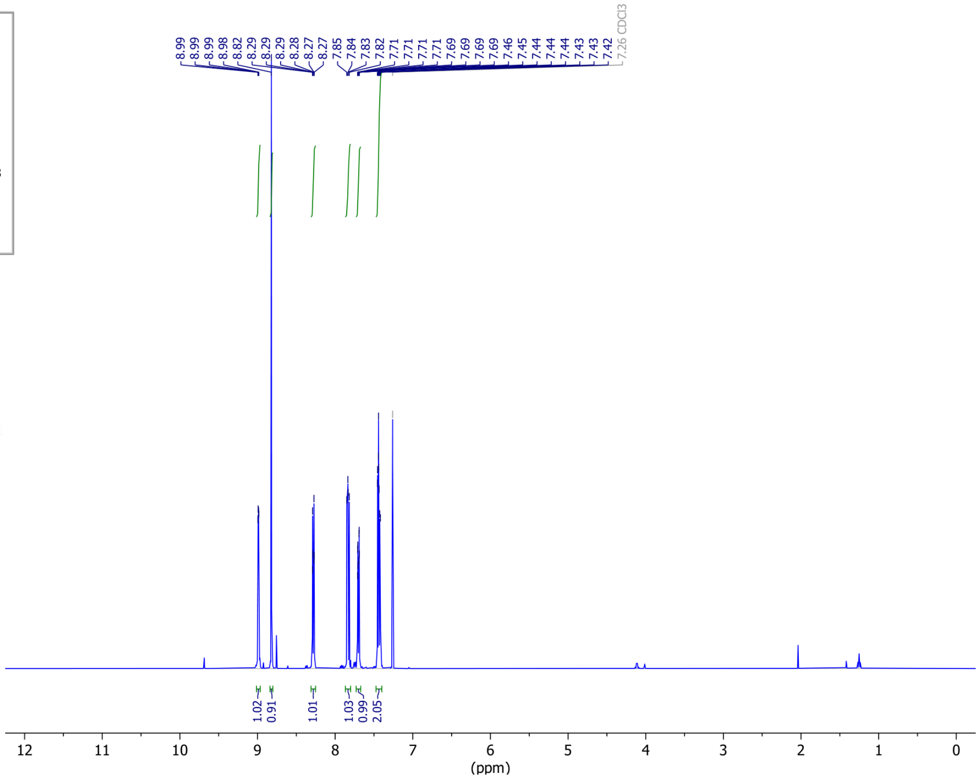

Parameter	Value
1 Title	Compound 9a (MF-E350)
2 Origin	Bruker BioSpin GmbH
3 Solvent	CDCl3
4 Temperature	298.0
5 Number of Scans	1024
6 Acquisition Date	2022-03-05T20:32:48
7 Spectrometer Frequency	125.73
8 Nucleus	13C

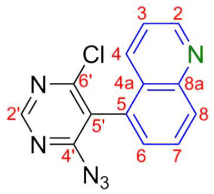

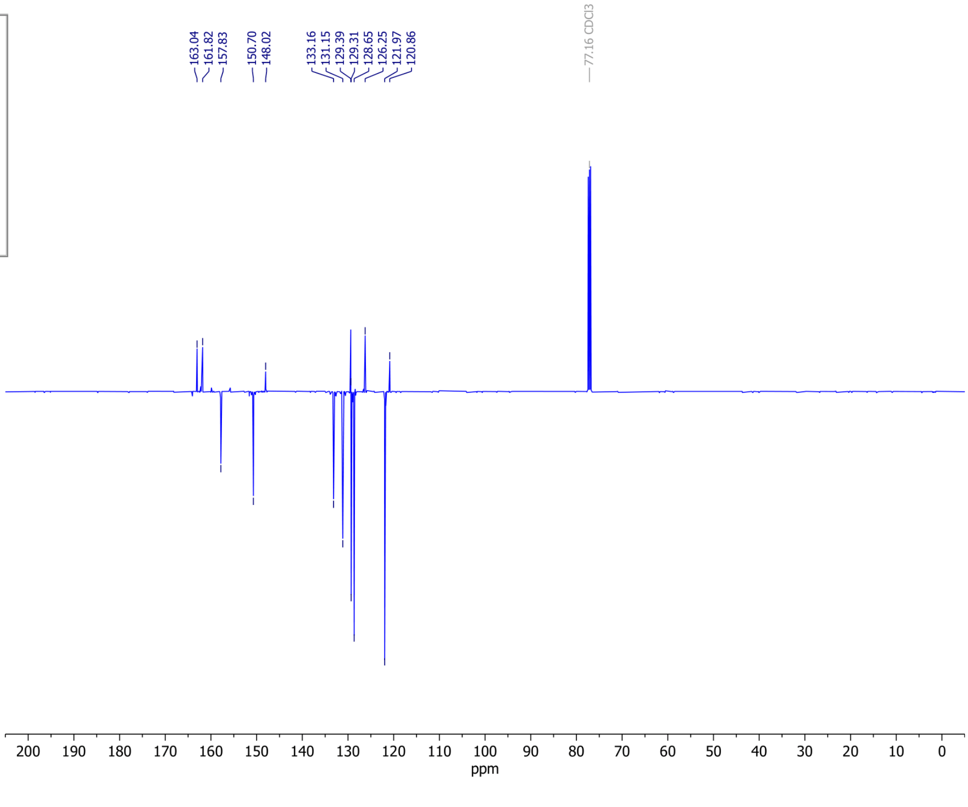

Figure S4. ^{1}H and ^{13}C NMR spectra of compound **9a** in $CDCl_3$.

5-(7-Chlorotetrazolo[1,5-*c*]pyrimidin-8-yl)quinoline (9b)

Parameter	Value
1 Title	Compound 9b (MF-E350)
2 Origin	Bruker BioSpin GmbH
3 Solvent	DMSO
4 Temperature	298.0
5 Number of Scans	16
6 Relaxation Delay	1.0000
7 Acquisition Date	2022-03-05T17:30:23
8 Spectrometer Frequency	499.98
9 Nucleus	1H

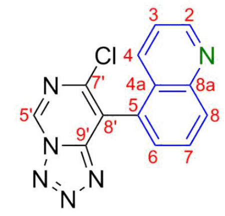

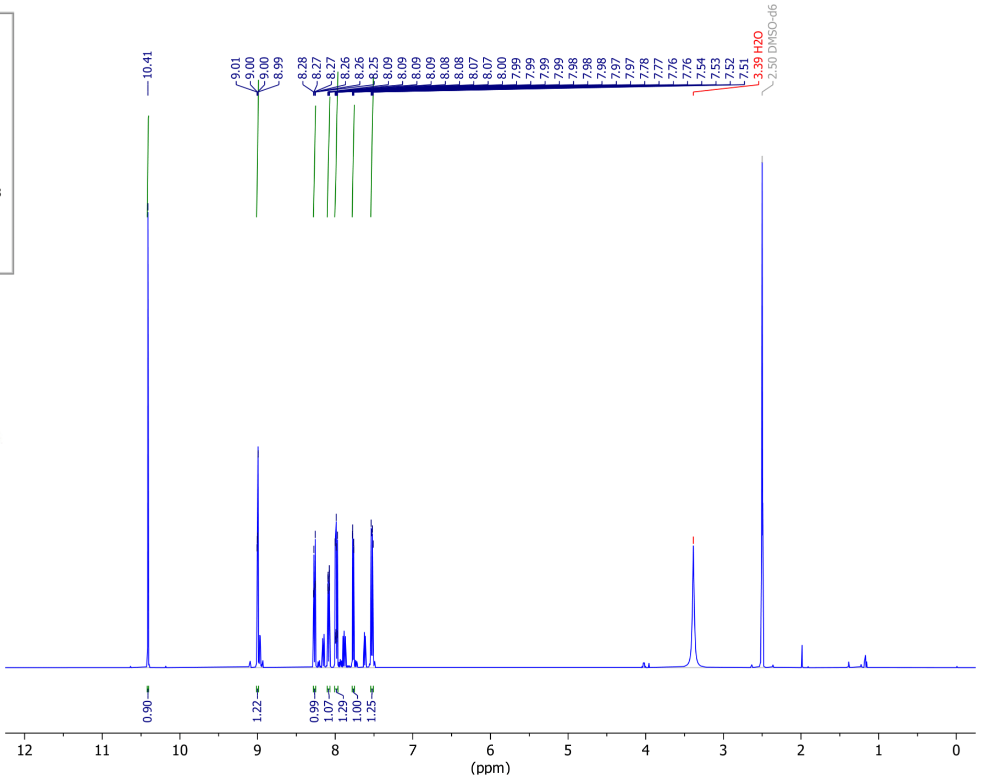

Parameter	Value
1 Title	Compound 9b (MF-E350)
2 Origin	Bruker BioSpin GmbH
3 Solvent	DMSO
4 Temperature	298.0
5 Number of Scans	1024
6 Acquisition Date	2022-03-05T18:24:33
7 Spectrometer Frequency	125.73
8 Nucleus	13C

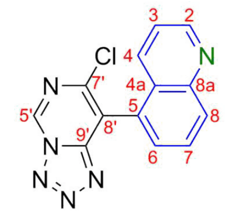

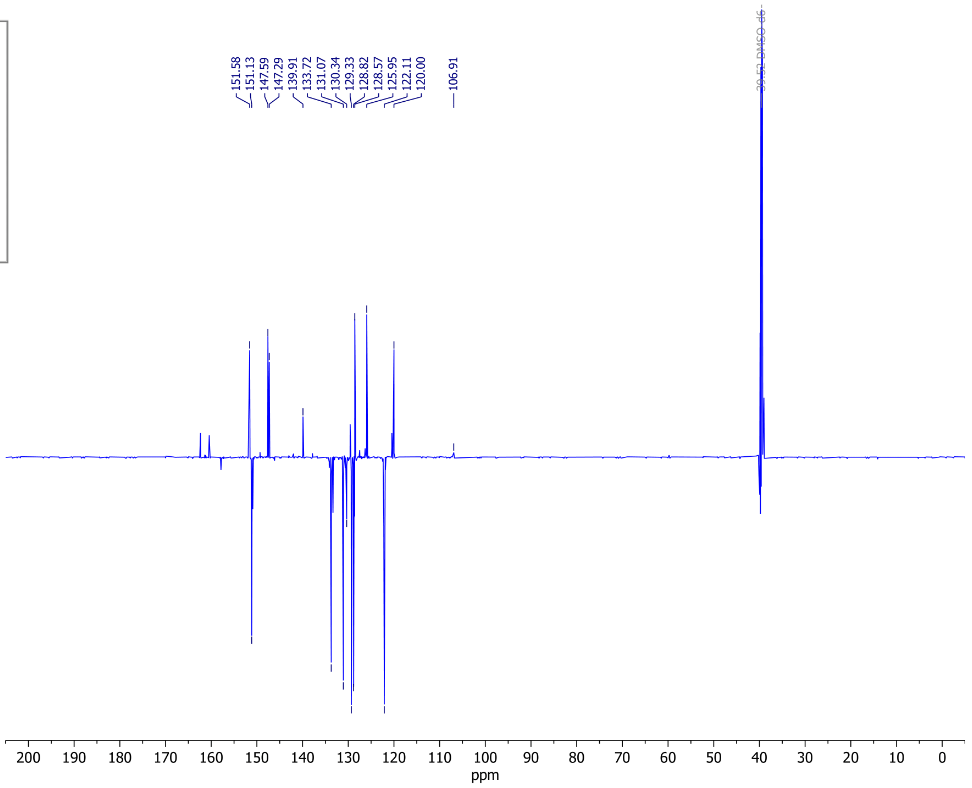

Figure S5. ^{1}H and ^{13}C NMR spectra of compound **9b** in DMSO-d_6.

11-Chloro-7*H*-pyrimido[5',4':4,5]pyrrolo[3,2-*f*]quinoline (10)

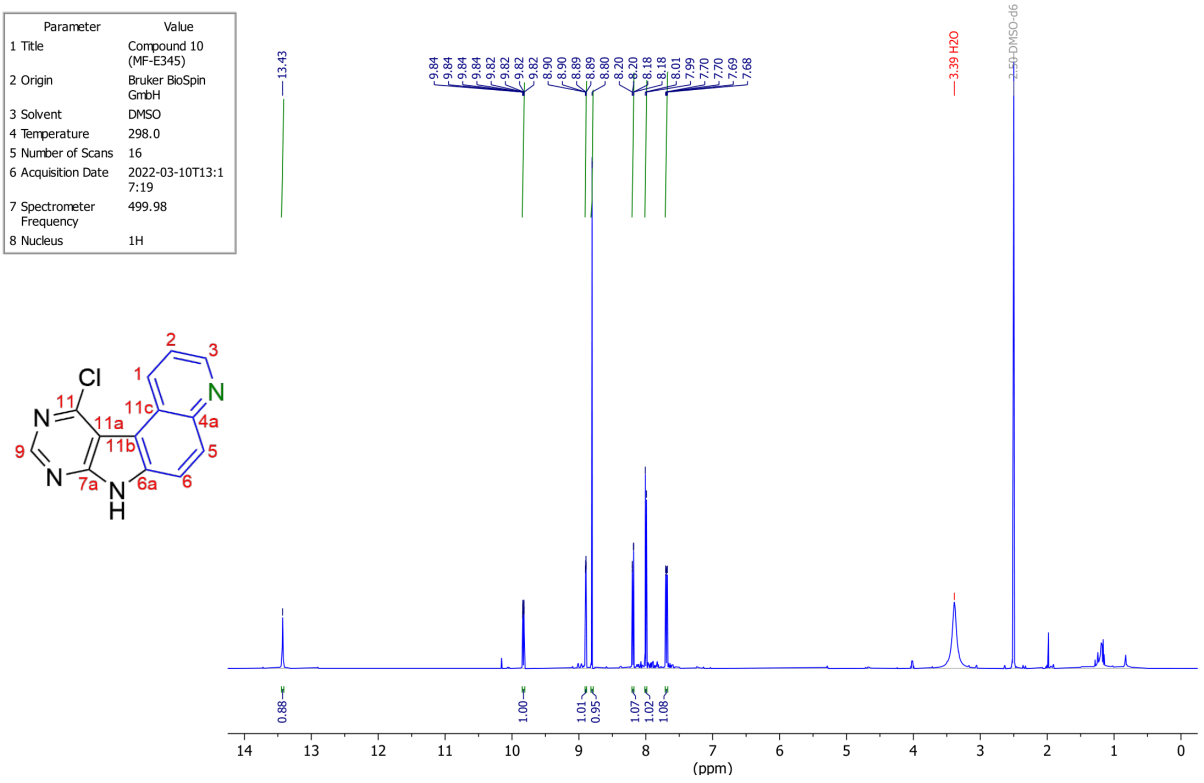

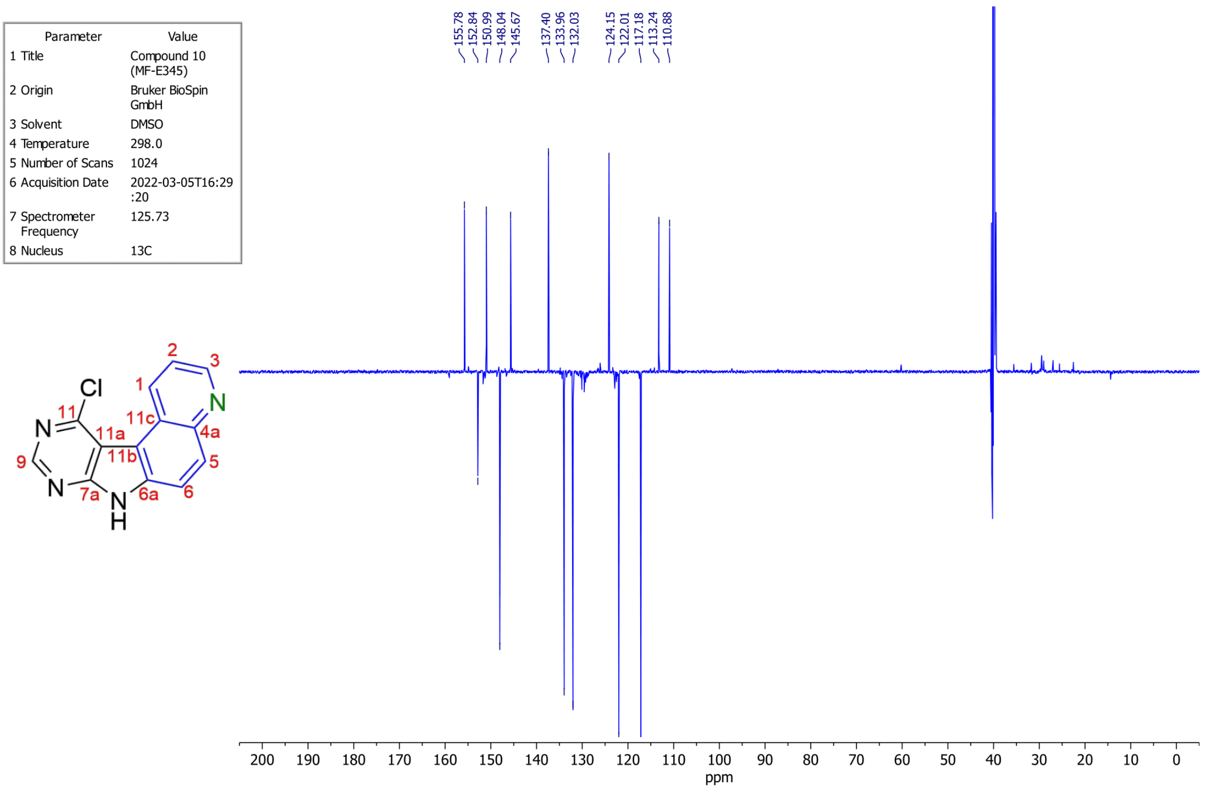

Figure S6. ^{1}H and ^{13}C NMR spectra of compound **10** in DMSO-d_6.

11-Chloro-7-(2,3,5-tri-*O*-benzoyl-*β*-D-ribofuranosyl)pyrimido[5',4':4,5]pyrrolo[3,2-*f*]quinoline (11)

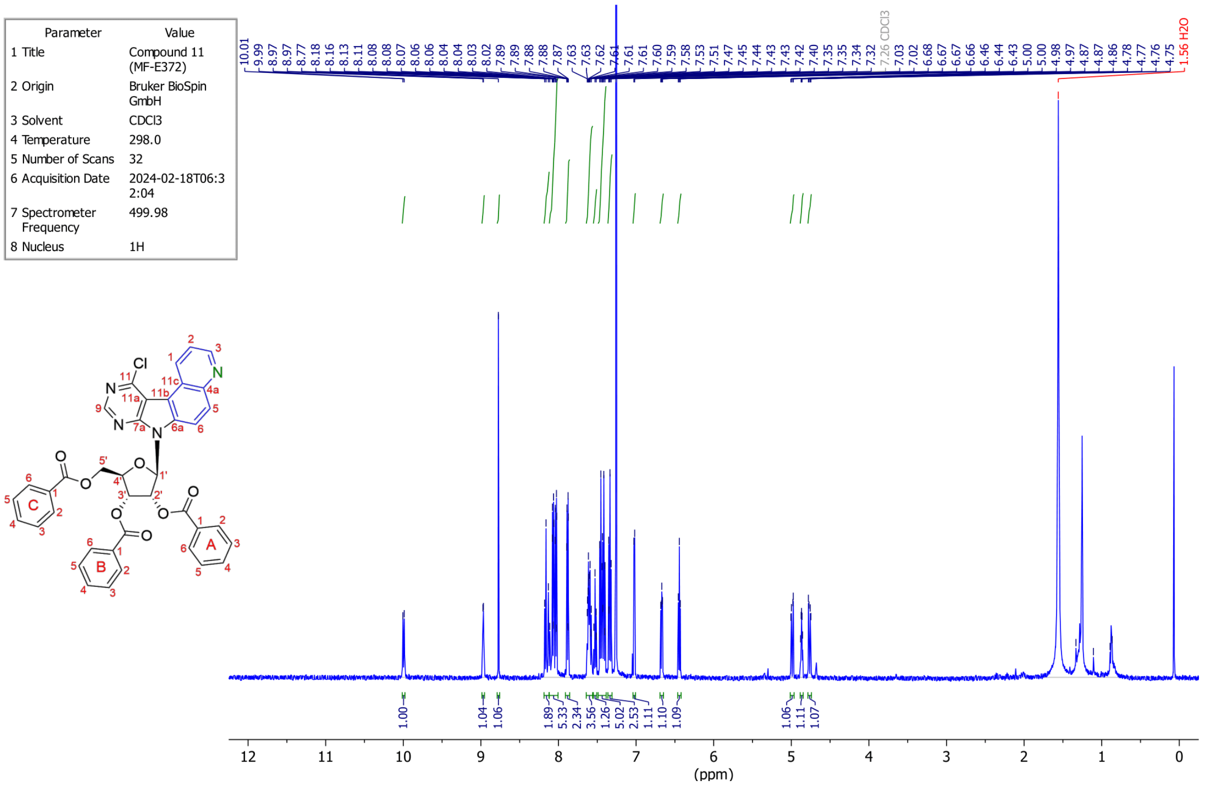

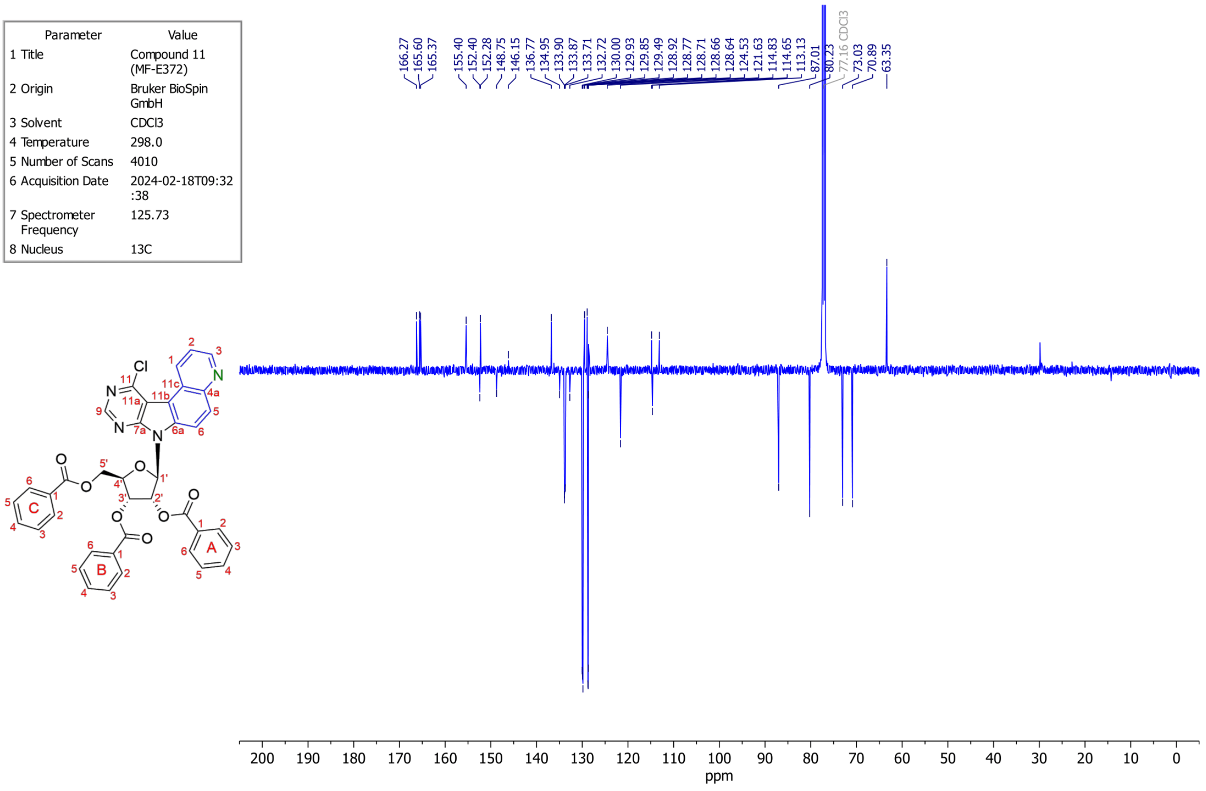

Figure S7. ^{1}H and ^{13}C NMR spectra of compound **11** in $CDCl_3$.

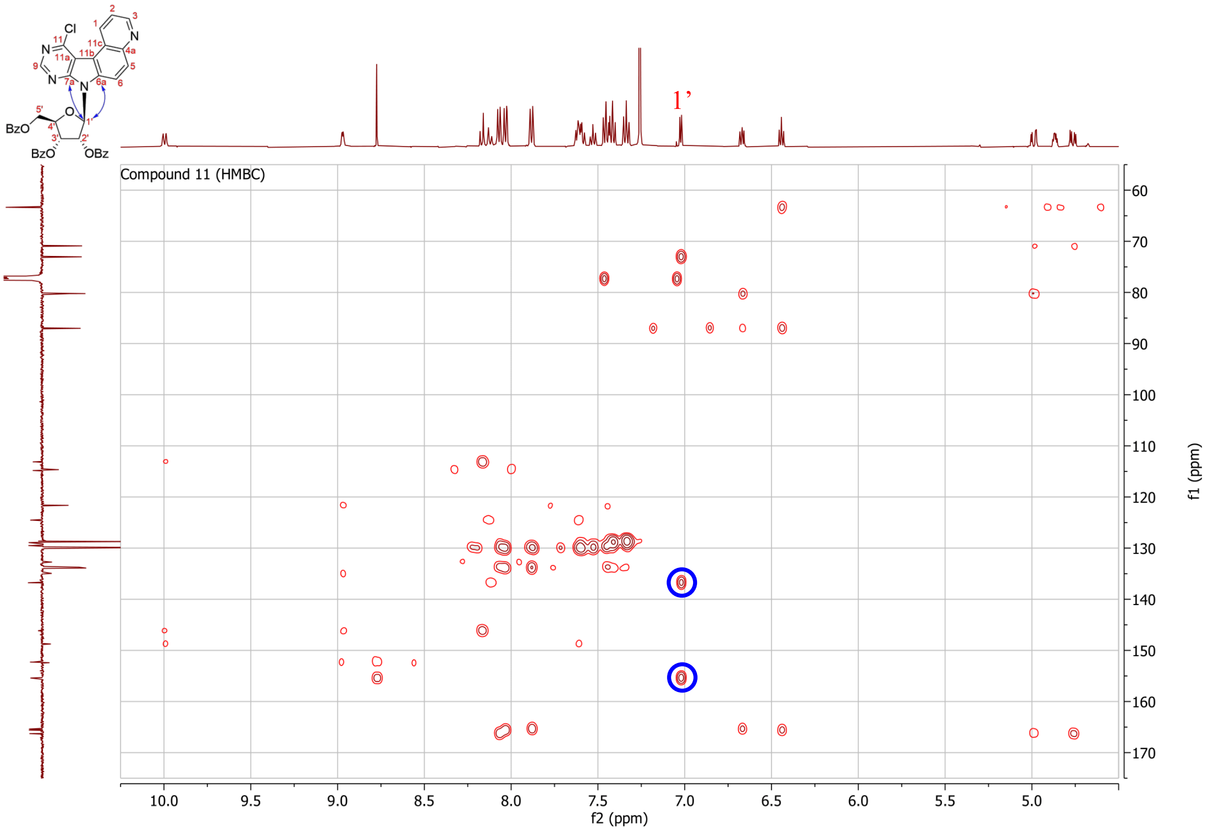

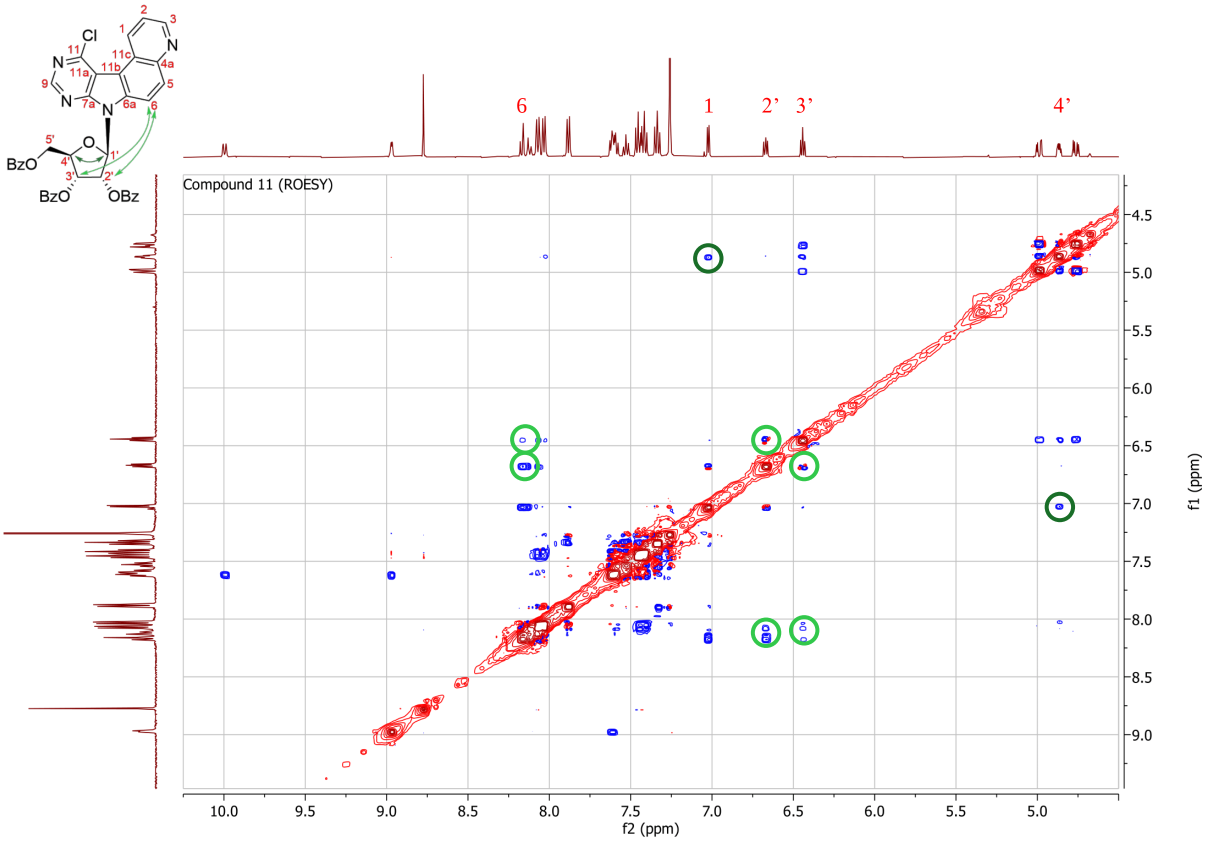

Figure S8. HMBC and ROESY spectra of compound **11** in $CDCl_3$.

11-Chloro-7-(2,3-*O*-isopropylidene-5-*O*-*tert*-butyldimethylsilyl-*β*-D-ribofuranosyl)pyrimido[5',4':4,5]pyrrolo[3,2-*f*]quinoline (12-*β*)

Parameter	Value
1 Title	Compound 12beta (MF-G523beta)
2 Origin	Bruker BioSpin GmbH
3 Solvent	CDCl3
4 Temperature	298.0
5 Number of Scans	32
6 Acquisition Date	2024-02-18T15:19:01
7 Spectrometer Frequency	499.98
8 Nucleus	1H

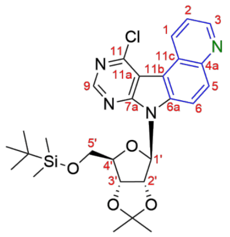

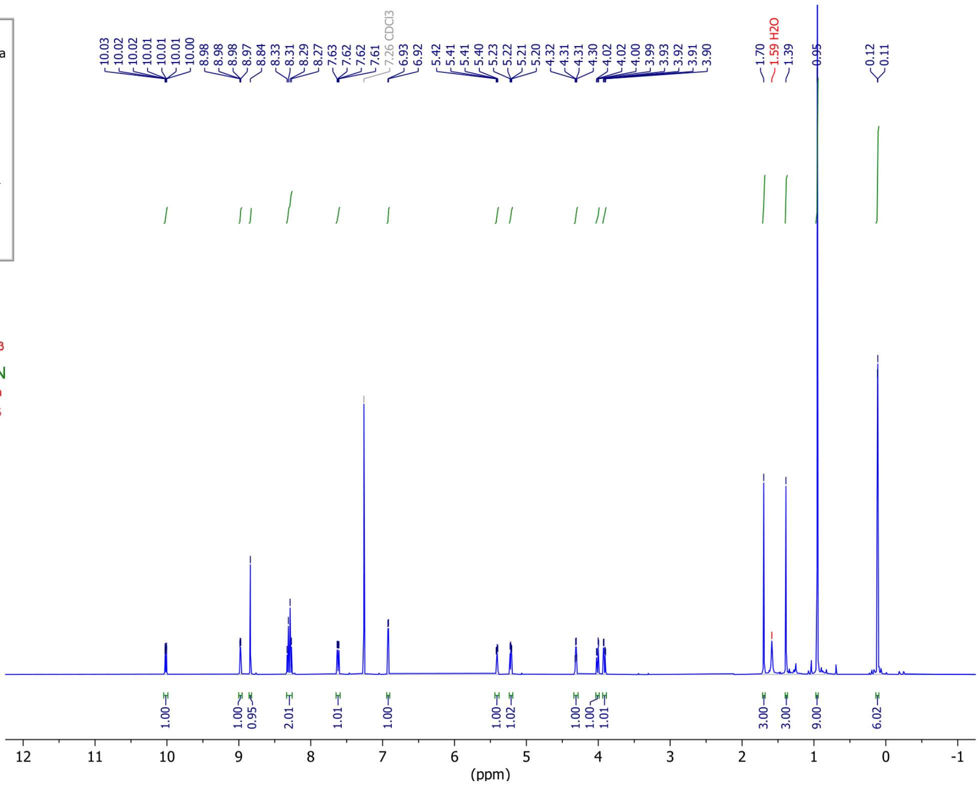

Parameter	Value
1 Title	Compound 12beta (MF-G523beta)
2 Origin	Bruker BioSpin GmbH
3 Solvent	CDCl3
4 Temperature	298.0
5 Number of Scans	2048
6 Acquisition Date	2024-02-18T17:06:53
7 Spectrometer Frequency	125.73
8 Nucleus	13C

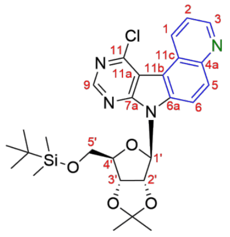

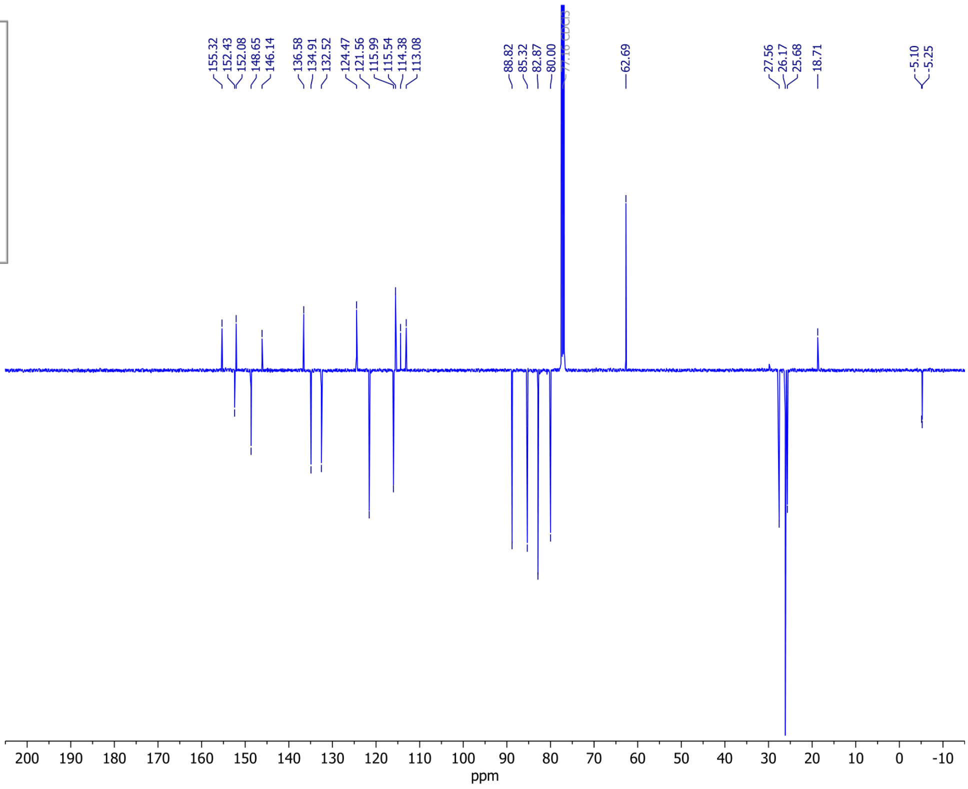

Figure S9. ^{1}H and ^{13}C NMR spectra of compound **12-*β*** in $CDCl_3$.

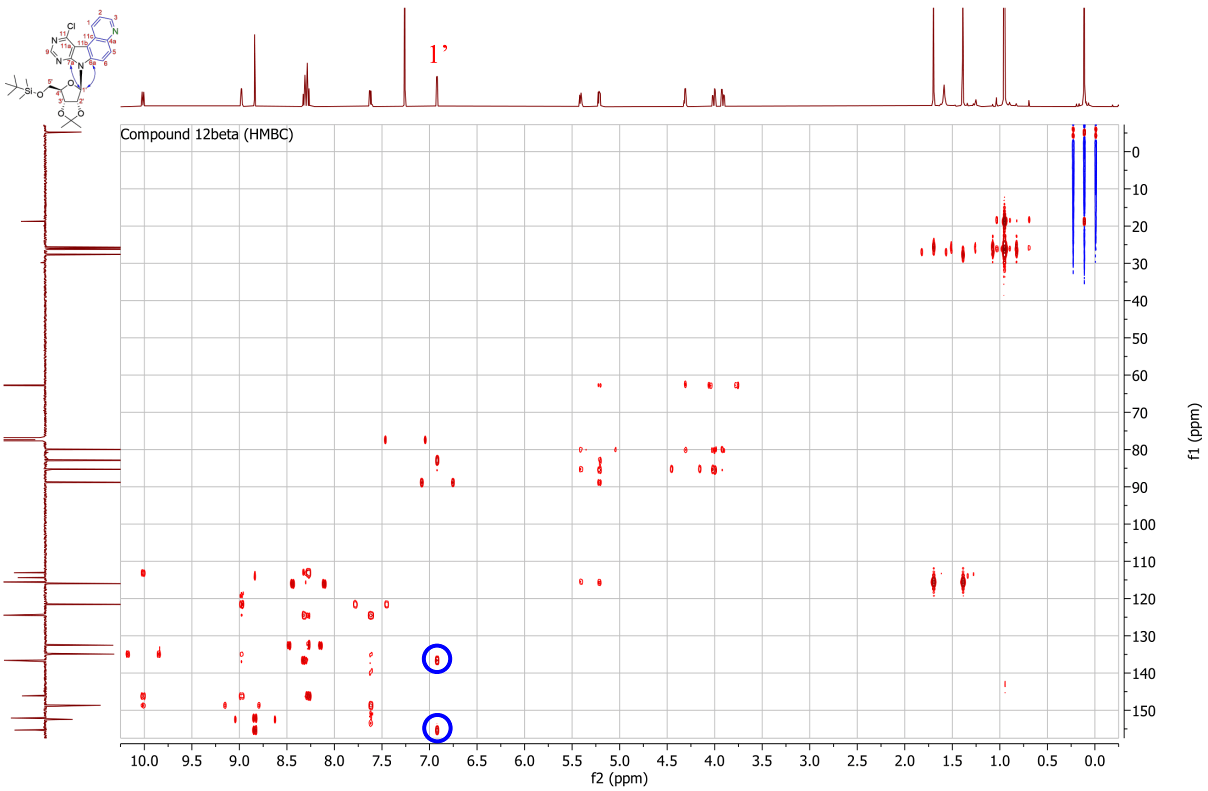

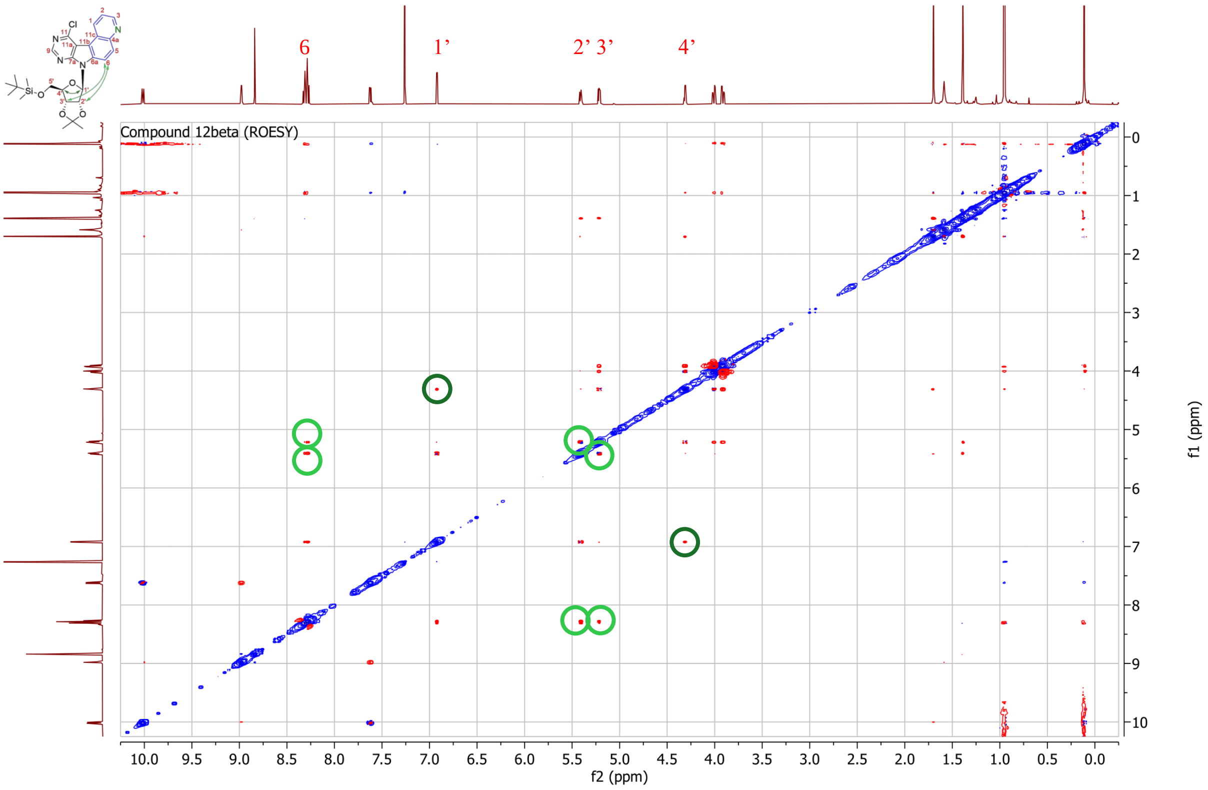

Figure S10. HMBC and ROESY spectra of compound **12-*β*** in $CDCl_3$.

11-Chloro-7-(2,3-*O*-isopropylidene-5-*O*-*tert*-butyldimethylsilyl-*α*-D-ribofuranosyl)pyrimido[5',4':4,5]pyrrolo[3,2-*f*]quinoline (12-*α*)

Parameter	Value
1 Title	Compound 12alpha (MF-G523alpha)
2 Origin	Bruker BioSpin GmbH
3 Solvent	CDCl3
4 Temperature	298.0
5 Number of Scans	16
6 Acquisition Date	2024-02-27T16:39:00
7 Spectrometer Frequency	499.98
8 Nucleus	1H

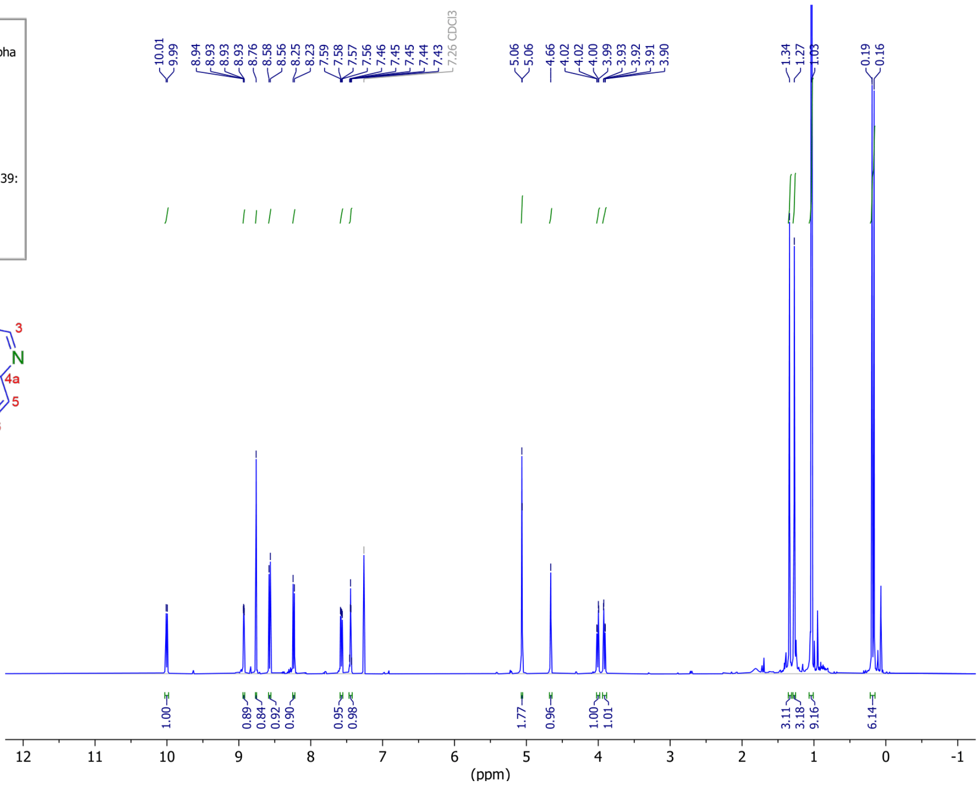

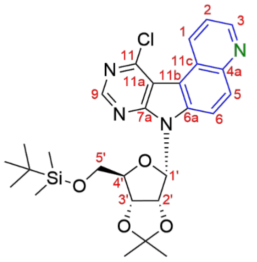

Parameter	Value
1 Title	Compound 12alpha (MF-G523alpha)
2 Origin	Bruker BioSpin GmbH
3 Solvent	CDCl3
4 Temperature	298.0
5 Number of Scans	3072
6 Acquisition Date	2024-02-27T19:22:00
7 Spectrometer Frequency	125.73
8 Nucleus	13C

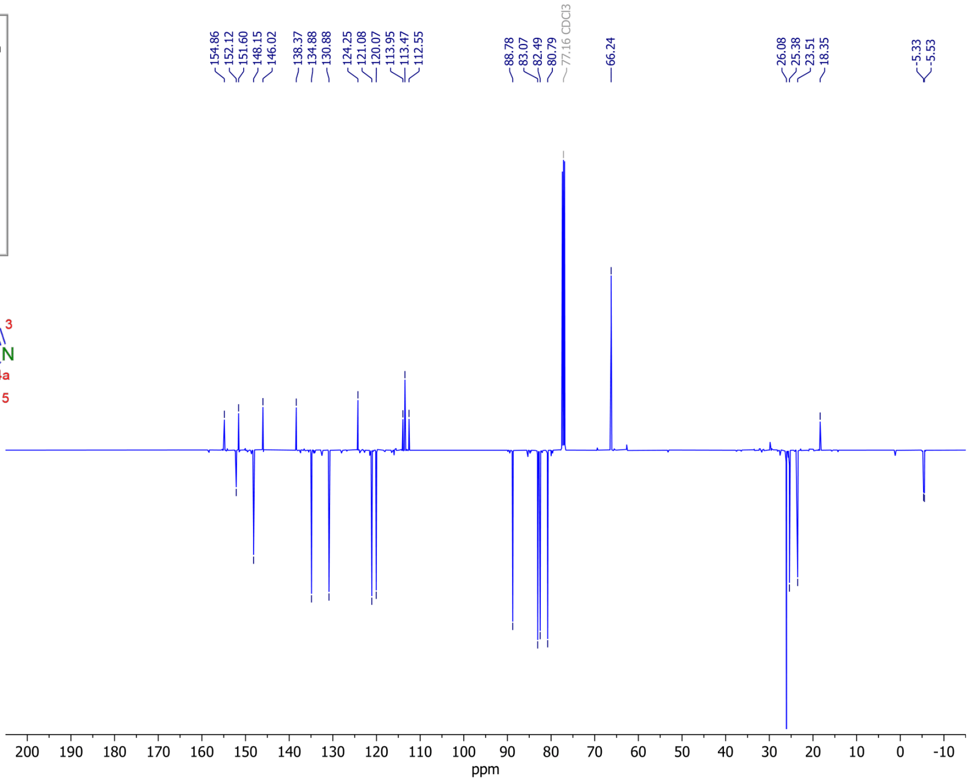

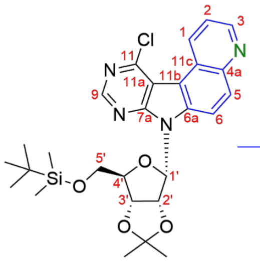

Figure S11. ^{1}H and ^{13}C NMR spectra of compound **12-*α*** in $CDCl_3$.

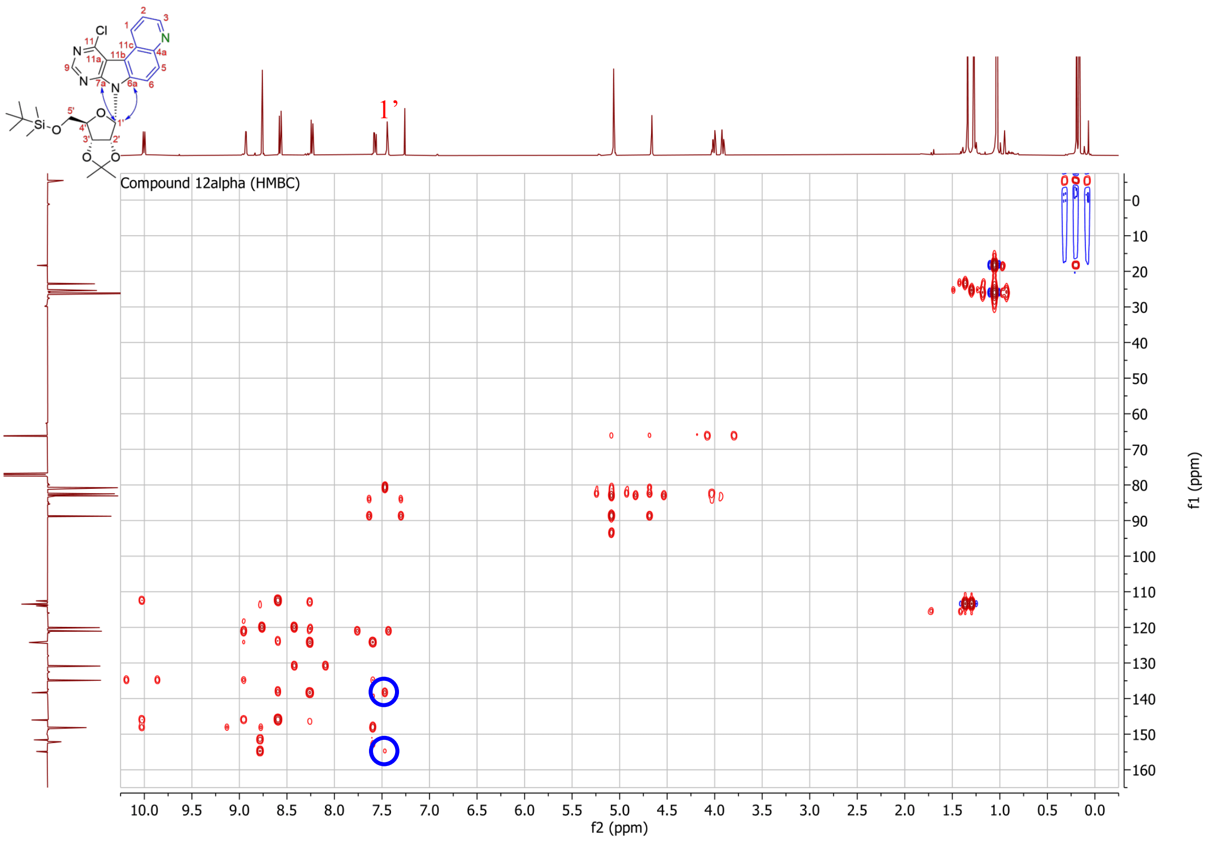

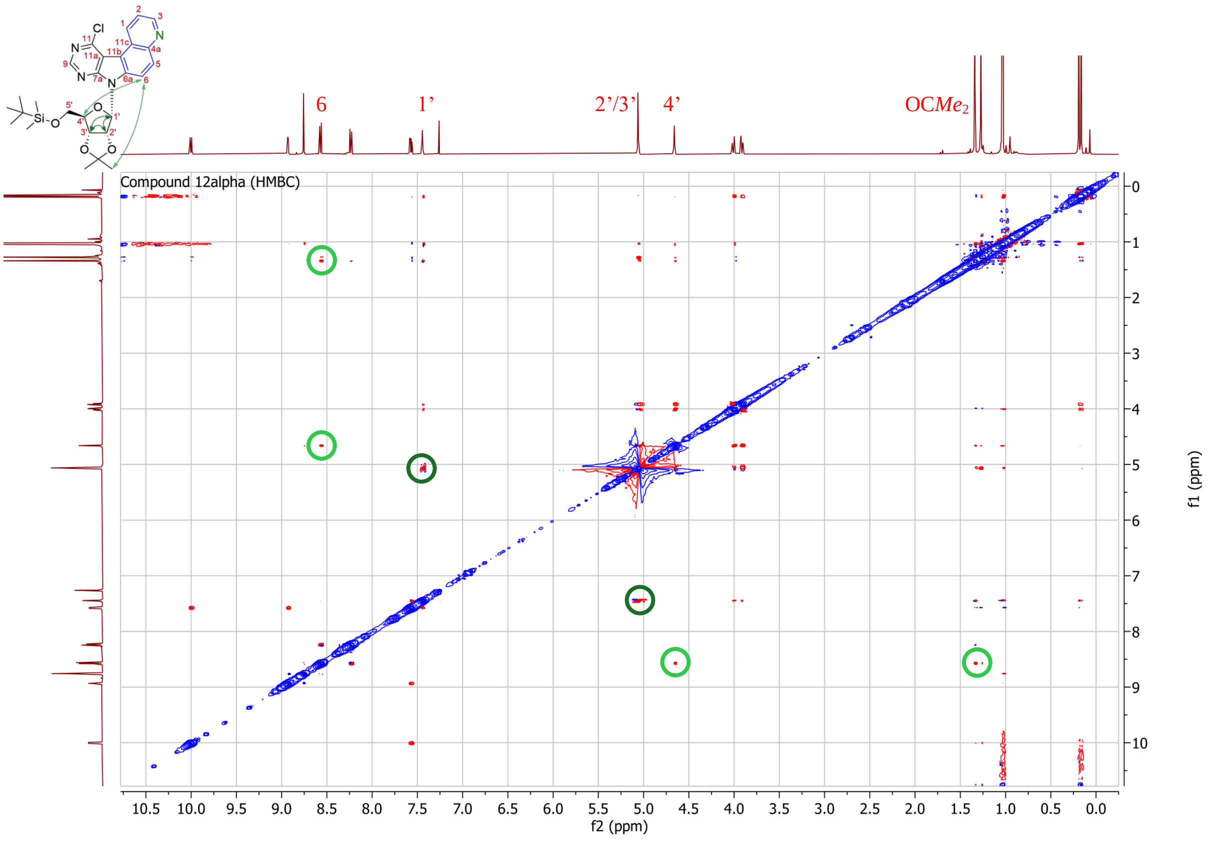

Figure S12. HMBC and ROESY spectra of compound **12-*α*** in $CDCl_3$.

11-(Furan-2-yl)-7-(2,3,5-tri-*O*-benzoyl-*β*-D-ribofuranosyl)pyrimido[5',4':4,5]pyrrolo[3,2-*f*]quinoline (13a)

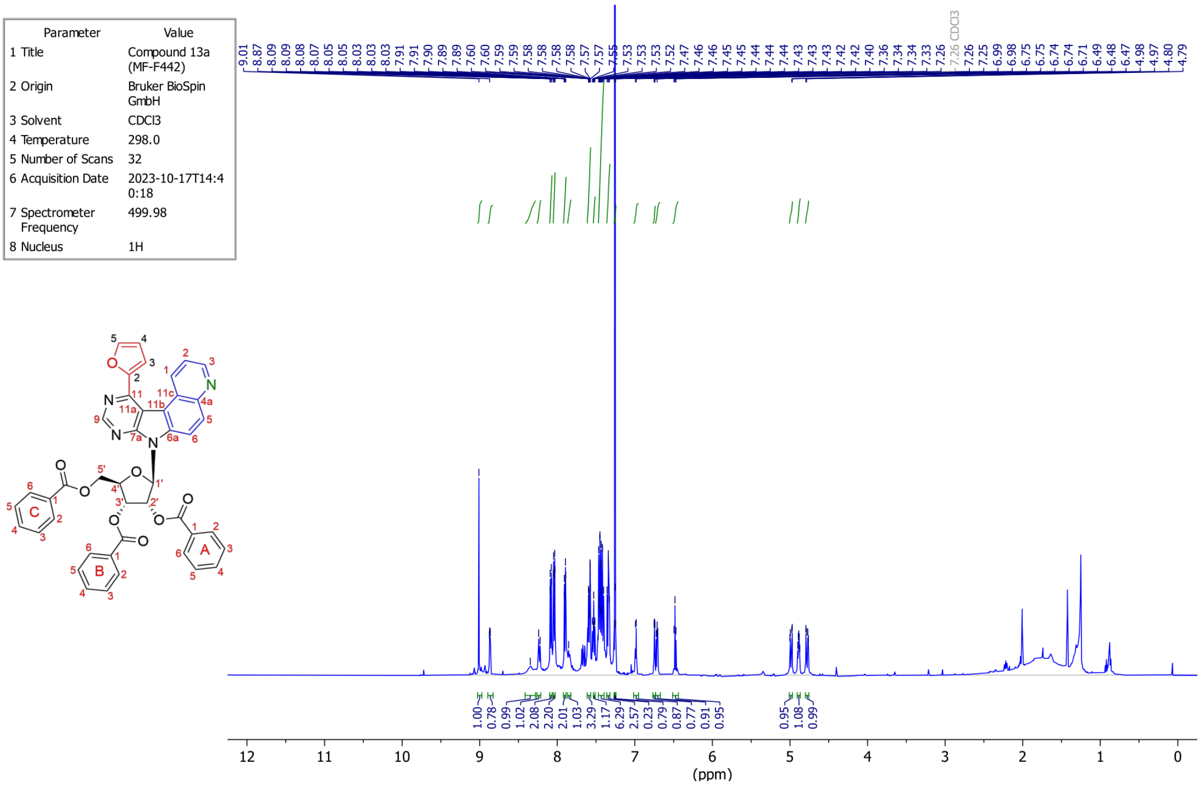

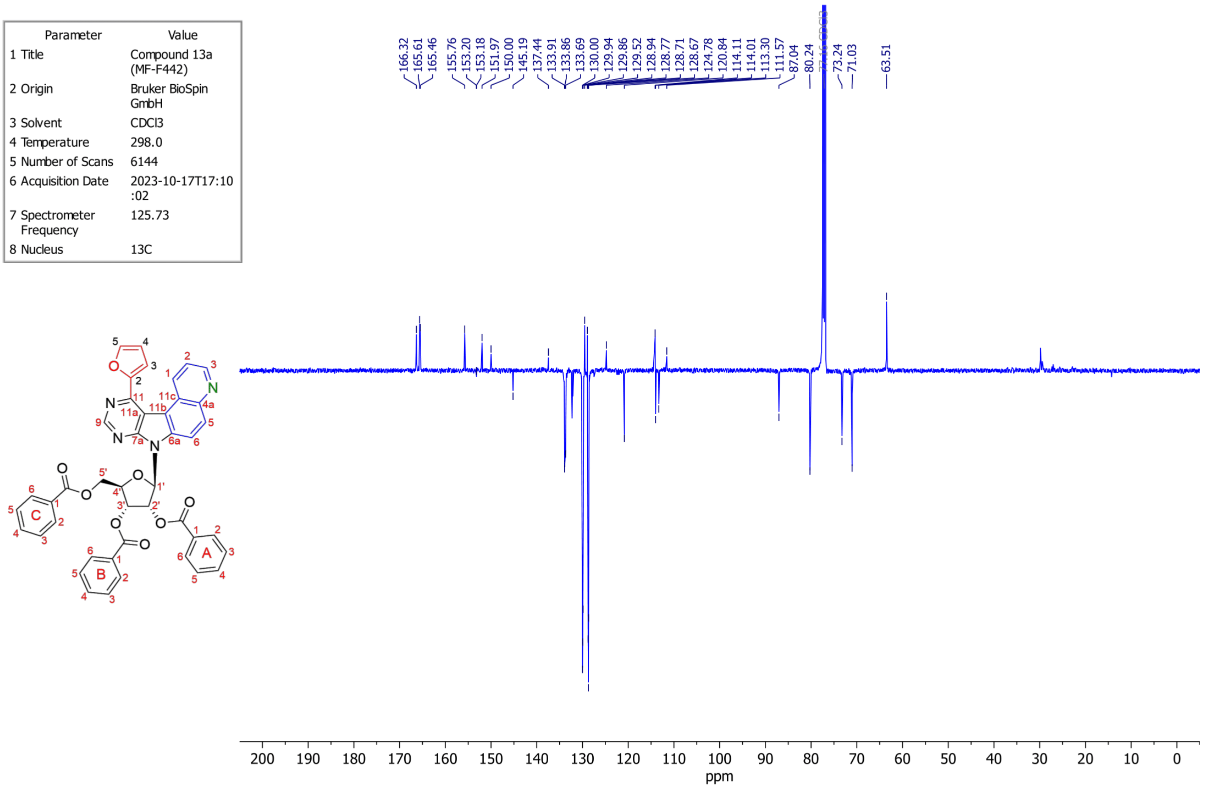

Figure S13. ^{1}H and ^{13}C NMR spectra of compound **13a** in $CDCl_3$.

11-(Benzofuran-2-yl)-7-(2,3,5-tri-*O*-benzoyl-*β*-D-ribofuranosyl)pyrimido[5',4':4,5]pyrrolo[3,2-*f*]quinoline (13b)

Parameter	Value
1 Title	Compound 13b (MF-F466)
2 Origin	Bruker BioSpin GmbH
3 Solvent	CDCl3
4 Temperature	298.0
5 Number of Scans	32
6 Acquisition Date	2024-02-26T23:54:00
7 Spectrometer Frequency	499.98
8 Nucleus	1H

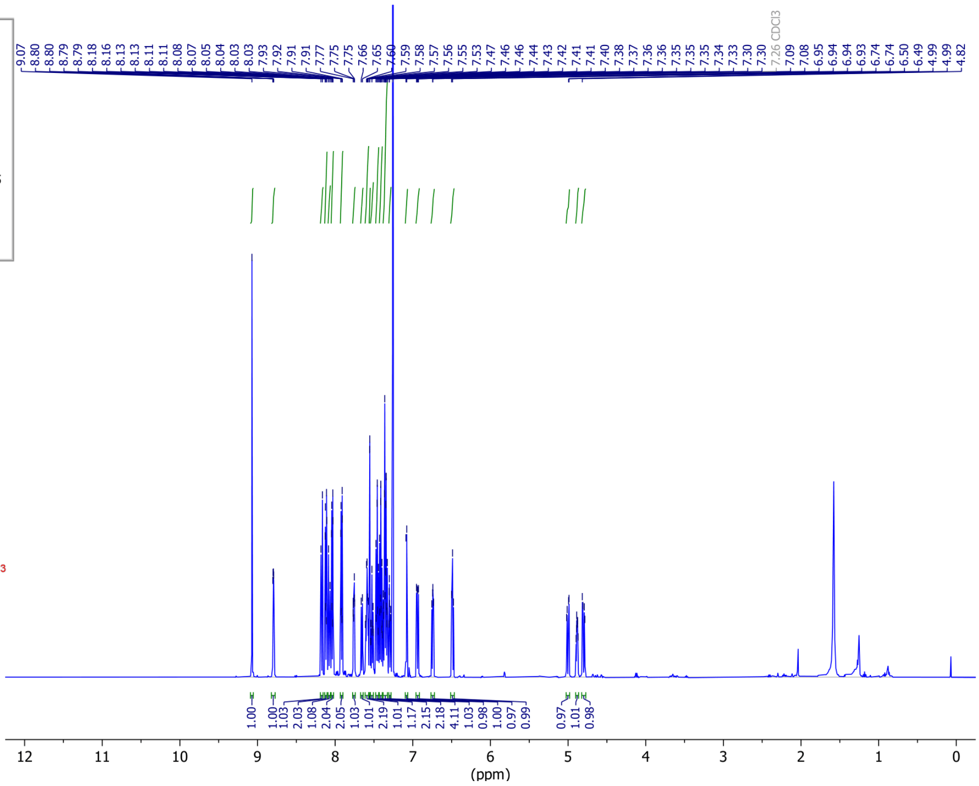

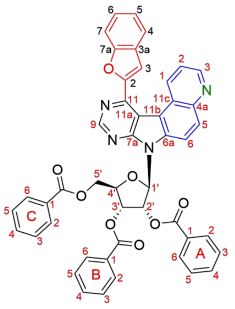

Parameter	Value
1 Title	Compound 13b (MF-F466)
2 Origin	Bruker BioSpin GmbH
3 Solvent	CDCl3
4 Temperature	298.0
5 Number of Scans	3072
6 Acquisition Date	2024-02-27T02:36:00
7 Spectrometer Frequency	125.73
8 Nucleus	13C

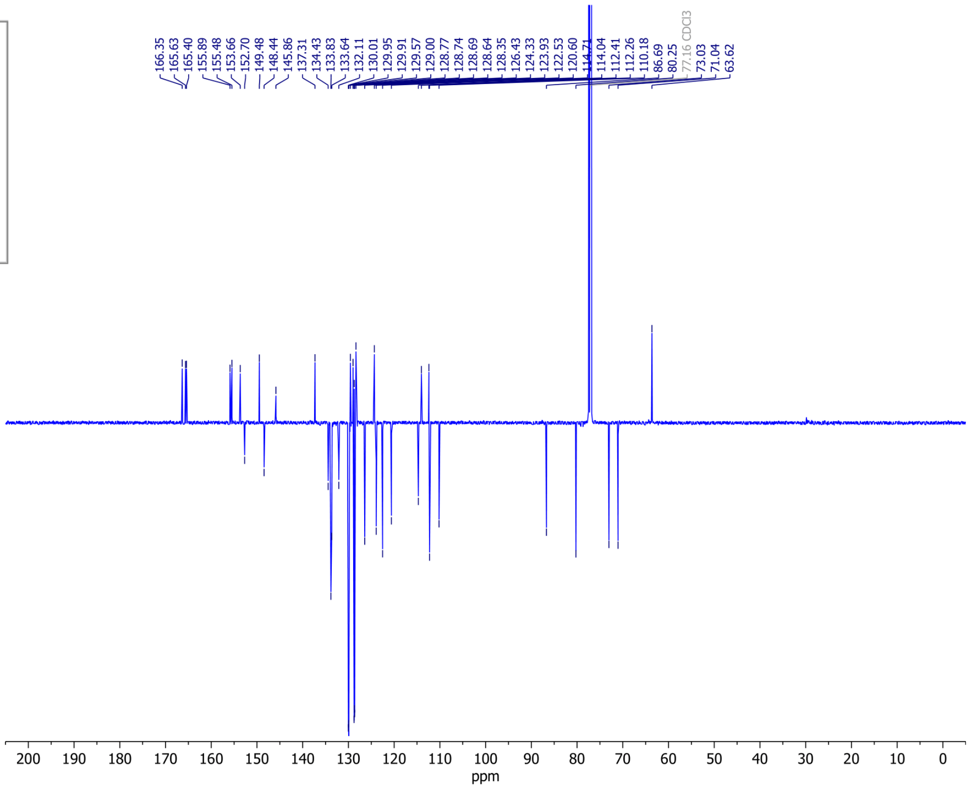

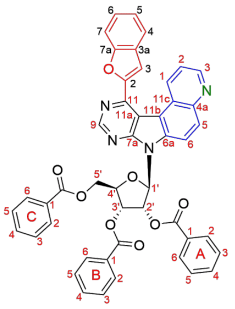

Figure S14. ^{1}H and ^{13}C NMR spectra of compound **13b** in $CDCl_3$.

Methyl-7-(2,3,5-tri-*O*-benzoyl-*β*-D-ribofuranosyl)pyrimido[5',4':4,5]pyrrolo[3,2-*f*]quinoline (13c)

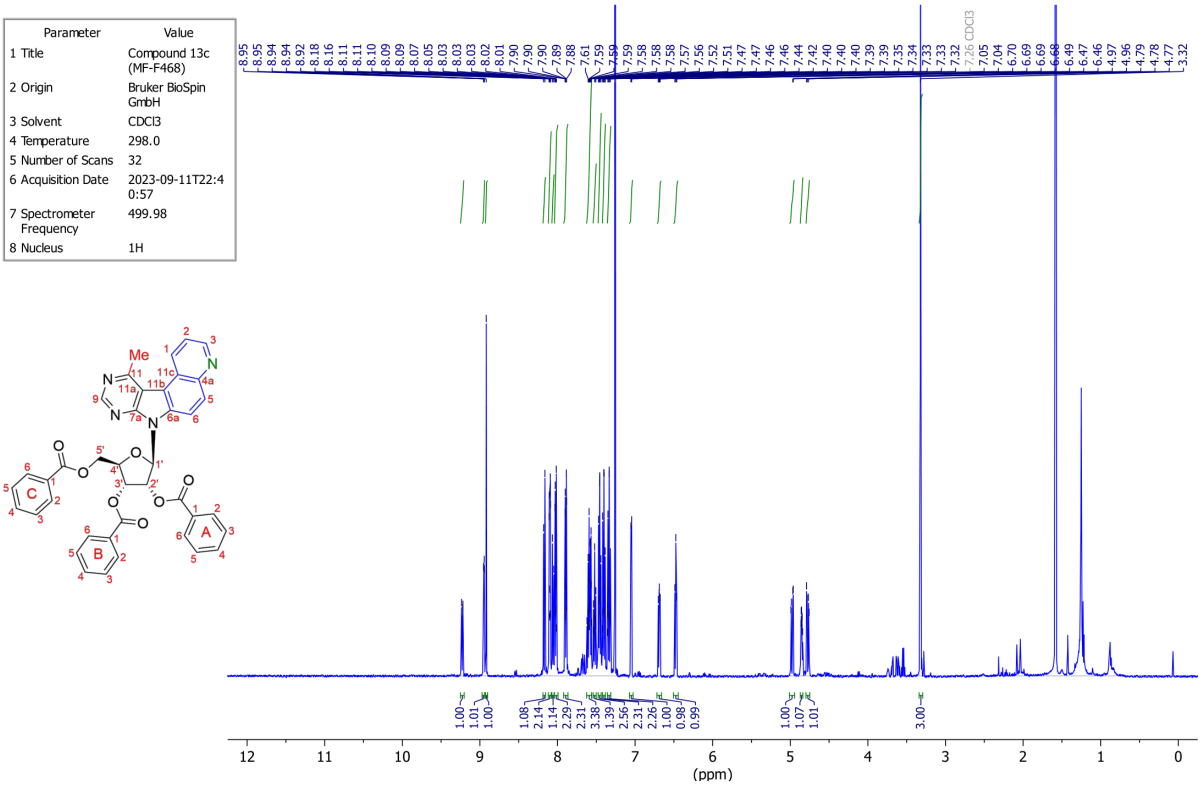

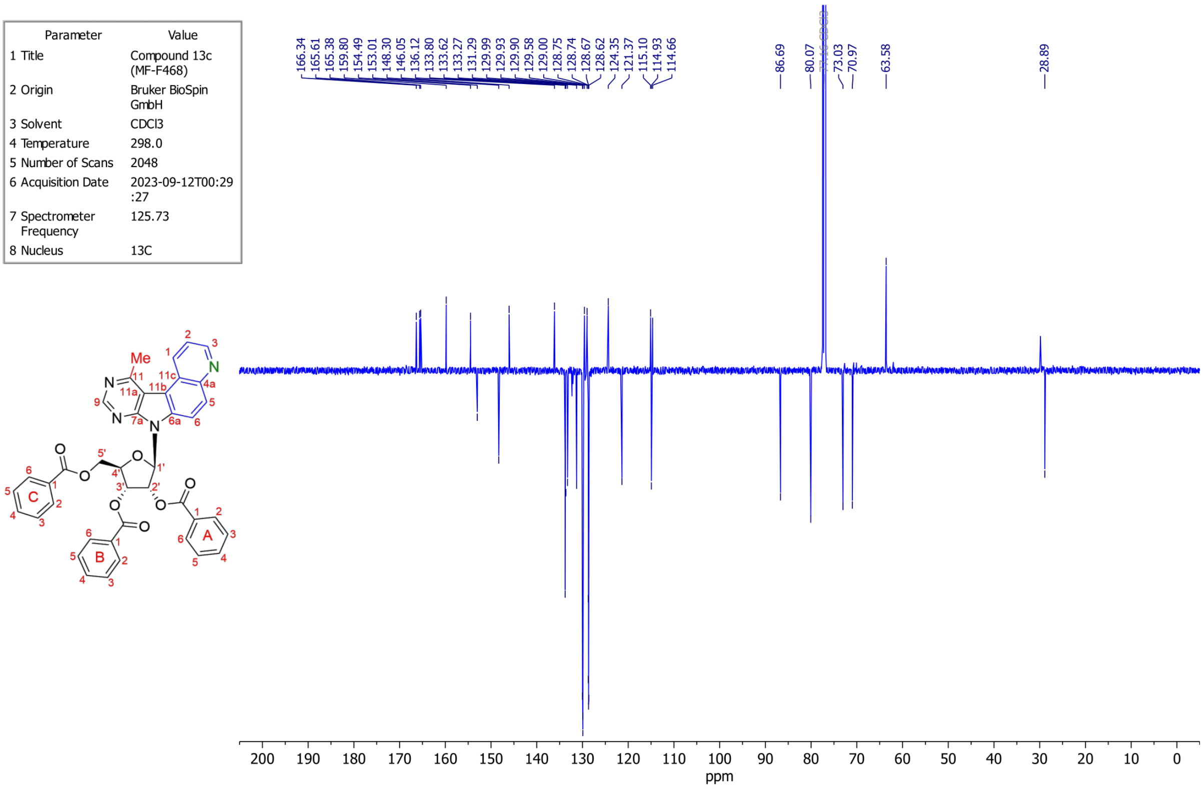

Figure S15. ^{1}H and ^{13}C NMR spectra of compound **13c** in $CDCl_3$.

11-(*N*,*N*-dimethylamino)-7-(2,3,5-tri-*O*-benzoyl-*β*-D-ribofuranosyl)pyrimido[5',4':4,5]pyrrolo[3,2-*f*]quinoline (13d)

Parameter	Value
1 Title	Compound 13d (MF-G474)
2 Origin	Bruker BioSpin GmbH
3 Solvent	CDCl3
4 Temperature	298.0
5 Number of Scans	32
6 Acquisition Date	2024-02-26T19:59:00
7 Spectrometer Frequency	499.98
8 Nucleus	1H

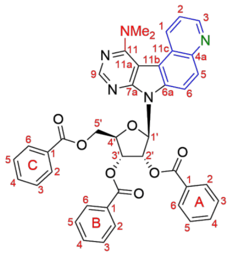

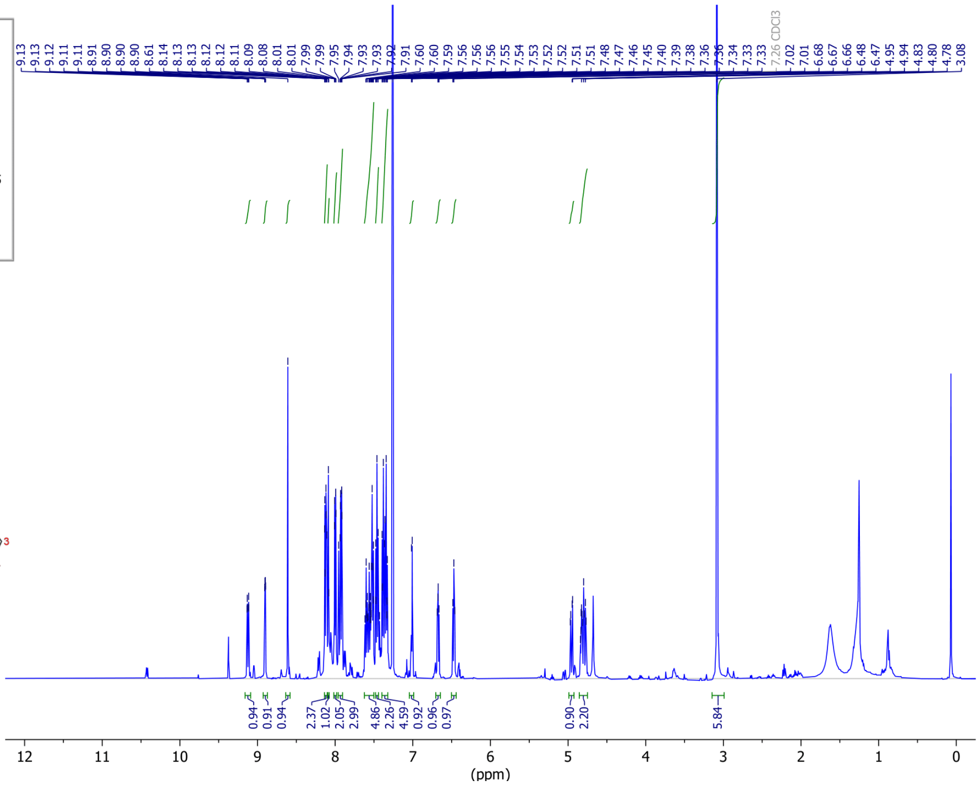

Parameter	Value
1 Title	Compound 13d (MF-G474)
2 Origin	Bruker BioSpin GmbH
3 Solvent	CDCl3
4 Temperature	298.0
5 Number of Scans	3072
6 Acquisition Date	2024-02-26T22:41:00
7 Spectrometer Frequency	125.73
8 Nucleus	13C

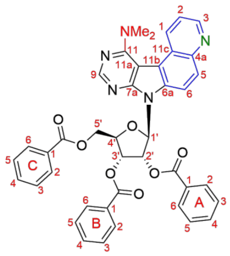

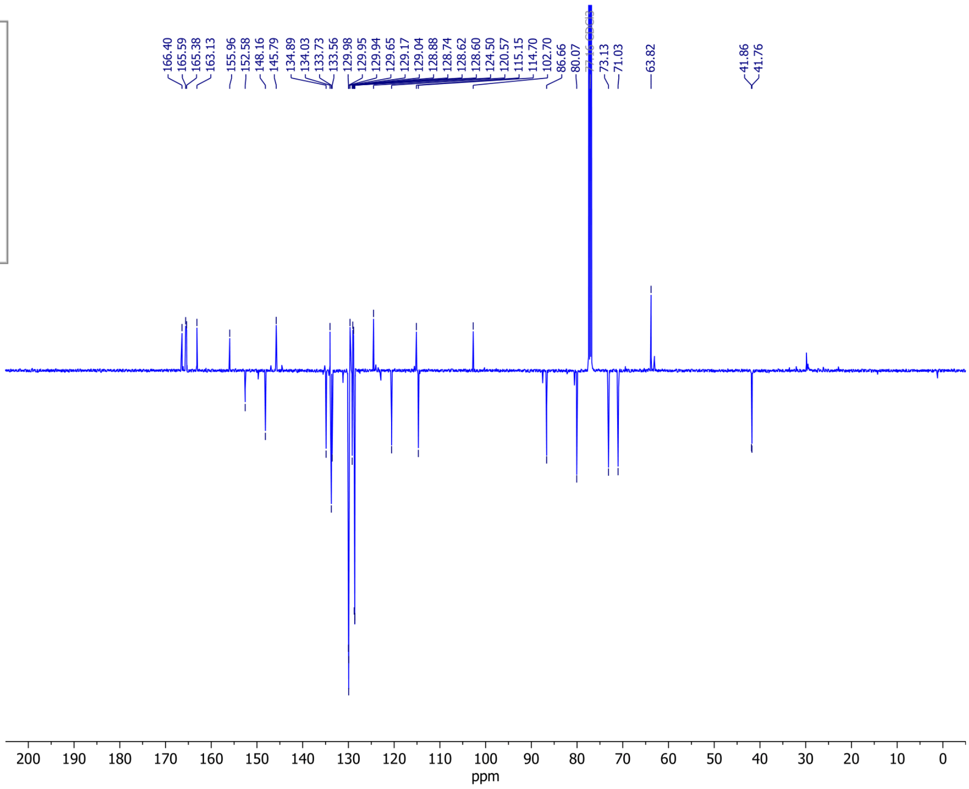

Figure S16. ^{1}H and ^{13}C NMR spectra of compound **13d** in $CDCl_3$.

11-(Furan-2-yl)-7-(*β*-D-ribofuranosyl)pyrimido[5',4':4,5]pyrrolo[3,2-*f*]quinoline (14a)

Parameter	Value
1 Title	Compound 14a (MF-G479)
2 Origin	Bruker BioSpin GmbH
3 Solvent	DMSO
4 Temperature	298.0
5 Number of Scans	32
6 Acquisition Date	2023-12-12T00:35:00
7 Spectrometer Frequency	499.98
8 Nucleus	1H

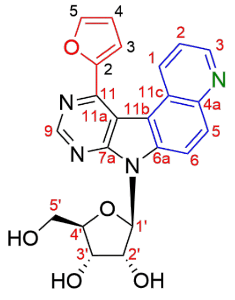

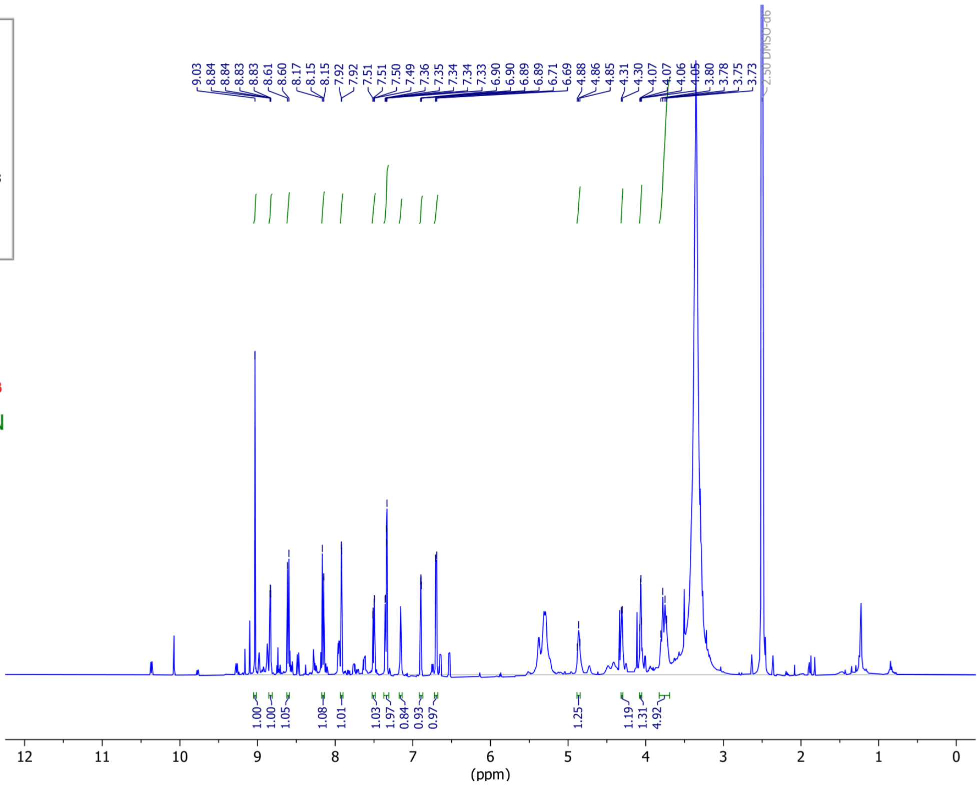

Parameter	Value
1 Title	Compound 14a (MF-G479)
2 Origin	Bruker BioSpin GmbH
3 Solvent	DMSO
4 Temperature	298.0
5 Number of Scans	2048
6 Acquisition Date	2023-12-12T02:23:00
7 Spectrometer Frequency	125.73
8 Nucleus	13C

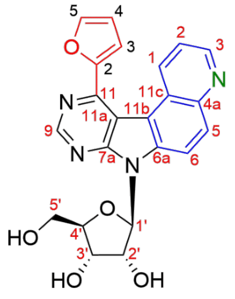

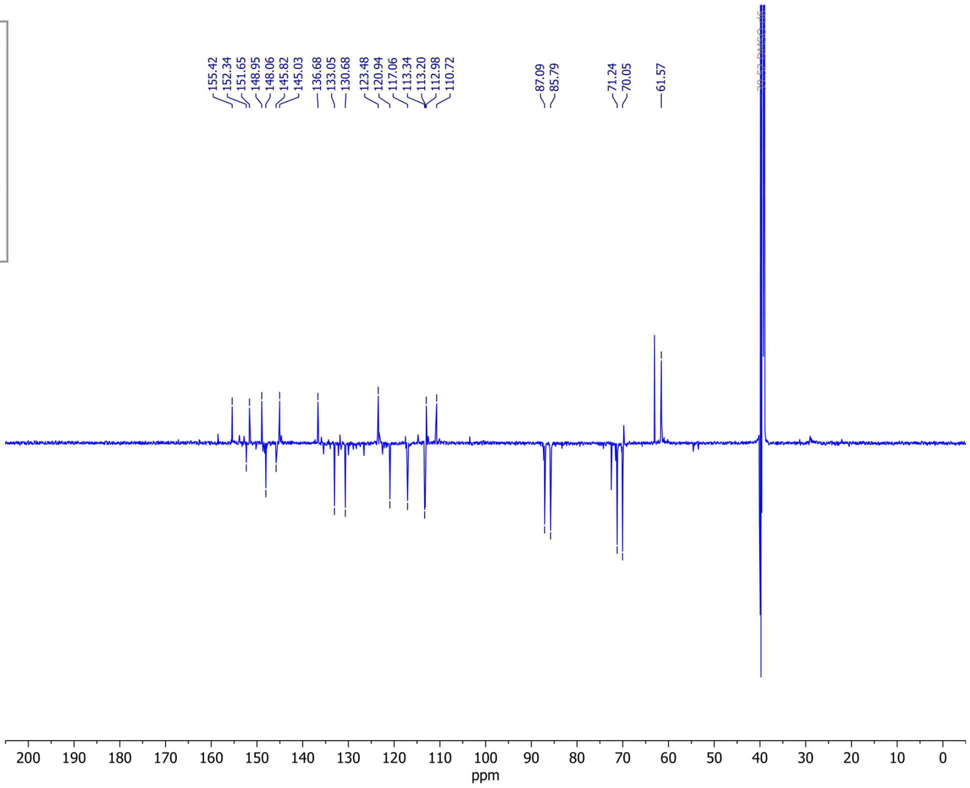

Figure S17. ^{1}H and ^{13}C NMR spectra of compound **14a** in DMSO-d_6.

11-(Benzofuran-2-yl)-7-(*β*-D-ribofuranosyl)pyrimido[5',4':4,5]pyrrolo[3,2-*f*]quinoline (14b)

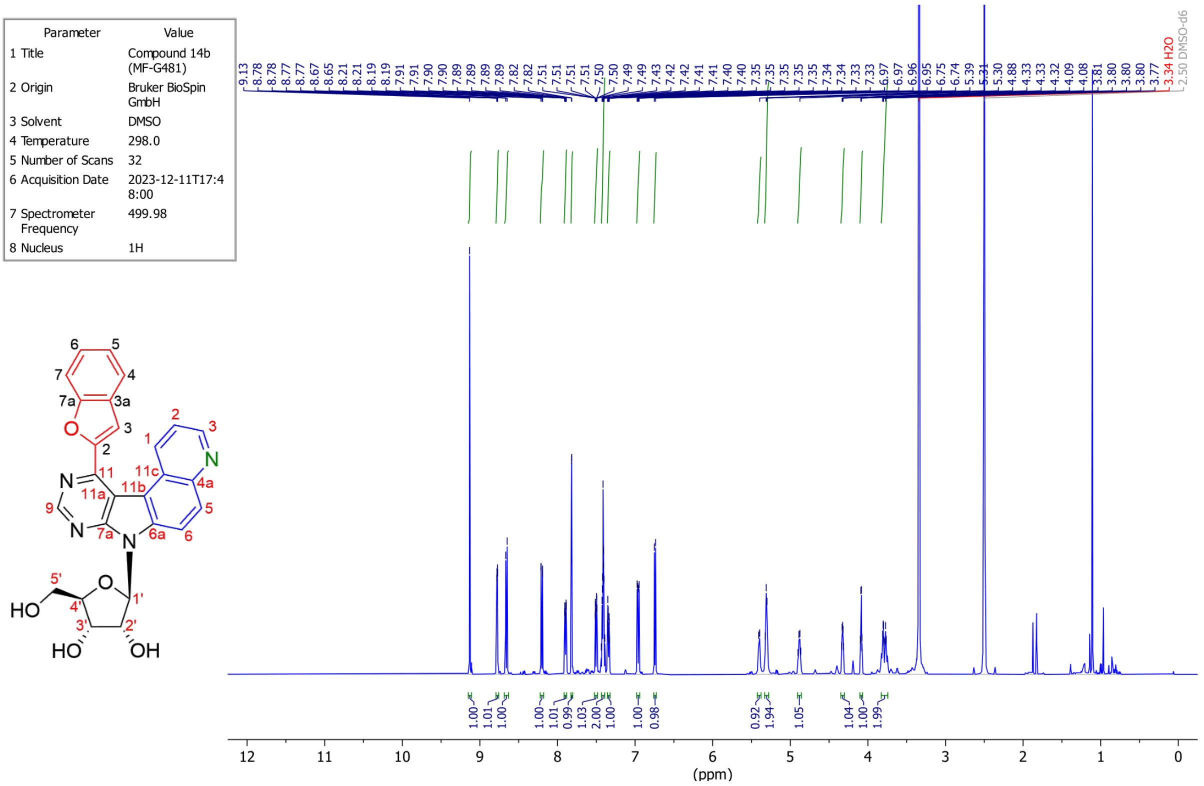

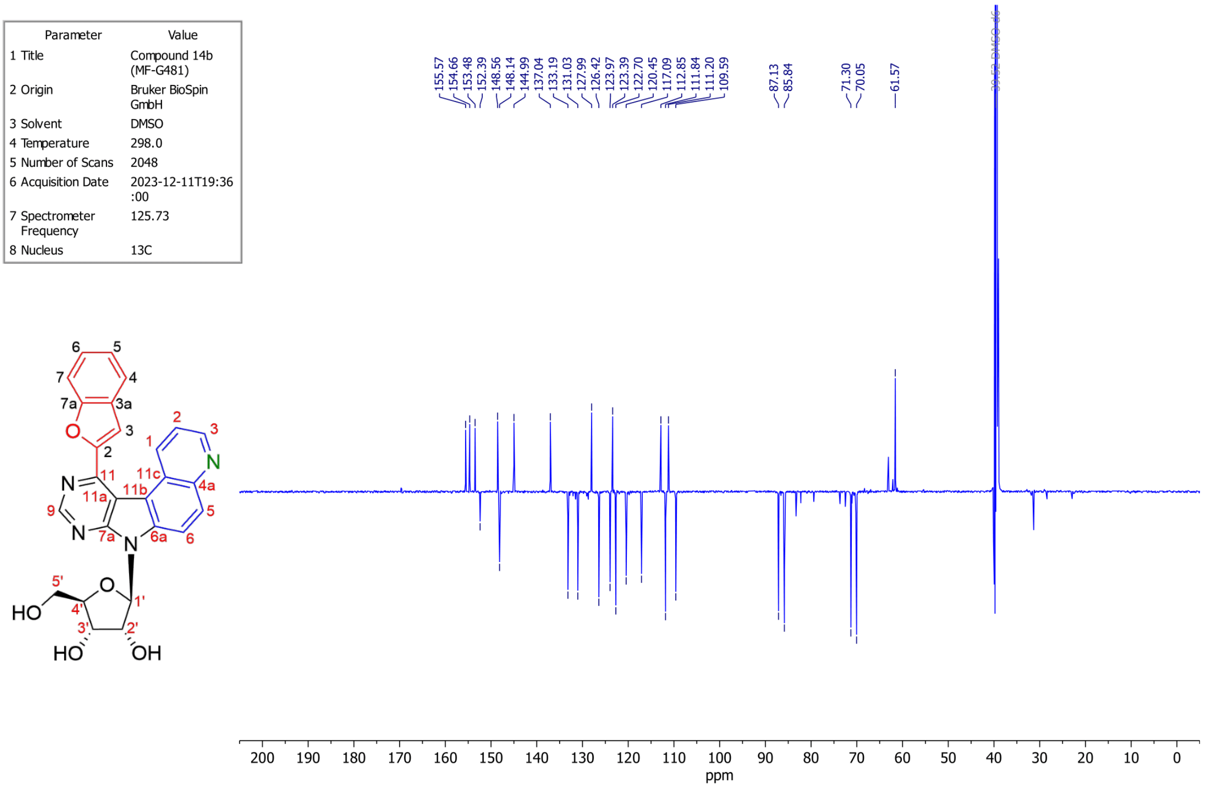

Figure S18. ^{1}H and ^{13}C NMR spectra of compound **14b** in DMSO-d_6.

11-Methyl-7-(*β*-D-ribofuranosyl)pyrimido[5',4':4,5]pyrrolo[3,2-*f*]quinoline (14c)

Parameter	Value
1 Title	Compound 14c (MF-G486)
2 Origin	Bruker BioSpin GmbH
3 Solvent	DMSO
4 Temperature	298.0
5 Number of Scans	32
6 Acquisition Date	2023-12-12T03:36:00
7 Spectrometer Frequency	499.98
8 Nucleus	1H

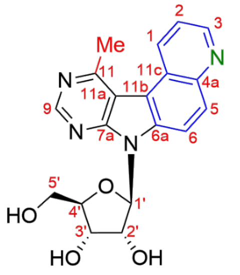

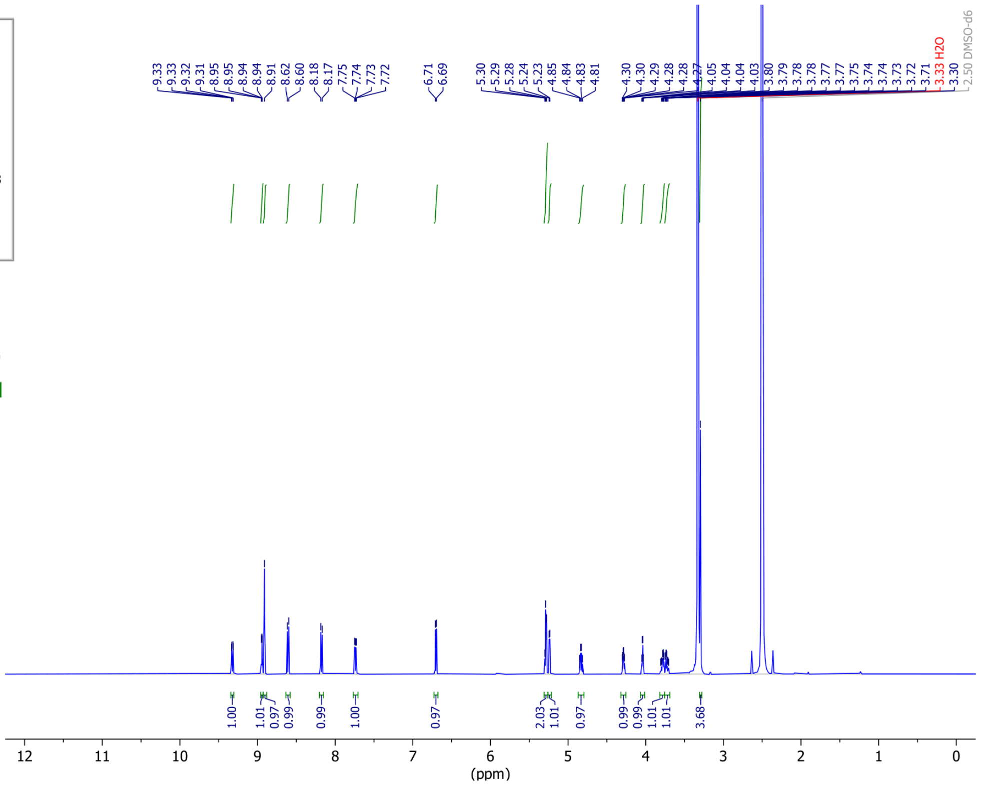

Parameter	Value
1 Title	Compound 14c (MF-G486)
2 Origin	Bruker BioSpin GmbH
3 Solvent	DMSO
4 Temperature	298.0
5 Number of Scans	4096
6 Acquisition Date	2023-12-12T08:33:00
7 Spectrometer Frequency	125.73
8 Nucleus	13C

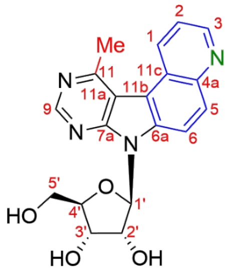

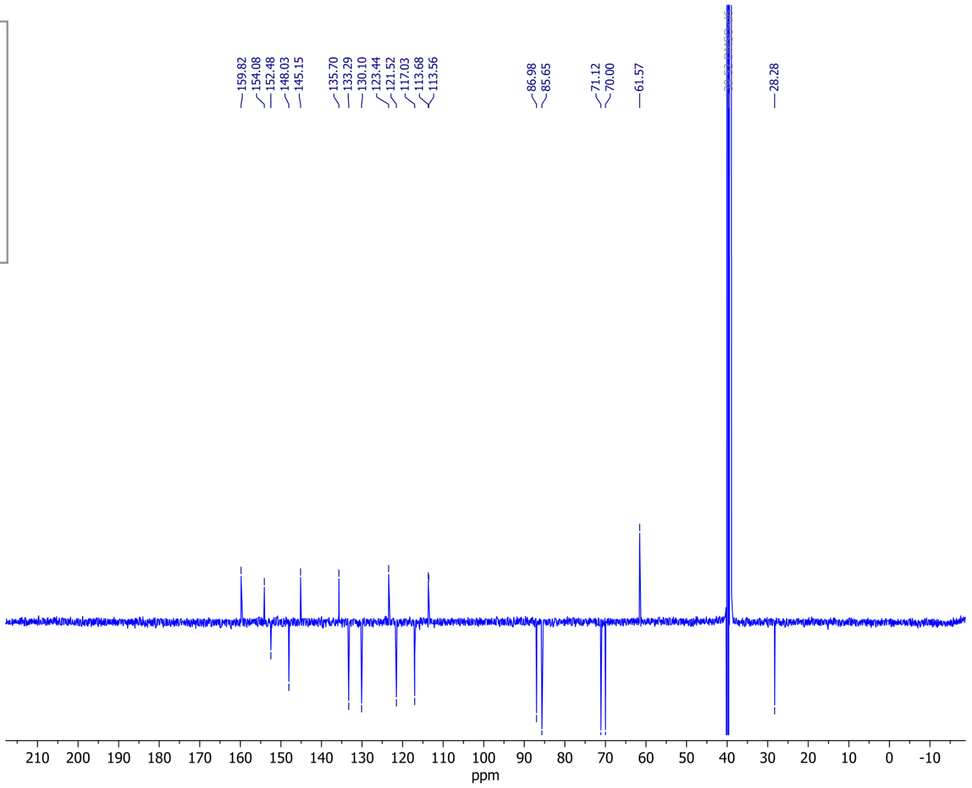

Figure S19. ^{1}H and ^{13}C NMR spectra of compound **14c** in DMSO-d_6.

11-(*N*,*N*-dimethylamino)-7-(*β*-D-ribofuranosyl)pyrimido[5',4':4,5]pyrrolo[3,2-*f*]quinoline (14d)

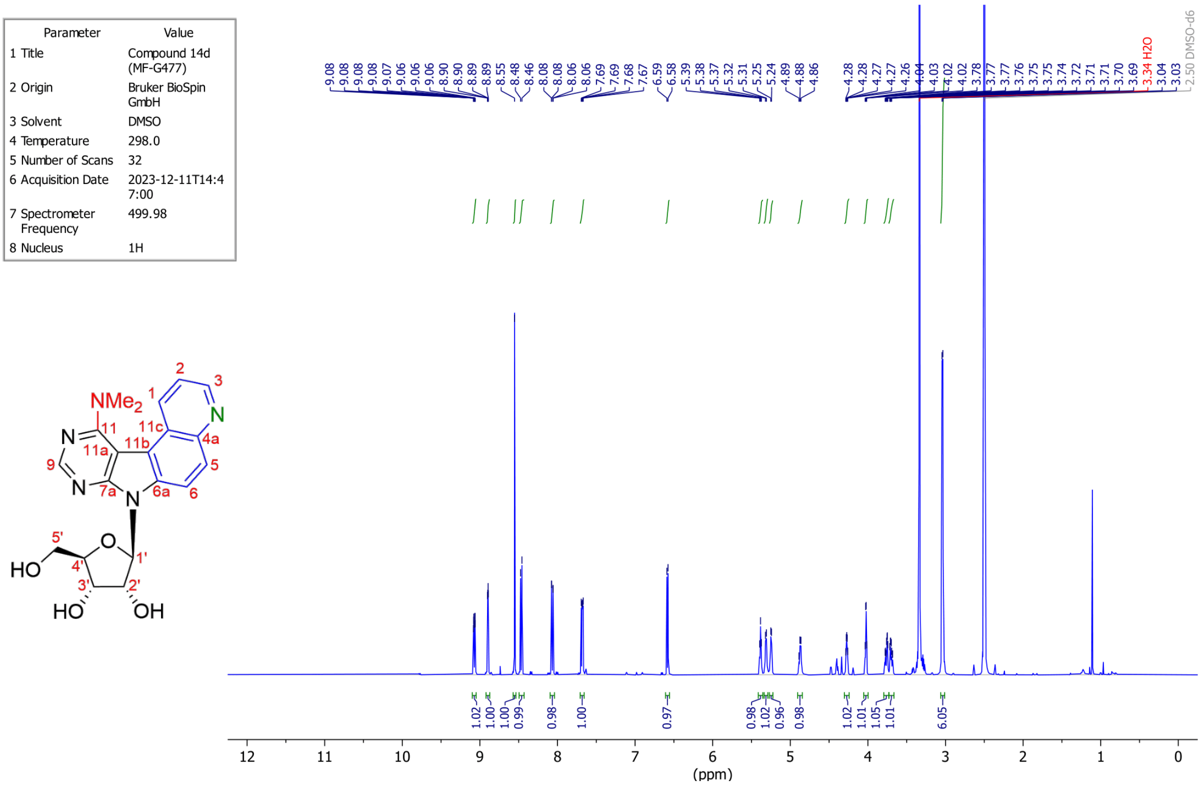

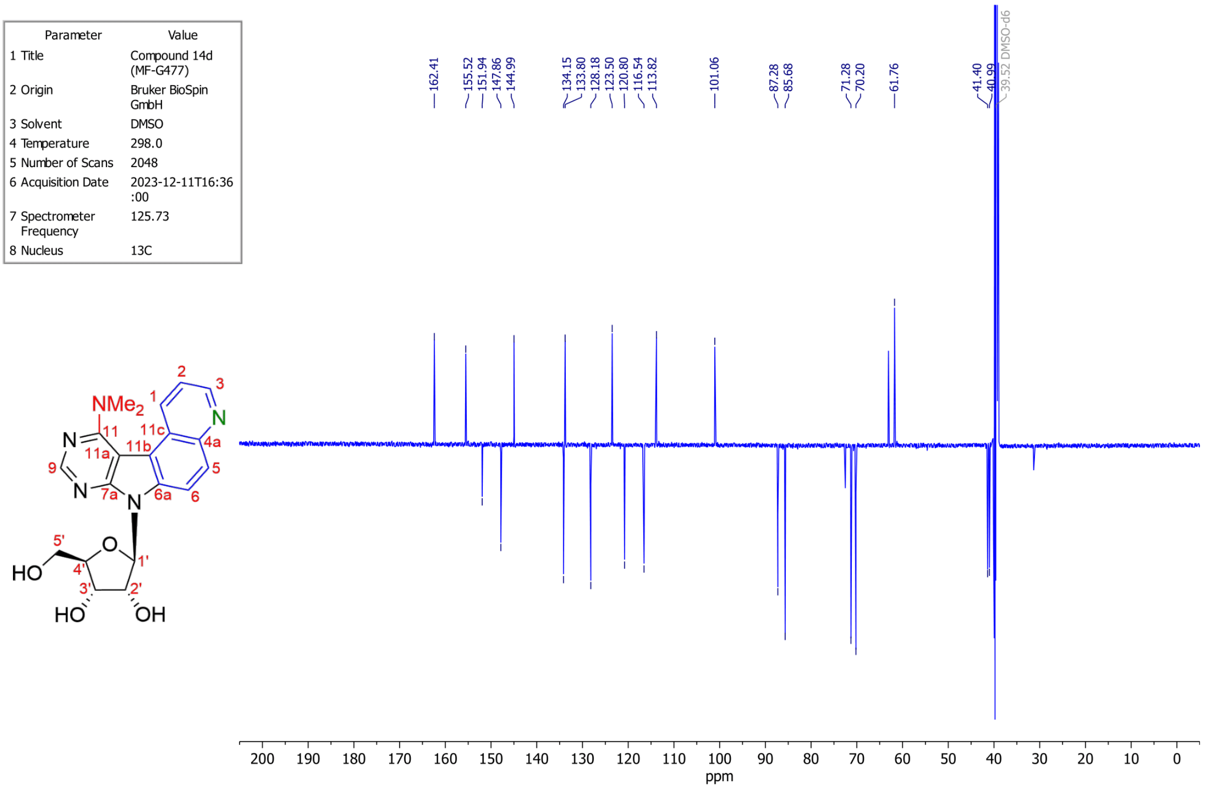

Figure S20. ^{1}H and ^{13}C NMR spectra of compound **14d** in DMSO-d_6.

11-Amino-7-(*β*-D-ribofuranosyl)pyrimido[5',4':4,5]pyrrolo[3,2-*f*]quinoline (14e)

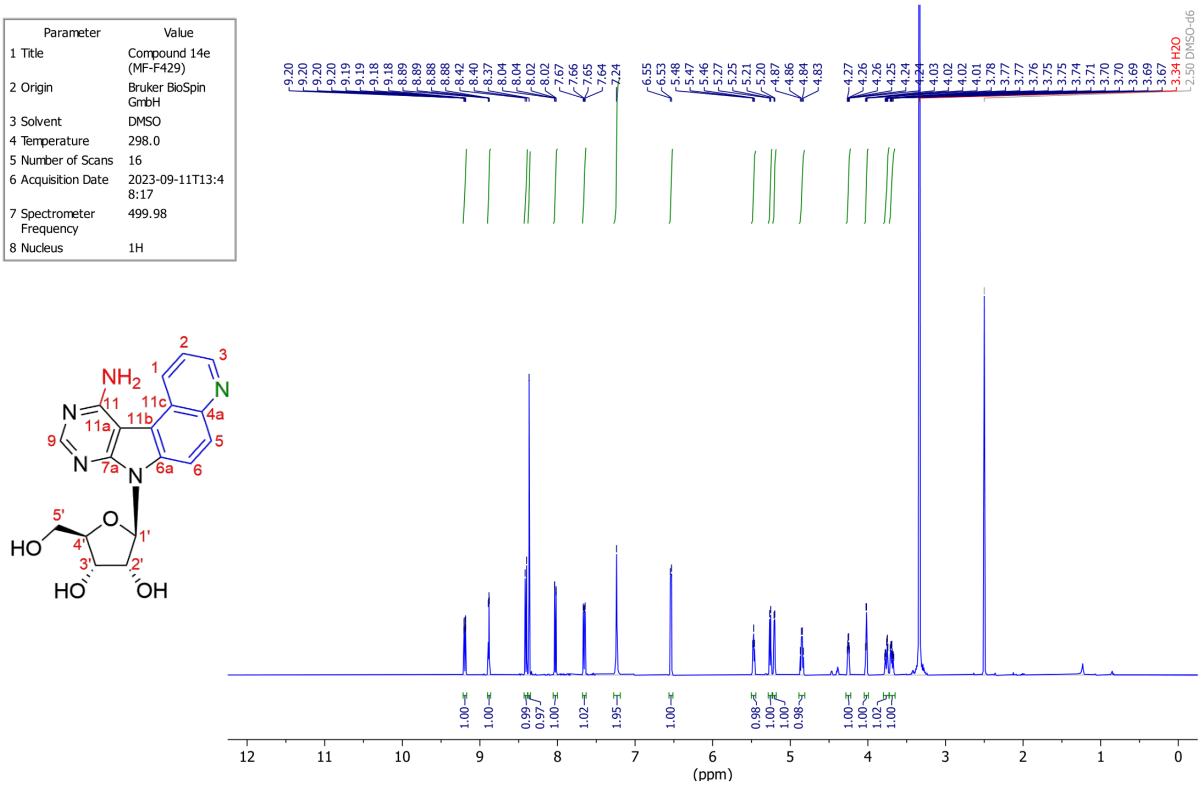

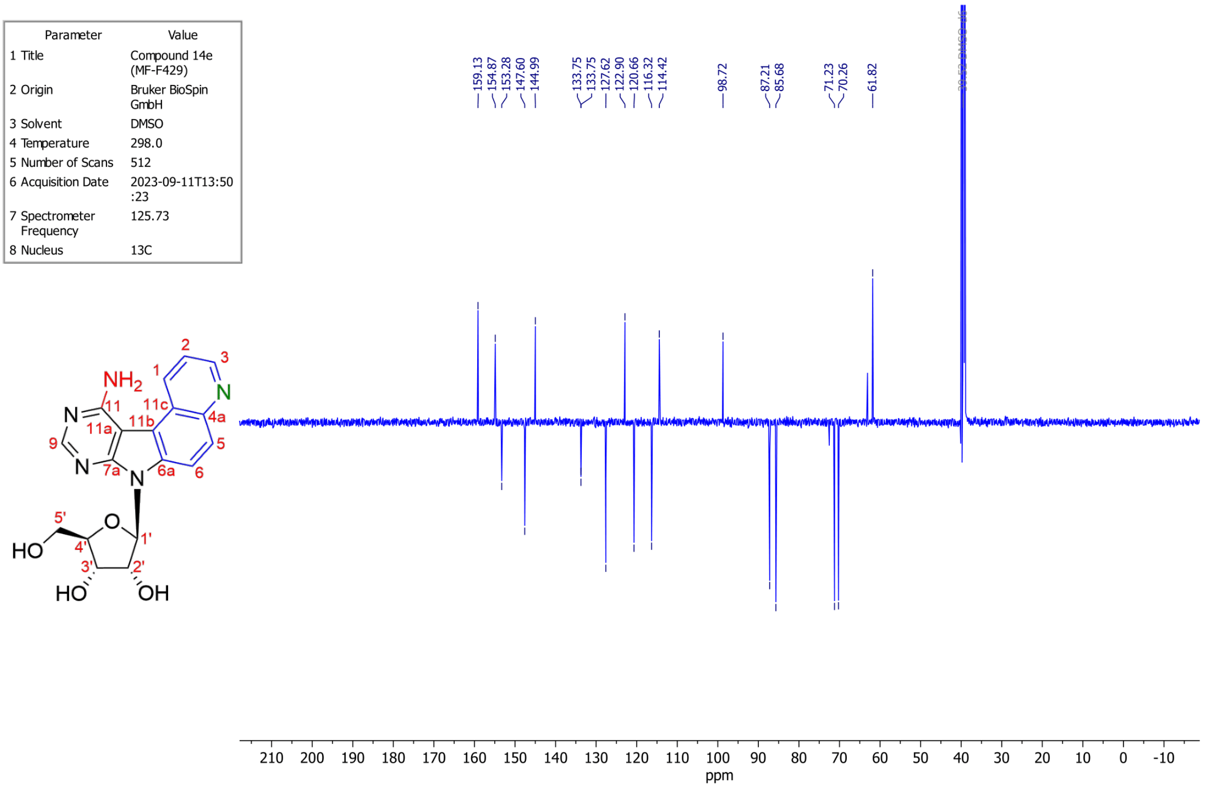

Figure S21. ^{1}H and ^{13}C NMR spectra of compound **14e** in DMSO-d_6.

11-Methoxy-7-(β-D-ribofuranosyl)pyrimido[5',4':4,5]pyrrolo[3,2-*f*]quinoline (14f)

Parameter	Value
1 Title	Compound 14f (MF-E382)
2 Origin	Bruker BioSpin GmbH
3 Solvent	DMSO
4 Temperature	298.2
5 Number of Scans	32
6 Acquisition Date	2022-11-29T11:04:45
7 Spectrometer Frequency	600.13
8 Nucleus	1H

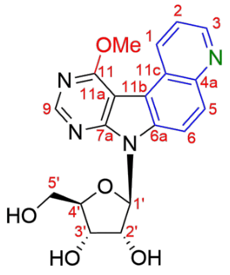

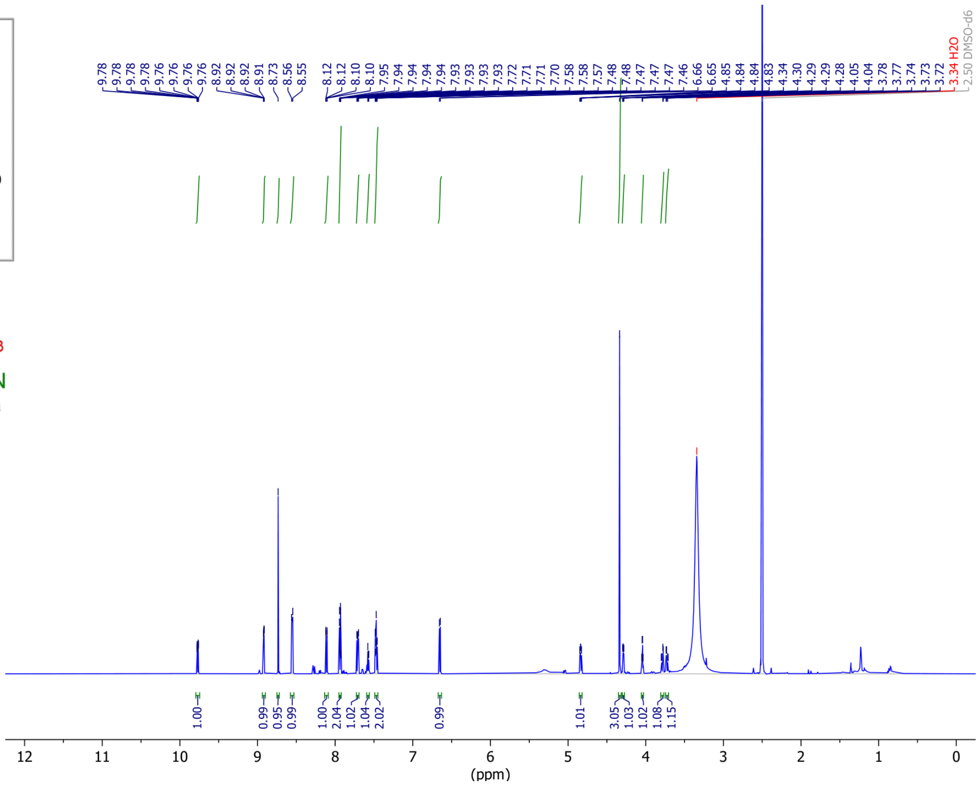

Parameter	Value
1 Title	Compound 14f (MF-E382)
2 Origin	Bruker BioSpin GmbH
3 Solvent	DMSO
4 Temperature	298.2
5 Number of Scans	966
6 Acquisition Date	2022-11-29T11:51:32
7 Spectrometer Frequency	150.92
8 Nucleus	13C

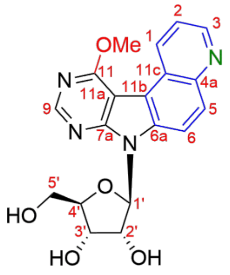

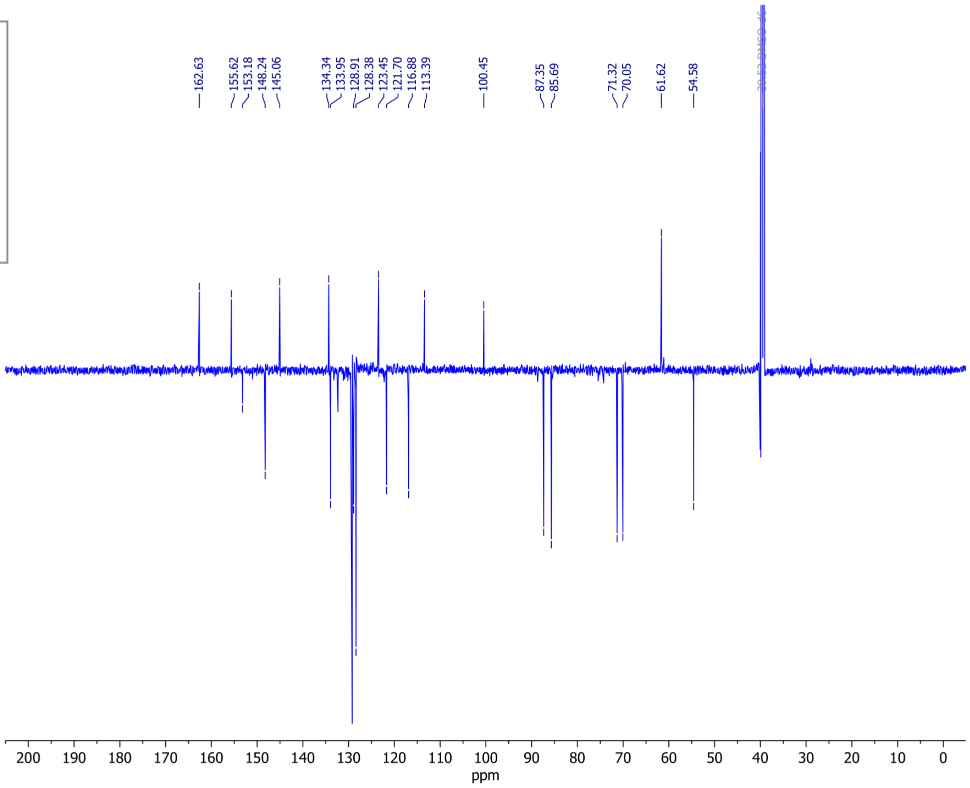

Figure S22. ^{1}H and ^{13}C NMR spectra of compound **14f** in DMSO-d_6.

11-Methylthio-7-(*β*-D-ribofuranosyl)pyrimido[5',4':4,5]pyrrolo[3,2-*f*]quinoline (14g)

Parameter	Value
1 Title	Compound 14g (MF-G482)
2 Origin	Bruker BioSpin GmbH
3 Solvent	DMSO
4 Temperature	298.0
5 Number of Scans	32
6 Acquisition Date	2023-12-11T21:35:00
7 Spectrometer Frequency	499.98
8 Nucleus	1H

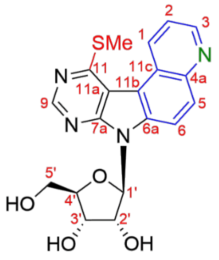

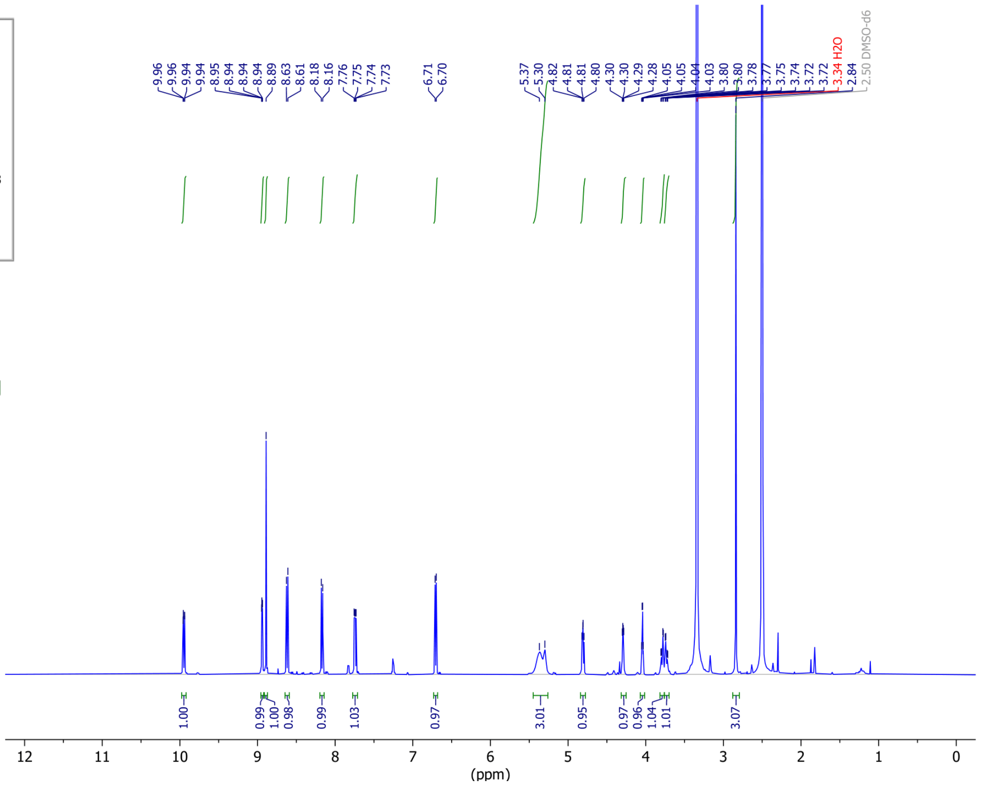

Parameter	Value
1 Title	Compound 14g (MF-G482)
2 Origin	Bruker BioSpin GmbH
3 Solvent	DMSO
4 Temperature	298.0
5 Number of Scans	2048
6 Acquisition Date	2023-12-11T23:22:00
7 Spectrometer Frequency	125.73
8 Nucleus	13C

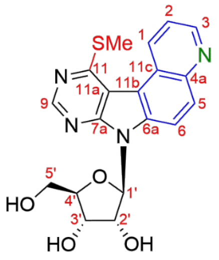

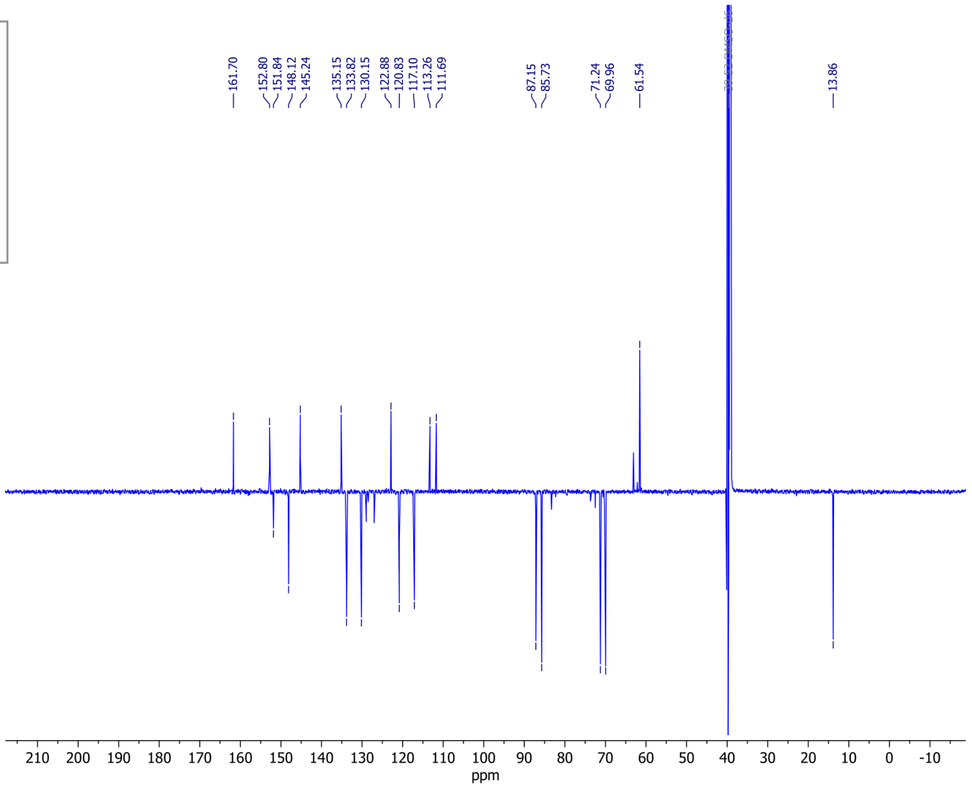

Figure S23. ^{1}H and ^{13}C NMR spectra of compound **14g** in DMSO-d_6.

11-Amino-7-(β-D-ribofuranosyl)pyrimido[5',4':4,5]pyrrolo[3,2-*f*]quinoline 5'-*O*-Triphosphate Bistriethylammonium Salt (15, A^{Q}TP)

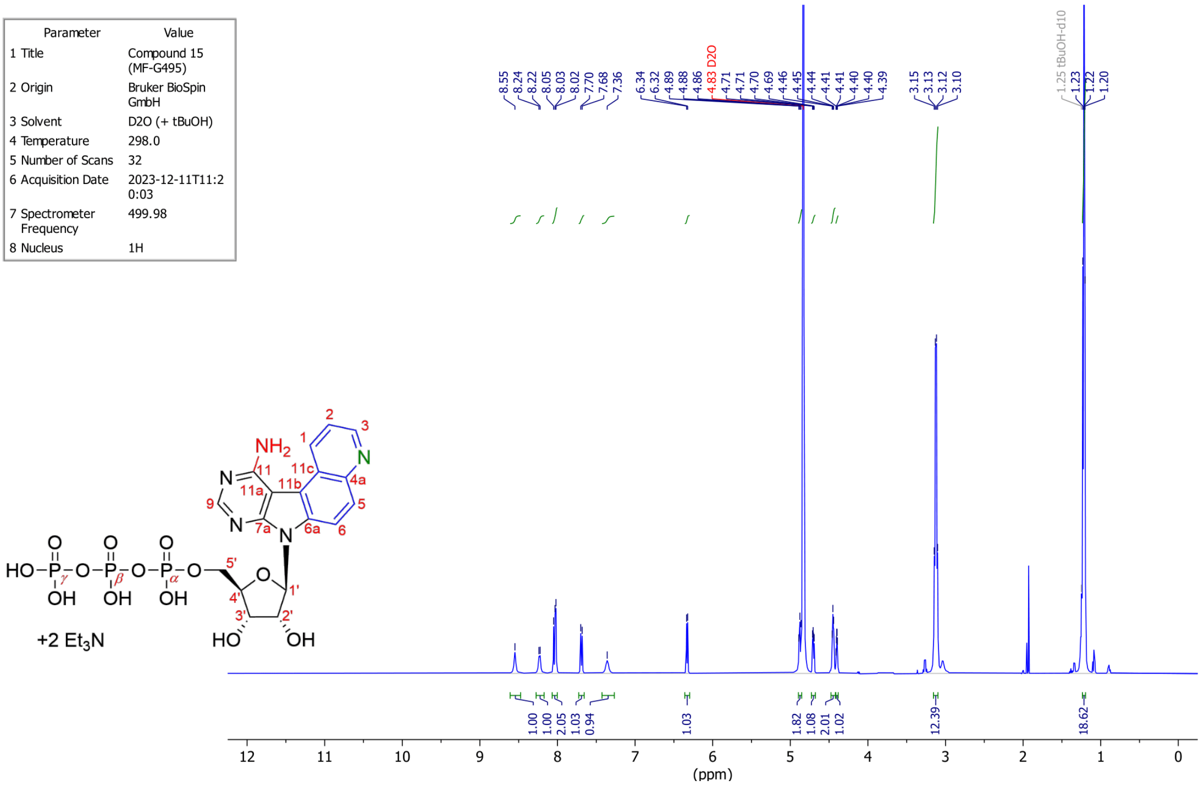

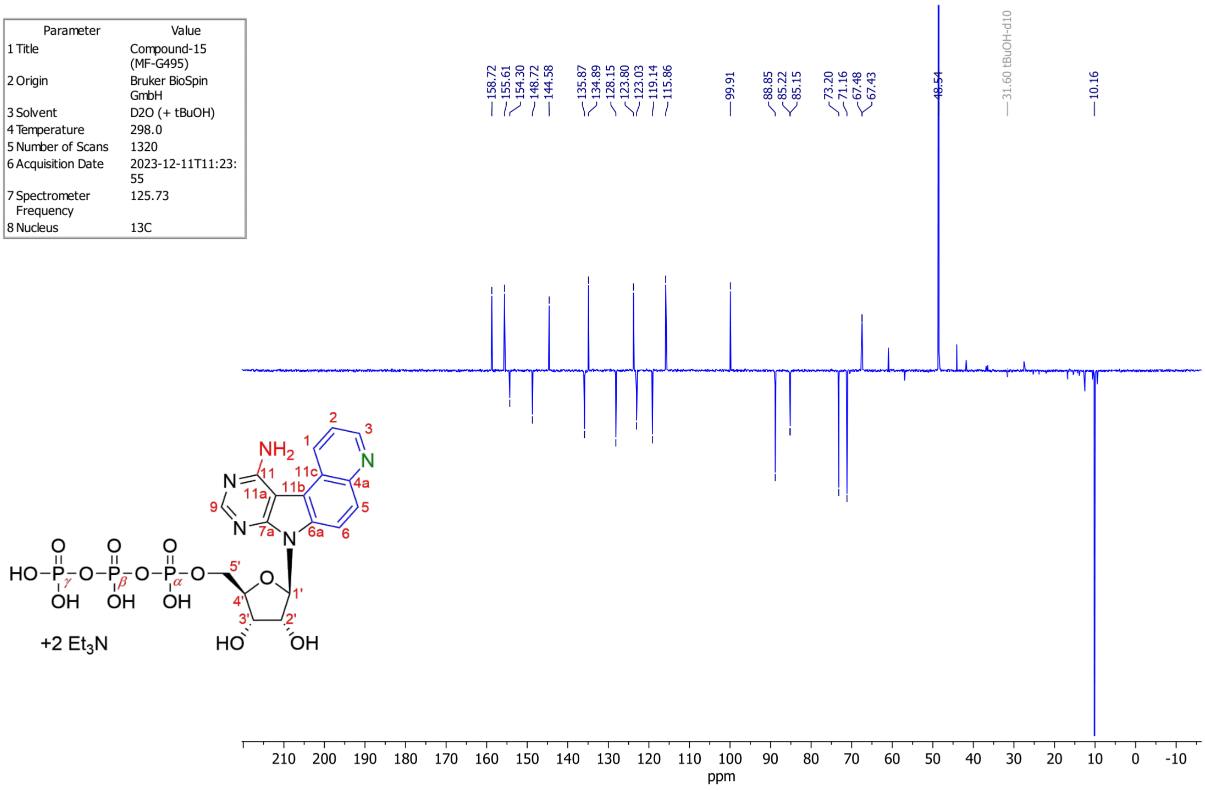

Figure S24. ^{1}H and ^{13}C NMR spectra of compound **15** in D_2O.

Parameter	Value
1 Title	Compound 15 (MF-G495)
2 Origin	Bruker BioSpin GmbH
3 Solvent	D2O (+ tBuOH)
4 Temperature	298.0
5 Number of Scans	32
6 Acquisition Date	2023-12-11T11:11:38
7 Spectrometer Frequency	202.39
8 Nucleus	31P

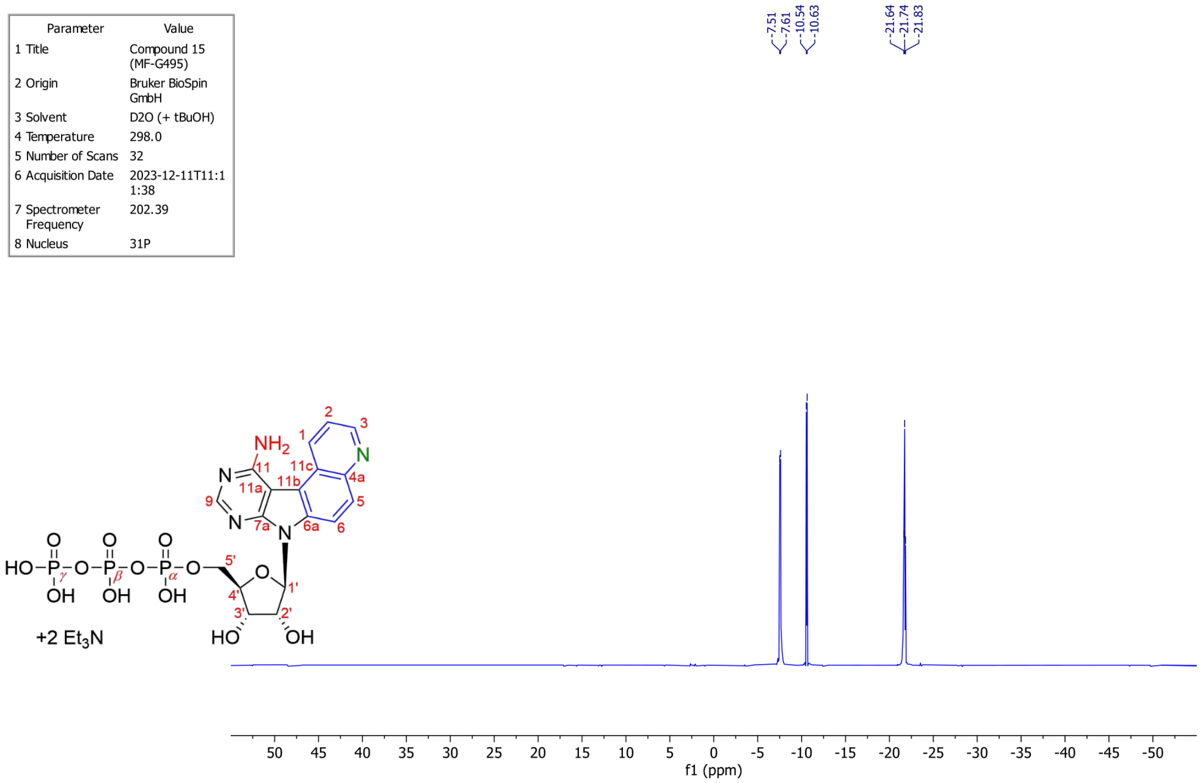

Figure S25. ^{31}P NMR spectrum of compound **15** in D_2O.

6-Chloro-5-(quinolin-5-yl)pyrimidin-4-amine (16)

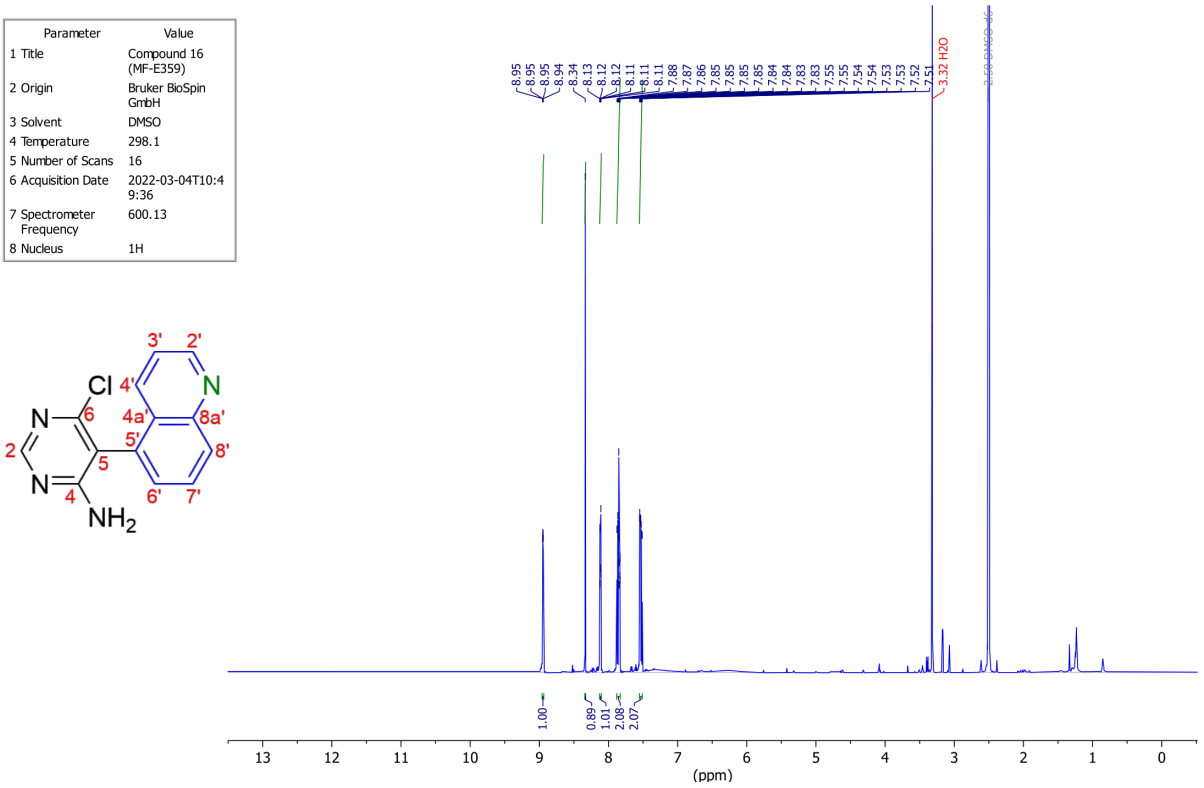

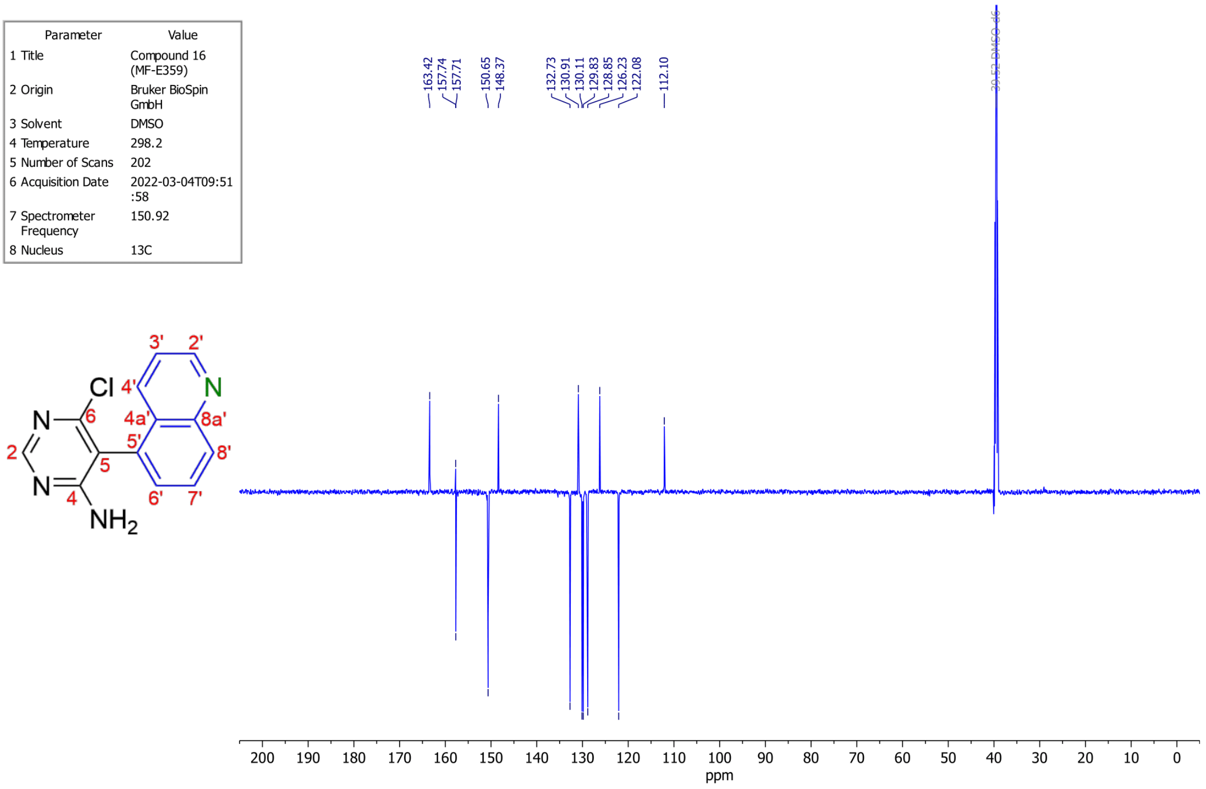

Figure S26. ^{1}H and ^{13}C NMR spectra of compound **16** in DMSO-d_6.

S7. References

1 Fleuti, M.; Bártová, K.; Poštová Slavětínská, L.; Tloušťová, E.; Tichý, M.; Gurská, S.; Pavliš, P.; Džubák, P.; Hajdúch, M.; Hocek, M. Synthesis and Biological Profiling of Pyrazolo-Fused 7-Deazapurine Nucleosides. *J. Org. Chem.* **2020**, *85*, 10539−10551. DOI: 10.1021/acs.joc.0c00928

2 (a) Tichý, M.; Smoleń, S.; Tloušťová, E.; Pohl, R.; Oždian, T.; Hejtmánková, K.; Lišková, B.; Gurská, S.; Džubák, P.; Hajdúch, M.; Hocek, M. Synthesis and Cytostatic and Antiviral Profiling of Thieno-Fused 7-Deazapurine Ribonucleosides. *J. Med. Chem.* **2017**, *60*, 2411–2424. DOI: 10.1021/acs.jmedchem.6b01766; (b) Tokarenko, A.; Lišková, B.; Smoleń, S.; Táborská, N.; Tichý, M.; Gurská, S.; Perlíková, P.; Frydrych, I.; Tloušťová, E.; Znojek, P.; Mertlíková-Kaiserová, H.; Poštová Slavětínská, L.; Pohl, R.; Klepetářová, B.;Khalid, N.-U.-A.; Wenren, Y.; R. Laposa, R. R.; Džubák, P.; Hajdúch, M.; Hocek, M. Synthesis and Cytotoxic and Antiviral Profiling of Pyrrolo- and Furo-Fused 7-Deazapurine Ribonucleosides. *J. Med. Chem.* **2018**, *61*, 9347−9359. DOI: 10.1021/acs.jmedchem.8b01258; (c) Ghosh, K.; Perlíková, P.; Havlíček, V.; Yang, C.; Pohl, R.; Tloušťová, E.; Hodek, J.; Gurská, S.; Džubák, P.; Hajdúch, M.; Hocek, M. Isomeric Naphtho-Fused 7-Deazapurine Nucleosides and Nucleotides: Synthesis, Biological Activity, Photophysical Properties and Enzymatic Incorporation to Nucleic Acids. *Eur. J. Org. Chem.* **2018**, *37*, 5092–5108. DOI: 10.1002/ejoc.201800165; (d) Yang, C.; Poštová Slavětínská, L.; Fleuti, M.; Klepetářová, B.; Tichý, M.; Gurská, S.; Pavliš, P.; Džubák, P.; Hajdúch, M.; Hocek, M. Synthesis of Polycyclic Hetero-Fused 7-Deazapurine Heterocycles and Nucleosides through C−H Dibenzothiophenation and Negishi Coupling. *J. Am. Chem. Soc.* **2022**, *144*, 19437−19446. DOI: 10.1021/jacs.2c07517

3 Yang, C.; Pohl, R.; Tichý, M.; Gurská, S.; Pavliš, P.; Džubák, P.; Hajdúch, M.; Hocek, M. Synthesis, Photophysical Properties, and Biological Profiling of Benzothieno-Fused 7-Deazapurine Ribonucleosides. *J. Org. Chem.* **2020**, *85*, 8085–8101. DOI: 10.1021/acs.joc.0c00927

4 Yang, C.; Tichý, M.; Poštová Slavětínská, L.; Vaiedelich, E.; Gurská, S.; Džubák, P.; Hajdúch, M.; Hocek, M. Synthesis and Biological Profiling of Benzofuro-Fused 7-Deazapurine Nucleosides. *Eur. J. Org. Chem.* **2023**, *26*, e202300723. DOI: 10.1002/ejoc.202300723

5 Stokes, B. J.; Jovanović, B.; Dong, H.; Richert, K. J.; Riell, R. D.; Driver, T. G. Rh_2(II)-Catalyzed Synthesis of Carbazoles from Biaryl Azides. *J. Org. Chem.* **2009**, *74*, 3225–3228. DOI: 10.1021/jo9002536

6 Zhou, F.; Liu, S.; Santarsiero, B.; Wink, D. J.; Boudinet, D.; Facchetti, A.; Tom Driver, T. Synthesis and Properties of New N-Heteroheptacenes for Solution-Based Organic Field Effect Transistors. *Chem. Eur. J.* **2017**, *23*, 12542–12549. DOI: 10.1002/chem.201701966

7 Yang, C.; Pohl, R.; Tichý, M.; Gurská, S.; Pavliš, P.; Džubák, P.; Hajdúch, M.; Hocek, M. Synthesis, Photophysical Properties, and Biological Profiling of Benzothieno-Fused 7-Deazapurine Ribonucleosides. *J. Org. Chem.* **2020**, *85*, 8085–8101. DOI: 10.1021/acs.joc.0c00927

8 Würth, C.; Grabolle, M.; Pauli, J.; Spieles, M.; Resch-Genger, U., Relative and absolute determination of fluorescence quantum yields of transparent samples. *Nat. Protoc.* **2013,** *8*, 1535–1550. DOI: 10.1038/nprot.2013.087

9 Eaton, D. F. Reference materials for fluorescence measurement. *Pure Appl. Chem.* **1988**, *60*, 1107–1114. DOI: 10.1351/pac198860071107

10 Kozma, I. Z.; Krok, P.; Riedle, E. Direct measurement of group-velocity mismatch and derivation of the refractive index dispersion for a variety of solvents in the ultraviolet. *J. Opt. Soc. Am. B* **2005**, *22*, 1479–1485. DOI: 10.1364/AO.12.000555
Values retrieved from: Polyanskiy, M. RefractiveIndex.INFO – refractive index database. https://refractiveindex.info/?shelf=organic&book=methanol&page=Kozma (accessed 2024-02-28)

11 Hale, G. M.; Querry, M. R. Optical constants of water in the 200 nm to 200 μm wavelength region. *Appl. Opt.* **1973**, *12*, 555–563. DOI: 10.1364/JOSAB.22.001479
Values retrieved from: Polyanskiy, M. RefractiveIndex.INFO – refractive index database. https://refractiveindex.info/?shelf=3d&book=liquids&page=water (accessed 2024-02-28).
